# Development of New Thiophene-Containing Triaryl Pyrazoline Derivatives as PI3Kγ Inhibitors

**DOI:** 10.3390/molecules27082404

**Published:** 2022-04-08

**Authors:** Bing Yang, Bo Zhang, Qun Zhao, Jin Li, Yujun Shi

**Affiliations:** 1 School of Chemistry and Chemical Engineering, Nantong University, Nantong 226019, China; qunzhao@ntu.edu.cn; 2 State Key Laboratory of Pharmaceutical Biotechnology, Nanjing University, Nanjing 210023, China; dg1930048@smail.nju.edu.cn; 3 National and Local Joint Engineering Research Center for Mineral Salt Deep Utilization, Key Laboratory for Palygorskite Science and Applied Technology of Jiangsu Province, Huaiyin Institute of Technology, Huai’an 223003, China

**Keywords:** PI3K inhibition activity, thiophene moiety, pyrazoline derivatives, molecular docking

## Abstract

A series of new thiophene-containing triaryl pyrazoline derivatives, **3a**–**3t**, were synthesized and evaluated regarding PI3K inhibition activity and anti-tumor potency based on a trial of introducing significant moieties, including pyrazoline and thiophene, and simplifying the parallel ring structures. Most of the tested compounds indicated potent PI3K inhibitory potency, with this series of compounds showing more potency for PI3Kγ than PI3Kα. The top hit **3s** seemed more potent than the positive control **LY294002** on inhibiting PI3Kγ (IC_50_ values: 0.066 μM versus 0.777 μM) and more selective from PI3Kα (Index values: 645 versus 1.74). It could be inferred that the combination of *para*- and *meta*-, as well as the modification of the electron-donating moieties, led to the improvement in potency. The anti-proliferation inhibitory activity and the enzymatic inhibition potency indicated consistent tendencies. The top hit **3s** could inhibit the phosphorylation of Akt by inhibiting PI3K through the PI3K-Akt-mTOR pathway. The molecular docking simulation indicated that the binding pattern of **3s** into PI3Kγ was preferable than that of PI3Kα, with more hydrogen bond, more π-involved interactions, and fewer π-sulfur interactions. The information in this work is referable for the further development of selective inhibitors for specific isoforms of PI3K.

## 1. Introduction

Cancer is a great risk for human health in modern society and is associated with several major signaling pathways such as the Mitogen-Activated Protein Kinases (MAPK) family pathway, Akt pathway and so on [1,2,3]. Along with the Akt pathway, the upstream node and phosphatidylinositol 3-kinases (PI3Ks) have actually drawn more attention due to their essential roles in various cellular procedures, including motility, differentiation, proliferation, growth and intracellular trafficking [4,5,6]. In humans, PI3Ks and phosphatase and tensin homologue (PTEN) are reported as a complementary pair in the interconversion of the second messenger phosphatidylinositol 3,4,5-triphosphate (PIP3) and its precursor phosphatidylinositol (4,5) diphosphate (PIP2) [7,8,9]. As a primary process, PI3Ks can convert PIP2 into PIP3 and mediate downstream biological events, whereas PTEN can transform PIP3 into PIP2 as a feedback course. Based on the deeper understanding of the biochemistry, the PI3K family could be classified into three classes (I, II, and III) according to the features in structures and the specificity of substrates [10,11,12]. Among them, Class I PI3Ks have been associated more often with cancer therapies in recent decades and have become a research hotspot for investigators [13,14,15]. In detail, there are four known isoforms, named PI3Kα, β, γ and δ, in Class I PI3Ks which are usually assembled from the p110 catalytic subunit and p85 regulating subunit [16,17,18,19]. PI3Kα and PI3Kβ show common expression in many sites, while PI3Kδ and PI3Kγ are merely located in epithelial cells and the central nervous and hematopoietic systems [20,21,22]. Since Class I PI3Ks has been reported to participate in oncogenesis frequently, the development of corresponding inhibitors seems quite important in seeking the potential approaches for treating cancer.

**Figure 1:**
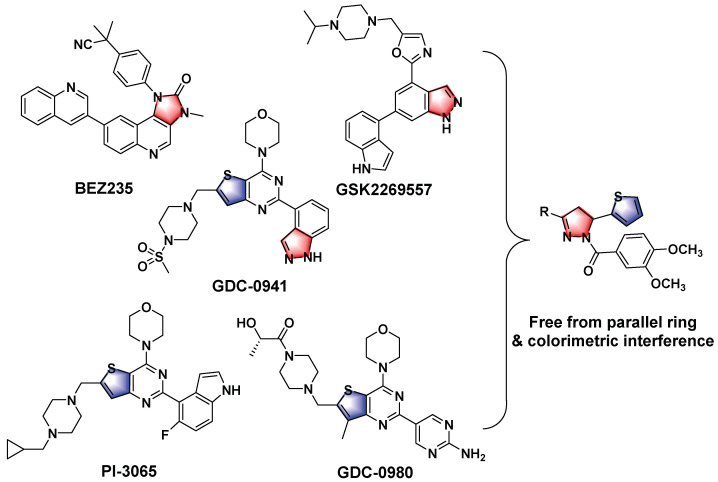
The design concept of this work, introducing the reported functional groups and simplifying the parallel ring structures.

A variety of PI3K inhibitors have been identified and exploited, among which several representatives have been promoted to early clinical trials [23,24,25]. A typical example was Wortmannin, which could inhibit both PI3K and polo-like kinase 1 (PLK1) with high potency. However, its antibiotic-like structure seemed difficult to modify. Other reported representatives included BEZ235, GSK2269557, GDC-0941, GDC-0980, and PI-3065 [26,27,28,29,30]. Except for the candidates in clinical trials, there were also other distinctive backbones such as chromeno [4,3-*c*] pyrazol-4(2*H*)-one [31,32]. From the above-mentioned cases, we identified the functional groups to organize the design concept in this work. As shown in Figure 1, the pyrazoline moiety was derived from BEZ235, GSK2269557 and GDC-0941; the thiophene was selected from GDC-0941, GDC-0980 and PI-3065; while the parallel ring structures were simplified to eliminate the colorimetric interference in the previous reports [31,32]. The parts similar to vanillic acyl were also reported in PI3K inhibition studies [33].

There are few reports about the thiophene derivatives containing triaryl pyrazoline. In 2020, thiazolyl-3-(2-thienyl)-5-aryl-2-pyrazoline derivatives were synthesized through acrylation and nucleophilic addition reaction after the addition and condensation reaction of chalcone and thiosemicarbazide [34]. Research results showed the compound, ethyl-4-methyl-2-(5-phenyl-3-(thiophen-2-yl)-4,5-dihydropyrazol-1-yl)thiazole-5-carboxylate, had potent anti-tumor activity against HCT-116 cells and CACO-2 cells and no cytotoxicity to BHK cells [34]. In our previous work, some thiophene derivatives containing the characteristic 3,4,5-trimethoxy phenyl structure were prepared from chalcone [35]. Most compounds could inhibit the tubulin polymerization. The top hit compound had strong anti-proliferation potency for MCF-7 cells, HepG2 cells and HeLa cells. This compound also exhibited inhibitory activity on VEGFR2. In this work, the synthesized new series **3a**–**3t** were evaluated on the PI3K inhibition activity and anti-tumor potency.

## 2. Results and Discussion

### 2.1. Chemistry

**Scheme 1:**
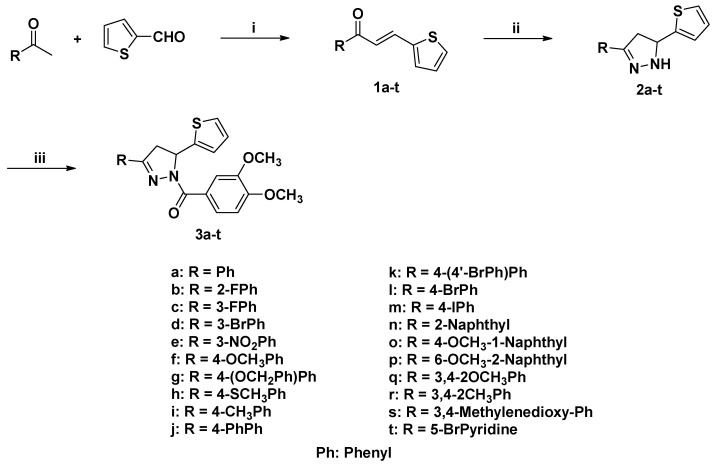
Synthesis of compounds **3a**–**3t**. Reagents and conditions: (i) 5% NaOH, EtOH, 0 °C-r.t., overnight; (ii) N_2_H_4_·H_2_O, EtOH, reflux, 5 h; (iii) 3,4-dimethoxybenzoic acid, EDC·HCl, HOBt, dichloromethane, r.t.

Synthesis of compounds **3a**–**t** is shown in Scheme 1. Chalcones **1a**–**1t**, which were obtained from the condensation reactions of 2-thiophenecarboxaldehyde with acetophenones [35,36], were reacted with hydrazine hydrate to give the pyrazoline intermediates **2a**–**2t** according to the previous procedure reported [35,37]. Acylation of pyrazolines **2a**–**2t** with 3,4-dimethoxybenzoic acid catalyzed by EDC·HCl and HOBt produced target compounds **3a**–**3t [38]**.

### 2.2. Biological Acitivity

#### 2.2.1. PI3K Inhibition Assay

Initially, we directly tested the inhibition potency of the synthesized compounds on PI3K by using competitive fluorescence polarization kinase kits, as mentioned in previous investigations [31,32]. The results were organized in Table 1. According to the significance and research maturity of the isoforms as referenced, the PI3Kα and PI3Kγ isoforms were preliminarily included here. The origin ligand of the protein crystal complex **LY294002** was selected as the positive control. General speaking, the majority of the tested compounds indicated potent PI3K inhibitory potency, which set the basis for discussing the structure–activity relationship (SAR).

The results inferred that this series of compounds were more potent for PI3Kγ than PI3Kα. Compared with the positive control **LY294002**, the top hits indicated even better selectivity towards PI3Kγ. The top three hits for PI3Kγ were **3s**, **3q** and **3f** with corresponding half inhibitory concentration (IC_50_) values of 0.066 μM, 0.430 μM and 0.570 μM, respectively. Regarding the data of the selectivity index values, **LY294002** was calculated as 1.74 (0.777/0.447), while **3s**, **3q** and **3f** were calculated as 645 (42.600/0.066), 12.2 (5.240/0.430), and 46.7 (26.600/0.570), respectively. Accordingly, preliminarily, we could regard the most potent compound **3s** as a potential candidate for selectively inhibiting PI3Kγ. Subsequently, the potency for inhibiting PI3Kγ was associated with the substituent group. First, if extending along with the symmetry axis from the linking bond (*para*-substitute) was an acceptable strategy, the tested compounds indicated the tendency that the steric factor was essential here and the suitable length seemed between one and two benzene rings. The corresponding data suggested that **3f** (4-OCH_3_) was among the top hits (IC_50_ = 0.570 μM); other one-ring compounds (**3a**, **3i**, **3l**, **3m**) indicated attractive potency (IC_50_ < 10 μM), except for **3h** (IC_50_ = 18.100 μM), while two-ring compounds (**3g**, **3j**, **3k**) demonstrated no potential for further investigation (IC_50_ > 25 μM). Then, the extending deviation from the symmetry axis (*para*- to *meta*- to *othro*-) was checked. Whether introducing *othro*-substitute alone (**3b**) was beneficial was not clear (IC_50_ = 4.430 μM), while merely introducing *meta*-substitute (**3c**, **3d**, **3e**) seemed preferable for improving the potency (IC_50_ = 4.210 μM, 2.220 μM, 1.120 μM, respectively). For a single substitute, it seemed that the electron-donating substitute (**3f**, **3i**) resulted in better effects than the electron-withdrawing (**3l**, **3m**) on the *para*-position, except for **3h**. It was not obvious on the *meta*-position. Moreover, in the cases of multi-substituent, parallel or heterocyclic ring, the situation could be abstracted into the combination of *para*- and *meta*- (**3n**, **3p**, **3q**, **3r**, **3s**), *meta*- and *ortho*- (**3o**), *para*- and *othro*- (**3t**). It seemed that only the combination of *para*- and *meta*- led to the improvement of the potency, while, in detail, within a relatively narrow space, the tiny modification of the electron-donating moieties could bring better inhibitory activity. Afterwards, regarding inhibiting PI3Kα, the tendency could also be hinted according to the data in Table 1. The tendency could be summarized more simply. Except for **3e** with nitro at the *meta*-position, the bulky moieties showed better potency than the single-substituent ones. Among the tested compounds, **3p** indicated the potential to act as a pan blocker of both PI3Kγ than PI3Kα. In previous reports, many of the potential inhibitors were pan blockers or more potent for PI3Kα [31,32]. In this work, the top hits could be comparable in the potency, which was preferable for PI3Kγ instead. For the PI3K family, both pan blockers and specific inhibitors were essential; however, PI3Kγ seemed the most significant in the tumorigenesis, therefore the selectivity towards PI3Kγ in this work was meaningful.

#### 2.2.2. The Anti-Proliferation Assay

In this section, the anti-proliferation activity of the synthesized compounds **3a**–**3t** was evaluated. Herein, HeLa (human epithelial cervical cancer cell line), HepG2 (human hepatoellular carcinoma cell line) and L02 (human normal hepatocyte line) cells were used. According to the affection on L02 cells, most of the tested compounds were low-toxic. For example, the cytotoxicity of the top hit **3s** was much lower than the control (CC_50_ values: 181.000 μM vs. 51.300 μM). Basically, many of the compounds in this series could inhibit the growth of HeLa and HepG2 cells effectively. The anti-proliferation inhibitory activity and the enzymatic inhibition potency indicated the consistent tendencies; the SAR of the observed anti-proliferation activity was also similar to that of the enzymatic inhibition, especially that of PI3Kγ inhibition; thus, the cellular inhibition might be caused by the effect on PI3K. In comparison with different cell lines, this series showed better potency on HepG2 than HeLa. This result agreed with the fact that the PI3K-associated metabolism was more frequent in liver than in model cancer cells (HeLa was from human epithelial cervical cancer but now more universal). Thus, HepG2 cells were selected to conduct further investigations at the protein level.

#### 2.2.3. Western Blot

**Figure 2:**
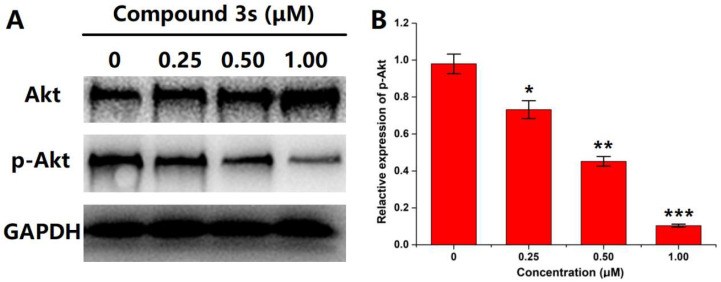
(**A**) The expression levels of Akt, phosphorylated Akt (p-Akt) and GAPDH in HepG2 cells incubated with various concentrations of compound **3s** (0, 0.25, 0.50, 1.00 μM); (**B**) Quantitation analysis of the p-Akt level. Data were expressed as Mean ± SD (*n* = 3). * *p* < 0.05, ** *p* < 0.01 and *** *p* < 0.005 vs. the control.

For the evaluation of PI3Kγ activity, the downstream signal of PI3Kγ should be checked. Commonly, in the PI3K-Akt-mTOR pathway, the activation of PI3K mediated the phosphorylation of Akt and then triggered the following biological events. Herein, Western blot was conducted to visualize the expression levels of Akt and p-Akt (phosphorylated Akt) in HepG2 cells after incubation with various concentrations of the top hit **3s** (Figure 2). GAPDH was used as the internal reference to guarantee cell status. Along with the increase in **3s** concentration, the p-Akt level indicated an obvious decrease, whereas the Akt level almost remained unchanged (even slightly increased). This meant that compound **3s** has the potential to cause dose-dependent blocking on the PI3K-mediated Akt phosphorylation.

### 2.3. Molecular Docking Simulation

**Figure 3:**
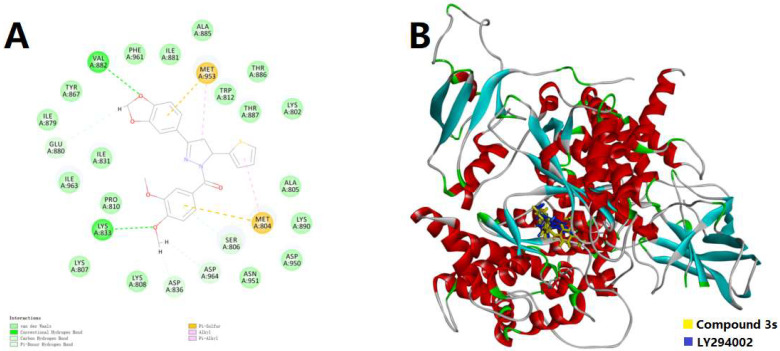

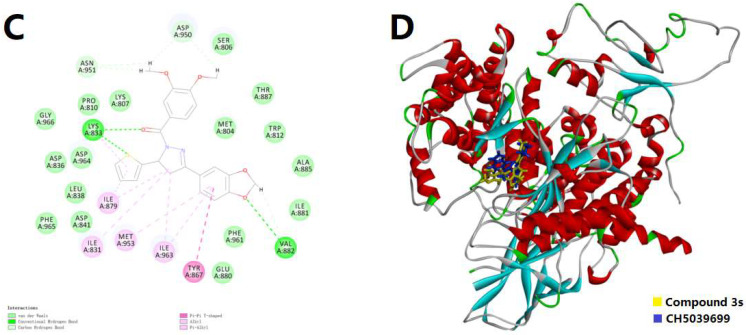
(**A**) The 2D docking model of compound **3s** into PI3Kα; (**B**) The 3D docking models of **3s** and **LY294002** into PI3Kα; (**C**) The 2D docking model of compound **3s** into PI3Kγ; (**D**) The 3D docking models of **3s** and **CH5039699** into PI3Kγ. The H-bonds were displayed as green dotted lines. The π-related interactions were shown as pink (π-π or π-alkyl) and yellow (π-sulfur) dotted lines.

Since the biological assay confirmed that **3s** could act as a potential candidate for the selective inhibition of PI3Kγ, the molecular docking simulation was conducted to visualize the possible binding pattern of **3s** into the active site of PI3Kα (PDB code: 1E7V) and PI3Kγ (PDB code: 3APF), respectively. The binding conformations were also compared with the original ligands, **LY294002** and **CH5039699**, respectively. The maps of the binding patterns in both 2D and 3D are depicted in Figure 3. In Figure 3A, in the binding site of PI3Kα, **3s** indicated possible hydrogen bonds with the key residues Val882 and Lys883, as well as π-alkyl and π-sulfur interactions with key residues Met804 and Met953. It was notable that several important interactions relied on sulfur, which was not typical and might be not strong enough for binding. This result inferred that the selectivity for PI3Kγ from PI3Kα might be caused by the affection of thiophene moiety. In Figure 3B, compound **3s** could mimic part of the structural feature of **LY294002**, but the stretched dimethoxyphenyl (DMP) group differed from the control, thus leading to a new possible hydrogen bond. However, for embedding into the deeper site of PI3Kα, **LY294002** seemed more suitable than **3s**. On the other hand, with the binding pattern of **3s** into PI3Kγ, as displayed in Figure 3C, there might be possible hydrogen bonds with the key residues Val882 and Lys883, π-π interactions with Tyr867, as well as π-alkyl interactions with Ile831, Ile879, Met953 and Ile963. Compared with the binding pattern into PI3Kα, this time one more hydrogen bond and much more π-involved interactions were introduced, while the π-sulfur interactions were not involved. Both the thiophene moiety and the aryl group might participate in the interaction of hydrogen bonds. These results agreed with the selectivity for PI3Kγ from PI3Kα because the tendency of interaction was stronger (Interaction Energy: −58.1403 Kcal/mol < −46.0220 Kcal/mol). Furthermore, in Figure 3D, **3s** could mimic almost the whole molecule of **CH5039699**, except for a slight difference between the DMP group of **3s** and the parallel ring of **CH5039699**. This result agreed with the initial design of simplifying the parallel ring structures and might lead to further inspiration on the modification of such inhibitory candidates, because, in this work, the potency of **3s** was attractive.

## 3. Materials and Methods

### 3.1. Materials and Apparatus

Solvents and reagents with analytical grade were used without further purification. Chromatographic purification of products was performed on silica gel (200–300 mesh). Melting points were determined on a micro melting point apparatus (SGW X-4B, Shanghai, China) and uncorrected. ^1^H and ^13^C NMR spectra were recorded on a Bruker AVANCE III HD 400M spectrometer (Zurich, Switzerland) with Tetramethyl silane (TMS) as the internal standard. Chemical shifts (δ) were reported in ppm (parts per million) with respect to TMS. HRMS (High Resolution Mass Spectrometry) analyses were carried out using an AB Sciex TripleTOF 4600 System mass spectrometer (Framingham, MA, USA) with an ESI (electrospray ionization) source.

### 3.2. Chemical Syntheses

Chalcones **1a**–**1t** were prepared from 2-furaldehyde and acetophenone derivatives through aldol reaction and dehydration reaction, then compounds **2a**–**2t** were synthesized from chalcones **1a**–**1t** and hydrazine hydrate by addition and condensation reaction, and, finally, the target molecules (**3a**–**3t**) were obtained from acylation reaction of the compounds **2a**–**2t**.

#### 3.2.1. Synthesis of Chalcones (**1a**–**1t**)

Chalcones **1a**–**1t** were obtained according to the procedure as described previously [35,36]. 2-furaldehyde (10 mmol) was dissolved in EtOH (15 mL) and stirred, and then to the above solution was added an acetophenone derivative (10 mmol). The resulting mixture was cooled at 0 °C and 8 mL of 5% NaOH water solution was added drop wise. After reaction for 24 h, the resulting solution was poured into ice water (50 g) and stirred. The crude product precipitated from the solution, filtrated and washed with cold water and ethanol. The pure chalcones (**1a**–**1t**) were obtained from water–ethanol by recrystallization, yield 70–98%.

#### 3.2.2. Synthesis of 3-Aryl-5-(thiophen-2-yl)-4,5-dihydro-1*H*-pyrazole Derivatives (**2a**–**2t**)

To a solution of chalcone (**1a**–**1t**, 2 mmol) in EtOH (10 mL) was added hydrazine hydrate (1 mL). The mixture was then stirred at reflux for 5 h then cooled. The reaction mixture was stored at −20 °C overnight. The precipitate (**2a**–**2t**) formed was filtered off, washed with petroleum ether and was used immediately without any further purification.

#### 3.2.3. Synthesis of (3,4-Dimethoxyphenyl)(3-aryl-5-(thiophen-2-yl)-4,5-dihydro-1*H*-pyrazol-1-yl)methanone Derivatives(**3a**–**3t**)

The precipitate (**2a**–**2t**, 1 mmol) from the last step was dissolved in dichloromethane (10 mL). 3,4-dimethoxybenzoic acid (2 mmol), EDC∙HCl (3 mmol) and HOBt (1.2 mmol) were added, and the reaction was stirred for 48 h at room temperature. After removal of dichloromethane in a vacuum, the product was purified by flash chromatography to obtain the target compound **3a**–**3t** (Table 2), yield 30.7–63.6%. NMR Spectra and HRMS analytical data see Supplementary Materials.

#### 3.2.4. (3,4-Dimethoxyphenyl)(3-phenyl-5-(thiophen-2-yl)-4,5-dihydro-1*H*-pyrazol-1-yl)methanone (**3a**)

Light yellow solid. Yield: 34.1%. m.p. 141–143 °C. ^1^H NMR (400 MHz, CDCl_3_) δ 3.39 (dd, 1H, *J* = 17.6, 4.4 Hz), 3.77 (dd, 1H, *J* = 17.2, 11.2 Hz), 3.94 (d, 6H, *J* = 8.0 Hz), 6.15 (dd, 1H, *J* = 11.6, 4.8 Hz), 6.92–6.96 (m, 2H), 7.11 (d, 1H, *J* = 3.6 Hz), 7.20 (d, 1H, *J* = 4.8 Hz), 7.41–7.44 (m, 3H), 7.69 (s, 1H), 7.49 (t, 2H, *J* = 4.0 Hz), 7.83 (d, 1H, *J* = 8.4 Hz). ^13^C NMR (100 MHz, CDCl_3_) δ 41.2, 56.1, 57.0, 109.9, 113.5, 124.6, 124.8, 125.1, 126.3, 126.9 (d, *J* = 14.0 Hz), 128.9, 130.6, 131.5, 144.5, 148.1, 151.6, 154.5, 165.8. HRMS (ESI-TOF) *m*/*z*: [M + H]^+^ Calcd. for C_22_H_21_N_2_O_3_S 393.1267, Found 393.1266.

#### 3.2.5. (3,4-Dimethoxyphenyl)(3-(2-fluorophenyl)-5-(thiophen-2-yl)-4,5-dihydro-1*H*-pyrazol-1-yl)methanone (**3b**)

Light yellow solid. Yield: 40.2%. m.p. 118–120 °C. ^1^H NMR (400 MHz, CDCl_3_) δ 3.50 (ddd, 1H, *J* = 16.8, 6.0, 2.4 Hz), 3.82–3.94 (m, 7H), 6.13 (dd, 1H, *J* = 11.6, 4.8 Hz), 6.90–6.96 (m, 2H), 7.11–7.21 (m, 4H), 7.38–7.44 (m, 1H), 7.70 (d, 1H, *J* = 2.0 Hz), 7.80–7.91 (m, 2H). ^13^C NMR (100 MHz, CDCl_3_) δ 43.4 (d, *J* = 7.0 Hz), 56.1, 56.9 (d, *J* = 3.0 Hz), 109.9, 113.6, 116.7, 116.9, 119.6 (d, *J* = 11.0 Hz), 124.6 (d, *J* = 3.0 Hz), 124.8, 125.1, 126.2, 127.0, 129.0 (d, *J* = 3.0 Hz), 132.1 (d, *J* = 9.0 Hz), 144.5, 148.2, 151.3 (d, *J* = 3.0 Hz), 151.7, 160.1, 162.6, 165.8. HRMS (ESI-TOF) *m*/*z*: [M + H]^+^ Calcd. for C_22_H_20_FN_2_O_3_S 411.1173, Found 411.1171.

#### 3.2.6. (3,4-Dimethoxyphenyl)(3-(3-fluorophenyl)-5-(thiophen-2-yl)-4,5-dihydro-1*H*-pyrazol-1-yl)methanone (**3c**)

Light yellow solid. Yield: 58.3%. m.p. 50–51 °C. ^1^H NMR (400 MHz, CDCl_3_) δ 3.35 (dd, 1H, *J* = 17.6, 4.8 Hz), 3.70–3.77 (m, 1H), 3.92–3.95 (m, 6H), 6.16 (dd, 1H, *J* = 11.6, 4.8 Hz), 6.91–6.95 (m, 2H), 7.10–7.15 (m, 2H), 7.20 (dd, 1H, *J* = 5.2, 1.2 Hz), 7.36–7.42 (m, 1H), 7.45–7.48 (m, 2H), 7.20 (d, 1H, *J* = 2.0 Hz), 7.80 (dd, 1H, *J* = 8.4, 2.0 Hz). ^13^C NMR (100 MHz, CDCl_3_) δ 41.1, 56.1 (d, *J* = 1.0 Hz), 57.1, 109.9, 113.4 (t, *J* = 13.0 Hz), 117.4, 117.6, 122.6 (d, *J* = 3.0 Hz), 124.5, 124.9, 125.1, 126.0, 127.0, 130.5 (d, *J* = 8.0 Hz), 133.6 (d, *J* = 8.0 Hz), 144.2, 148.2, 151.7, 153.4 (d, *J* = 3.0 Hz), 161.7, 164.2, 165.9. HRMS (ESI-TOF) *m*/*z*: [M + H]^+^ Calcd. for C_22_H_20_FN_2_O_3_S 411.1173, Found 411.1176.

#### 3.2.7. (3-(3-Bromophenyl)-5-(thiophen-2-yl)-4,5-dihydro-1*H*-pyrazol-1-yl)(3,4-dimethoxyphenyl)methanone (**3d**)

Light yellow solid. Yield: 32.1%. m.p. 124–126 °C. ^1^H NMR (400 MHz, CDCl_3_) δ 3.34 (dd, 1H, *J* = 17.6, 4.4 Hz), 3.72 (dd, 1H, *J* = 17.6, 11.6 Hz), 3.94 (d, 6H, *J* = 7.2 Hz), 6.15 (dd, 1H, *J* = 11.6, 4.8 Hz), 6.89–6.95 (m, 2H), 7.10 (d, 1H, *J* = 3.2 Hz), 7.20 (dd, 1H, *J* = 4.8, 0.8 Hz), 7.26–7.31 (m, 1H), 7.55 (dd, 1H, *J* = 8.0, 0.8 Hz), 7.62–7.68 (m, 2H), 7.79 (dd, 1H, *J* = 8.4, 1.6 Hz), 7.90 (t, 1H, *J* = 1.2 Hz). ^13^C NMR (100 MHz, CDCl_3_) δ 41.0, 56.0 (d, *J* = 3.0 Hz), 57.1, 109.9, 113.5, 123.1, 124.5, 124.9, 125.1, 125.3, 126.0, 127.0, 129.6, 130.4, 133.4 (d, *J* = 17.0 Hz), 144.2, 148.1, 151.7, 153.0, 165.8. HRMS (ESI-TOF) *m*/*z*: [M + H]^+^ Calcd. for C_22_H_20_BrN_2_O_3_S 471.0373, Found 471.0371.

#### 3.2.8. (3,4-Dimethoxyphenyl)(3-(3-nitrophenyl)-5-(thiophen-2-yl)-4,5-dihydro-1*H*-pyrazol-1-yl)methanone (**3e**)

Yellow solid. Yield: 40.8%. m.p. 76–78 °C. ^1^H NMR (400 MHz, CDCl_3_) δ 3.42 (dd, 1H, *J* = 17.6, 4.8 Hz), 3.81 (dd, 1H, *J* = 17.6, 11.6 Hz), 3.96 (s, 6H), 6.22 (dd, 1H, *J* = 11.6, 4.4 Hz), 6.90–7.00 (m, 2H), 7.12 (d, 1H, *J* = 3.2 Hz), 7.21 (d, 1H, *J* = 4.8 Hz), 7.62 (t, 1H, *J* = 8.0 Hz), 7.69 (d, 1H, *J* = 1.6 Hz), 7.79 (dd, 1H, *J* = 8.4, 2.0 Hz), 8.06 (d, 1H, *J* = 7.6 Hz), 8.27 (dd, 1H, *J* = 8.0, 1.2 Hz), 8.55 (s, 1H). ^13^C NMR (100 MHz, CDCl_3_) δ 41.0, 56.1 (d, *J* = 1.0 Hz), 57.5, 110.0, 113.5, 121.5, 124.6, 124.8, 125.0, 125.3, 125.8, 127.1, 130.1, 132.2, 133.3, 144.0, 148.3, 148.7, 152.0, 152.1, 165.9. HRMS (ESI-TOF) *m*/*z*: [M + H]^+^ Calcd. for C_22_H_20_N_3_O_5_S 438.1118, Found 438.1129.

#### 3.2.9. (3,4-Dimethoxyphenyl)(3-(4-methoxyphenyl)-5-(thiophen-2-yl)-4,5-dihydro-1*H*-pyrazol-1-yl)methanone (**3f**)

White solid. Yield: 51.1%. m.p. 53–55 °C. ^1^H NMR (400 MHz, CDCl_3_) δ 3.34 (dd, 1H, *J* = 17.2, 4.4 Hz), 3.72 (dd, 1H, *J* = 17.6, 11.6 Hz), 3.85 (s, 3H), 3.94 (d, 6H, *J* = 6.8 Hz), 6.12 (dd, 1H, *J* = 11.2, 4.4 Hz), 6.91–7.00 (m, 4H), 7.10 (d, 1H, *J* = 3.2 Hz), 7.19 (d, 1H, *J* = 4.8 Hz), 7.67–7.70 (m, 3H), 7.83 (dd, 1H, *J* = 8.4, 1.6 Hz). ^13^C NMR (100 MHz, CDCl_3_) δ 41.3, 55.5, 56.1, 56.9, 109.9, 113.6, 114.3, 124.1, 124.5, 124.7, 125.0, 126.5, 126.9, 128.4, 144.7, 148.1, 151.5, 154.4, 161.6, 165.5. HRMS (ESI-TOF) *m*/*z*: [M + H]^+^ Calcd. for C_23_H_23_N_2_O_4_S 423.1373, Found 423.1382.

#### 3.2.10. (3-(4-(Benzyloxy)phenyl)-5-(thiophen-2-yl)-4,5-dihydro-1*H*-pyrazol-1-yl)(3,4-dimethoxyphenyl)methanone (**3g**)

Light yellow solid. Yield: 54.5%. m.p. 60–62 °C. ^1^H NMR (400 MHz, CDCl_3_) δ 3.33 (dd, 1H, *J* = 17.6, 4.4 Hz), 3.71 (dd, 1H, *J* = 17.2, 11.2 Hz), 3.93 (d, 6H, *J* = 6.4 Hz), 5.11 (s, 2H), 6.12 (dd, 1H, *J* = 11.2, 4.4 Hz), 6.89–6.95 (m, 2H), 6.99–7.03 (m, 2H), 7.10 (d, 1H, *J* = 2.8 Hz), 7.19 (dd, 1H, *J* = 5.2, 1.2 Hz), 7.32–7.45 (m, 5H), 7.67–7.70 (m, 3H), 7.83 (dd, 1H, *J* = 8.4, 1.6 Hz). ^13^C NMR (100 MHz, CDCl_3_) δ 41.2, 56.0, 56.8, 70.2, 109.8, 113.5, 115.2, 124.2, 124.6 (d, *J* = 21.0 Hz), 124.9, 126.4, 126.9, 127.5, 128.4 (d, *J* = 14.0 Hz), 128.8, 136.4, 144.6, 148.1, 151.5, 154.4, 160.6, 165.5. HRMS (ESI-TOF) *m*/*z*: [M + H]^+^ Calcd. for C_29_H_27_N_2_O_4_S 499.1686, Found 499.1704.

#### 3.2.11. (3-(4-((λ^1^-Sulfanyl)-λ^5^-methyl) phenyl)-5-(thiophen-2-yl)-4,5-dihydro-1*H*-pyrazol-1-yl) (3,4-dimethoxyphenyl)methanone (**3h**)

Yellow solid. Yield: 50.7%. m.p. 63–65 °C. ^1^H NMR (400 MHz, CDCl_3_) δ 2.50 (s, 3H), 3.33 (dd, 1H, *J* = 17.6, 4.4 Hz), 3.72 (dd, 1H, *J* = 17.6, 11.6 Hz), 3.93 (d, 6H, *J* = 8.0 Hz), 6.13 (dd, 1H, *J* = 11.2, 4.4 Hz), 6.89–6.94 (m, 2H), 7.09 (d, 1H, *J* = 3.2 Hz), 7.18 (d, 1H, *J* = 4.8 Hz), 7.25 (d, 1H, *J* = 8.8 Hz), 7.65 (t, 3H, *J* = 8.4 Hz), 7.81 (dd, 1H, *J* = 8.4, 1.2 Hz). ^13^C NMR (100 MHz, CDCl_3_) δ 15.2, 41.1, 56.0, 56.9, 109.8, 113.5, 124.5, 124.7, 125.0, 125.9, 126.2, 127.0 (d, *J* = 15.0 Hz), 127.8, 142.2, 144.4, 148.1, 151.5, 154.2, 165.6. HRMS (ESI-TOF) *m*/*z*: [M + H]^+^ Calcd. for C_23_H_23_N_2_O_3_S_2_ 439.1145, Found 439.1135.

#### 3.2.12. (3,4-Dimethoxyphenyl)(5-(thiophen-2-yl)-3-(p-tolyl)-4,5-dihydro-1*H*-pyrazol-1-yl)methanone (**3i**)

Light yellow solid. Yield: 45.4%. m.p. 165–167 °C. ^1^H NMR (400 MHz, CDCl_3_) δ 2.40 (s, 3H), 3.36 (dd, 1H, *J* = 17.6, 4.4 Hz), 3.73 (dd, 1H, *J* = 17.6, 11.6 Hz), 3.91 (d, 6H, *J* = 7.6 Hz), 6.13 (dd, 1H, *J* = 11.2, 4.4 Hz), 6.91–6.95 (m, 2H), 7.10 (d, 1H, *J* = 3.2 Hz), 7.18–7.26 (m, 3H), 7.63–7.70 (m, 3H), 7.82–7.84 (m, 1H). ^13^C NMR (100 MHz, CDCl_3_) δ 21.6, 41.2, 56.0 (d, *J* = 1.0 Hz), 56.9, 109.8, 113.5, 124.6 (d, *J* = 18.0 Hz), 125.0, 126.4, 126.8 (d, *J* = 14.0 Hz), 128.7, 129.6, 140.9, 144.6, 148.1, 151.5, 154.7, 165.6. HRMS (ESI-TOF) *m*/*z*: [M + H]^+^ Calcd. for C_23_H_23_N_2_O_3_S 407.1424, Found 407.1430.

#### 3.2.13. (3-([1,1’-Biphenyl]-4-yl)-5-(thiophen-2-yl)-4,5-dihydro-1*H*-pyrazol-1-yl)(3,4-dimethoxyphenyl)methanone (**3j**)

Light yellow solid. Yield: 40.2%. m.p. 154–156 °C. ^1^H NMR (400 MHz, CDCl_3_) δ 3.42 (dd, 1H, *J* = 17.6, 4.4 Hz), 3.80 (dd, 1H, *J* = 17.6, 11.2 Hz), 3.95 (d, 6H, *J* = 5.6 Hz), 6.17 (dd, 1H, *J* = 11.6, 4.8 Hz), 6.90–6.96 (m, 2H), 7.13 (d, 1H, *J* = 3.2 Hz), 7.21 (d, 1H, *J* = 4.8 Hz), 7.37–7.49 (m, 3H), 7.62–7.70 (m, 5H), 7.81–7.86 (m, 3H). ^13^C NMR (100 MHz, CDCl_3_) δ 41.2, 56.1, 57.0, 109.9, 113.5, 124.6, 124.8, 125.1, 126.3, 127.0, 127.2 (d, *J* = 12.0 Hz), 127.6, 128.1, 129.1, 130.3, 140.2, 143.3, 144.5, 148.1, 151.6, 154.3, 165.7. HRMS (ESI-TOF) *m*/*z*: [M + H]^+^ Calcd. for C_28_H_25_N_2_O_3_S 469.1580, Found 469.1578.

#### 3.2.14. (3-(4’-Bromo-[1,1’-biphenyl]-4-yl)-5-(thiophen-2-yl)-4,5-dihydro-1*H*-pyrazol-1-yl)(3,4-dimethoxyphenyl)methanone (**3k**)

Light yellow solid. Yield: 39.3%. m.p. 75–77 °C. ^1^H NMR (400 MHz, CDCl_3_) δ 3.41 (dd, 1H, *J* = 17.6, 4.8 Hz), 3.79 (dd, 1H, *J* = 17.6, 11.6 Hz), 3.95 (d, 6H, *J* = 7.2 Hz), 6.17 (dd, 1H, *J* = 11.2, 4.4 Hz), 6.92–6.96 (m, 2H), 7.12 (d, 1H, *J* = 3.2 Hz), 7.20 (dd, 1H, *J* = 5.2, 0.8 Hz), 7.48 (d, 2H, *J* = 8.4 Hz), 7.60 (dd, 4H, *J* = 11.6, 8.4 Hz), 7.69 (d, 1H, *J* = 2.0 Hz), 7.80–7.85 (m, 3H). ^13^C NMR (100 MHz, CDCl_3_) δ 41.2, 56.1, 57.1, 109.9, 113.6, 122.4, 124.6, 124.8, 125.1, 126.3, 127.0, 127.4 (d, *J* = 6.0 Hz), 128.7, 130.8, 132.2, 139.1, 142.0, 144.5, 148.2, 151.7, 154.0, 165.7. HRMS (ESI-TOF) *m*/*z*: [M + H]^+^ Calcd. for C_28_H_24_BrN_2_O_3_S 547.0686, Found 547.0681.

#### 3.2.15. (3-(4-Bromophenyl)-5-(thiophen-2-yl)-4,5-dihydro-1*H*-pyrazol-1-yl)(3,4-dimethoxyphenyl)methanone (**3l**)

Light yellow solid. Yield: 47.9%. m.p. 71–73 °C. ^1^H NMR (400 MHz, CDCl_3_) δ 3.34 (dd, 1H, *J* = 17.6, 4.8 Hz), 3.73 (dd, 1H, *J* = 17.6, 11.6 Hz), 3.93 (d, 6H, *J* = 11.6 Hz), 6.14 (dd, 1H, *J* = 11.6, 4.8 Hz), 6.91–6.95 (m, 2H), 7.10 (d, 1H, *J* = 3.2 Hz), 7.20 (dd, 1H, *J* = 4.8, 0.8 Hz), 7.53–7.63 (m, 5H), 7.79 (dd, 1H, *J* = 8.4, 2.0 Hz). ^13^C NMR (100 MHz, CDCl_3_) δ 41.0, 56.1, 57.1, 109.9, 113.4, 124.5, 124.8 (d, *J* = 3.0 Hz), 125.1, 126.1, 127.0, 128.2, 130.4, 132.1, 144.3, 148.1, 151.7, 153.4, 165.8. HRMS (ESI-TOF) *m*/*z*: [M + H]^+^ Calcd. for C_22_H_20_BrN_2_O_3_S 471.0373, Found 471.0370.

#### 3.2.16. (3,4-Dimethoxyphenyl)(3-(4-iodophenyl)-5-(thiophen-2-yl)-4,5-dihydro-1*H*-pyrazol-1-yl)methanone (**3m**)

Light yellow solid. Yield: 30.7%. m.p. 69–71 °C. ^1^H NMR (400 MHz, CDCl_3_) δ 3.31 (dd, 1H, *J* = 17.6, 4.4 Hz), 3.71 (dd, 1H, *J* = 17.2, 11.2 Hz), 3.92 (d, 6H, *J* = 11.2 Hz), 6.13 (dd, 1H, *J* = 11.6, 4.8 Hz), 6.90–6.94 (m, 2H), 7.09 (d, 1H, *J* = 3.2 Hz), 7.19 (dd, 1H, *J* = 5.2, 0.8 Hz), 7.44 (d, 2H, *J* = 8.4 Hz), 7.63 (d, 1H, *J* = 0.8 Hz), 7.74–7.79 (m, 3H). ^13^C NMR (100 MHz, CDCl_3_) δ 40.9, 56.0, 57.1, 96.8, 109.9, 113.5, 124.5, 124.8, 125.1, 126.1, 126.9, 128.2, 130.9, 138.0, 144.3, 148.1, 151.7, 153.5, 165.7. HRMS (ESI-TOF) *m*/*z*: [M + H]^+^ Calcd. for C_22_H_20_IN_2_O_3_S 519.0234, Found 519.0211.

#### 3.2.17. (3,4-Dimethoxyphenyl)(3-(naphthalen-2-yl)-5-(thiophen-2-yl)-4,5-dihydro-1*H*-pyrazol-1-yl)methanone (**3n**)

Light yellow solid. Yield: 43.0%. m.p. 149–151 °C. ^1^H NMR (400 MHz, CDCl_3_) δ 3.53 (dd, 1H, *J* = 17.6, 4.8 Hz), 3.84–3.97 (m, 7H), 6.20 (dd, 1H, *J* = 11.6, 4.8 Hz), 6.92–7.00 (m, 2H), 7.14 (d, 1H, *J* = 3.6 Hz), 7.21 (d, 1H, *J* = 4.8 Hz), 7.51–7.57 (m, 2H), 7.73 (d, 1H, *J* = 1.6 Hz), 7.85–7.89 (m, 4H), 7.98 (s, 1H), 8.03 (dd, 1H, *J* = 8.8, 1.6 Hz). ^13^C NMR (100 MHz, CDCl_3_) δ 41.2, 56.1, 57.1, 110.0, 113.6, 123.4, 124.6, 124.8, 125.1, 126.3, 127.0, 127.5 (d, *J* = 5.0 Hz), 128.0, 128.6, 128.7, 129.1, 133.1, 134.3, 144.5, 148.2, 151.7, 154.6, 165.8. HRMS (ESI-TOF) *m*/*z*: [M + H]^+^ Calcd. for C_26_H_23_N_2_O_3_S 443.1424, Found 443.1408.

#### 3.2.18. (3,4-Dimethoxyphenyl)(3-(4-methoxynaphthalen-1-yl)-5-(thiophen-2-yl)-4,5-dihydro-1*H*-pyrazol-1-yl)methanone (**3o**)

Yellow solid. Yield: 53.6%. m.p. 72–74 °C. ^1^H NMR (400 MHz, CDCl_3_) δ 3.55 (dd, 1H, *J* = 17.2, 4.4 Hz), 3.89–4.04 (m, 10H), 6.15 (dd, 1H, *J* = 11.2, 4.4 Hz), 6.81 (d, 1H, *J* = 8.4 Hz), 6.92–6.96 (m, 2H), 7.16–7.20 (m, 2H), 7.51–7.59 (m, 3H), 7.73 (d, 1H, *J* = 1.6 Hz), 7.84 (dd, 1H, *J* = 8.4, 2.0 Hz), 8.33–8.38 (m, 1H), 9.27–9.32 (m, 1H). ^13^C NMR (100 MHz, CDCl_3_) δ 43.6, 55.4, 55.8, 56.1, 102.9, 109.9, 113.3, 120.3, 122.6, 124.3, 124.7, 125.1, 125.9, 126.2, 126.8 (d, *J* = 21.0 Hz), 128.3, 129.9, 131.7, 144.7, 148.1, 151.5, 155.0, 157.7, 165.9. HRMS (ESI-TOF) *m*/*z*: [M + H]^+^ Calcd. for C_27_H_25_N_2_O_4_S 473.1530, Found 473.1538.

#### 3.2.19. (3-(6-((λ1-Oxidanyl)-λ^5^-methyl) naphthalen-2-yl)-5-(thiophen-2-yl)-4,5-dihydro-1*H*-pyrazol-1-yl)(3,4-dimethoxyphenyl)methanone (**3p**)

Yellow solid. Yield: 52.7%. m.p. 74–76 °C. ^1^H NMR (400 MHz, CDCl_3_) δ 3.49 (dd, 1H, *J* = 17.6, 4.8 Hz), 3.84 (dd, 1H, *J* = 17.6, 11.6 Hz), 3.95 (t, 9H, *J* = 4.8 Hz), 6.18 (dd, 1H, *J* = 11.2, 4.4 Hz), 6.94–6.96 (m, 2H), 7.13–7.21 (m, 4H), 7.73–7.77 (m, 3H), 7.86–7.91 (m, 2H), 7.99 (dd, 1H, *J* = 8.8, 1.2 Hz). ^13^C NMR (100 MHz, CDCl_3_) δ 41.1, 55.5, 56.1, 57.0, 106.2, 109.9, 113.6, 119.7, 124.0, 124.6 (d, *J* = 22.0 Hz), 125.0, 126.4, 126.9 (d, *J* = 10.0 Hz), 127.2, 127.5, 128.4, 130.1, 135.7, 144.6, 148.1, 151.6, 154.8, 159.0, 165.6. HRMS (ESI-TOF) *m*/*z*: [M + H]^+^ Calcd. for C_27_H_25_N_2_O_4_S 473.1530, Found 473.1524.

#### 3.2.20. (3,4-Dimethoxyphenyl)(3-(3,4-dimethoxyphenyl)-5-(thiophen-2-yl)-4,5-dihydro-1*H*-pyrazol-1-yl)methanone (**3q**)

Light yellow solid. Yield: 49.6%. m.p. 127–128 °C. ^1^H NMR (400 MHz, CDCl_3_) δ 3.32 (dd, 1H, *J* = 17.6, 4.4 Hz), 3.70 (dd, 1H, *J* = 17.6, 11.6 Hz), 3.90 (d, 12H, *J* = 8.4 Hz), 6.10 (dd, 1H, *J* = 11.2, 4.4 Hz), 6.85–6.92 (m, 3H), 7.08 (d, 1H, *J* = 3.2 Hz), 7.16–7.18 (m, 2H), 7.37 (d, 1H, *J* = 1.2 Hz), 7.72 (s, 1H), 7.81 (dd, 1H, *J* = 8.4, 1.6 Hz). ^13^C NMR (100 MHz, CDCl_3_) δ 40.9, 55.7–55.8 (m), 56.7, 108.5, 109.6, 110.6, 113.4, 120.5, 124.0, 124.2, 124.4, 124.6, 126.2, 126.6, 144.4, 147.7, 149.0, 151.2 (d, *J* = 19.0 Hz), 154.3, 165.1. HRMS (ESI-TOF) *m*/*z*: [M + H]^+^ Calcd. for C_24_H_25_N_2_O_5_S 453.1479, Found 453.1476.

#### 3.2.21. (3,4-Dimethoxyphenyl)(3-(3,4-dimethylphenyl)-5-(thiophen-2-yl)-4,5-dihydro-1*H*-pyrazol-1-yl)methanone (**3r**)

Light yellow solid. Yield: 46.3%. m.p. 104–106 °C. ^1^H NMR (400 MHz, CDCl_3_) δ 3.30 (d, 6H, *J* = 1.2 Hz), 3.36 (dd, 1H, *J* = 17.6, 4.4 Hz), 3.73 (dd, 1H, *J* = 17.6, 11.6 Hz), 3.94 (d, 6H, *J* = 3.6 Hz), 6.13 (dd, 1H, *J* = 11.2, 4.4 Hz), 6.92–6.94 (m, 2H), 7.10 (d, 1H, *J* = 3.2 Hz), 7.18 (d, 2H, *J* = 6.8 Hz), 7.47 (d, 1H, *J* = 7.6 Hz), 7.53 (s, 1H), 7.73 (d, 1H, *J* = 1.6 Hz), 7.84 (dd, 1H, *J* = 8.4, 1.6 Hz). ^13^C NMR (100 MHz, CDCl_3_) δ 19.9 (d, *J* = 4.0 Hz), 41.2, 56.0 (d, *J* = 4.0 Hz), 56.8, 109.9, 113.6, 124.4, 124.5, 124.7, 124.9, 126.4, 126.9, 127.9, 129.0, 130.1, 137.2, 139.7, 144.7, 148.1, 151.5, 154.8, 165.5. HRMS (ESI-TOF) *m*/*z*: [M + H]^+^ Calcd. for C_24_H_25_N_2_O_3_S 421.1580, Found 421.1583.

#### 3.2.22. (3-(Benzo[d][1,3]dioxol-5-yl)-5-(thiophen-2-yl)-4,5-dihydro-1*H*-pyrazol-1-yl)(3,4-dimethoxyphenyl)methanone (**3s**)

Light yellow solid. Yield: 63.6%. m.p. 138–140 °C. ^1^H NMR (400 MHz, CDCl_3_) δ 3.23 (dd, 1H, *J* = 17.2, 4.4 Hz), 3.61 (dd, 1H, *J* = 17.2, 11.2 Hz), 3.86 (d, 6H, *J* = 6.4 Hz), 5.93 (s, 2H), 6.04 (dd, 1H, *J* = 11.2, 4.4 Hz), 6.74–6.77 (m, 1H), 6.83–6.87 (m, 2H), 7.02–7.05 (m, 2H), 7.11 (d, 1H, *J* = 4.8 Hz), 7.19–7.26 (m, 1H), 7.59 (d, 1H, *J* = 1.2 Hz), 7.74 (dd, 1H, *J* = 8.4, 1.2 Hz). ^13^C NMR (100 MHz, CDCl_3_) δ 40.2, 55.0, 55.9, 100.7, 105.3, 107.4, 108.8, 112.5, 120.9, 123.5, 123.7, 124.0, 124.7, 125.3, 125.9, 143.5, 147.1 (d, *J* = 1.0 Hz), 147.3, 148.8 (d, *J* = 1.0 Hz), 150.5, 153.2, 164.5. HRMS (ESI-TOF) *m*/*z*: [M + H]^+^ Calcd. for C_23_H_21_N_2_O_5_S 437.1166, Found 437.1163.

#### 3.2.23. (3-(5-Bromopyridin-2-yl)-5-(thiophen-2-yl)-4,5-dihydro-1*H*-pyrazol-1-yl)(3,4-dimethoxyphenyl)methanone (**3t**)

White solid. Yield: 58.7%. m.p. 77–79 °C. ^1^H NMR (400 MHz, CDCl_3_) δ 3.55 (dd, 1H, *J* = 18.4, 4.8 Hz), 3.77–3.92 (m, 7H), 6.13 (dd, 1H, *J* = 11.6, 4.8 Hz), 6.88–6.93 (m, 2H), 7.10 (d, 1H, *J* = 3.2 Hz), 7.17 (d, 1H, *J* = 5.2 Hz), 7.58 (d, 1H, *J* = 0.8 Hz), 7.74 (dd, 1H, *J* = 8.4, 1.2 Hz), 7.81 (dd, 1H, *J* = 8.8, 4.0 Hz), 7.89 (d, 1H, *J* = 8.4 Hz), 8.64 (d, 1H, *J* = 1.2 Hz). ^13^C NMR (100 MHz, CDCl_3_) δ 40.8, 56.1 (d, *J* = 1.0 Hz), 57.4, 109.9, 113.5, 121.9, 122.4, 124.4, 124.9, 125.3, 126.2, 127.0, 139.1, 144.2, 148.3, 149.3, 150.7, 151.8, 155.2, 166.2. HRMS (ESI-TOF) *m*/*z*: [M + H]^+^ Calcd. for C_21_H_19_BrN_3_O_3_S 472.0325, Found 472.0329.

### 3.3. Biological Assay

#### 3.3.1. The PI3K Inhibition Assay

The competitive fluorescence polarization kinase activity assay was used to test the inhibition potency on PI3K, as mentioned in previous investigations [31,32]. In general, following the instruction of the Kit (Perkin–Elmer, Waltham, MA, USA), the 10 μL scale of PI3K activity reaction was conducted with the substrate diC_8_-PI(4,5)*P*_2_ in a solution containing 2.5 mM MgCl_2_, 5 mM HEPES, 50 mM ATP and 10 mM DTT at pH 7.0. Into the 10 μL reaction wells with 50 ng enzyme and 10 mM substrate, different concentrations of the tested compounds were added, respectively. The mixture was incubated at room temperature for 3 h, and the reactions were then quenched. Afterwards, the phosphoinositide binding protein and the fluorescent dye-labeled phosphoinositide tracer were added in sequence. In the dark environment in 384-well black nonbinding plates (Corning, Corning, NY, USA), the further incubation lasted for 1 h. With appropriate filters, the polarization values of the corresponding dye were recorded to calculate the enzymatic activity of PI3K in this reacting procedure. This assay was universal for evaluating the activity of different PI3K isoforms.

#### 3.3.2. Anti-Proliferation Assay

In this section, the cells included HeLa (human epithelial cervical cancer cell line), HepG2 (human hepatocellular cancer cell line) and L02 (human normal hepatocyte line) to evaluate the anti-proliferation activity of the synthesized compounds. The cells were cultured in Dulbecco’s modified eagle medium (DMEM, Hyclone) with the addition of 10% fetal bovine serum (FBS, BI), 2 mmol/L L-glutamine, 100 units/mL penicillin-streptomycin (Sigma-Aldrich, Saint Louis, MO, USA), 100 mg/mL streptomycin (Hyclone, Logan, UT, USA). The environment was 5% CO_2_ contained atmosphere at 37 °C. The tests of anti-proliferative activity were conducted under a standard method with the typical dye 3-(4,5-dimethylthiazol-2-yl)-2,5-diphenyltetrazolium bromide (MTT) in triplicate [39,40]. The LX300 Epson Diagnostic micro-plate reader (Nagano, Japan) was used to record the absorbance at 570 nm (OD_570_).

#### 3.3.3. Western Blot

According to the results of the anti-proliferation assay, HepG2 cells were chosen to be incubated with the compound **3s** for 24 h. The cells were then trypsinized and collected. Afterwards, the extracting of the total proteins was conducted with a 1 × RIPA lysis buffer (1% NP-40, 50 mM Tris-HCl, 150 mM NaCl, 0.25% deoxycholic acid, 1 mM EDTA containing protease inhibitors PMSF, pH 7.4,) (Amresco, Solon, OH, USA). Then, the protein extract was separated by 10% sodium dodecyl sulfate polyacrylamide gel electrophoresis (SDS-PAGE), transferred onto the PVDF membrane, blotted with primary antibodies, and marked with secondary isotype-specific antibodies tagging horseradish peroxidase. Finally, the immunocomplexes were pictured under the ChemiDOC™ XRS + system (BioRad Laboratories, Hercules, CA, USA).

### 3.4. Protocol of Docking Simulation

The depiction of the compounds was performed in Chemdraw 14.0 software (Cambridge Soft corporation, Cambridge, MA, USA (2012)). The synthesized compounds were defined as ligands, while the proteins were defined as the receptors. The high-resolution complexes of PI3Kα (PDB Code: 1E7V) and PI3Kγ (PDB Code: 3APF) were downloaded from RSCB Protein Data Bank (http://www.rcsb.org, accessed on 20 November 2021). Referring to the previous reports, all the proteins and ligands were prepared by minimization under the CHARMM force field [41,42]. Then, the molecular docking simulation was conducted using AutoDock 4.2 (The Scripps Research Institute) software [43]. The results were visualized by Discovery Studio Visualizer 2016 (BIOVIA, San Diego, CA, USA).

## 4. Conclusions

In summary, based on the candidates in clinical trials, a series of thiophene-containing triaryl pyrazoline derivatives, **3a**–**3t**, were synthesized and evaluated regarding PI3K inhibition activity and anti-tumor potency. In addition to introducing the significant moieties, including pyrazoline and thiophene, we simplified the parallel ring structures to eliminate the colorimetric interference in previous reports. The majority of the tested compounds indicated potent PI3K inhibitory potency. The results inferred that this series of compounds were more potent for PI3Kγ than PI3Kα. The top hit **3s** seemed more potent than the positive control **LY294002** on inhibiting PI3Kγ (IC_50_ values: 0.066 μM versus 0.777 μM) and more selective from PI3Kα (Index values: 645 versus 1.74). The preliminary SAR discussion indicated that the combination of *para*- and *meta*-, as well as the modification of the electron-donating moieties, led to an improvement in potency. Although both pan blockers and specific inhibitors were essential, the selectivity towards PI3Kγ in this work was meaningful. The anti-proliferation inhibitory activity and the enzymatic inhibition potency indicated the consistent tendencies; thus, the cellular inhibition might be caused by the effect on PI3K. In comparison with different cell lines, this series showed better potency on HepG2 than HeLa. This result agreed with the fact that the PI3K-associated metabolism was more frequent in liver than in model cancer cells. The top hit could inhibit the phosphorylation of Akt by inhibiting PI3K through the PI3K-Akt-mTOR pathway. In the molecular docking simulation, compared with the binding pattern into PI3Kα, it was indicated that more hydrogen bond and much more π-involved interactions were introduced to PI3Kγ, while the π-sulfur interactions were not involved. Both the thiophene moiety and the aryl group might participate in the interaction of hydrogen bonds. These results agreed with the selectivity for PI3Kγ from PI3Kα. The information in this work is referable for the further development of selective inhibitors for specific isoforms of PI3K.

## Figures and Tables

**Table 1:** The PI3K inhibition, cytotoxicity, and anti-proliferation activity of the synthesized compounds **3a-3t**.

Code	IC_50_ (μM)		CC_50_ (μM)	GI_50_ (μM)	
	PI3Kα	PI3Kγ	L02	HeLa	HepG2γ
**3a**	>100.000	7.480 ± 0.220	174.000 ± 10.000	109.000 ± 5.800	61.100 ± 3.200
**3b**	>100.000	4.430 ± 0.200	152.000 ± 9.500	77.900 ± 3.900	50.200 ± 1.600
**3c**	30.900 ± 1.300	4.210 ± 0.072	94.500 ± 4.300	26.300 ± 1.700	20.400 ± 2.000
**3d**	78.700 ± 4.000	2.220 ± 0.072	120.000 ± 4.700	57.100 ± 2.600	42.600 ± 1.900
**3e**	2.080 ± 0.099	1.120 ± 0.081	58.400 ± 2.600	5.900 ± 0.200	5.510 ± 0.200
**3f**	26.600 ± 1.010	0.570 ± 0.044	112.000 ± 8.300	23.700 ± 1.200	15.100 ± 0.420
**3g**	0.920 ± 0.056	26.900 ± 1.400	103.500 ± 5.400	31.600 ± 0.900	31.600 ± 1.400
**3h**	64.400 ± 3.600	18.100 ± 1.700	180.000 ± 4.600	61.300 ± 2.300	53.300 ± 1.900
**3i**	89.500 ± 3.400	3.030 ± 0.085	151.000 ± 8.900	66.600 ± 1.200	48.700 ± 1.800
**3j**	3.920 ± 0.064	>100.000	196.000 ± 10.000	75.700 ± 2.900	50.600 ± 1.000
**3k**	1.100 ± 0.061	42.700 ± 2.600	178.000 ± 8.700	44.600 ± 2.100	46.600 ± 1.300
**3l**	17.100 ± 0.580	5.110 ± 0.083	86.600 ± 3.500	20.100 ± 1.700	15.500 ± 0.870
**3m**	75.600 ± 2.400	2.520 ± 0.055	141.000 ± 5.600	57.900 ± 3.600	40.100 ± 1.400
**3n**	12.000 ± 1.100	1.790 ± 0.049	62.600 ± 2.600	11.100 ± 1.200	8.850 ± 0.310
**3o**	8.810 ± 0.170	9.910 ± 0.260	137.000 ± 4.700	18.100 ± 0.850	16.900 ± 0.360
**3p**	0.560 ± 0.045	3.760 ± 0.190	67.100 ± 3.900	6.810 ± 0.240	6.310 ± 0.540
**3q**	5.240 ± 0.130	0.430 ± 0.036	41.300 ± 2.600	4.720 ± 0.120	4.220 ± 0.130
**3r**	4.130 ± 0.062	1.370 ± 0.055	52.500 ± 3.200	6.200 ± 0.220	5.550 ± 0.410
**3s**	42.600 ± 2.900	0.066 ± 0.005	181.000 ± 9.000	13.700 ± 1.100	12.900 ± 1.300
**3t**	>100.000	9.150 ± 0.140	>300.000	124.000 ± 9.700	109.700 ± 8.100
**LY294002**	0.447 ± 0.038	0.777 ± 0.071	51.300 ± 1.400	21.100 ± 2.000	26.500 ± 1.900

**Table 2:** The structures of the synthesized compounds **3a**–**3t**.

Code	Structure	Code	Structure
**3a**	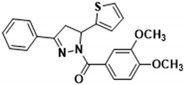	**3k**	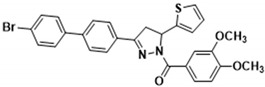
**3b**	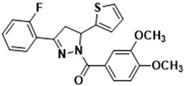	**3l**	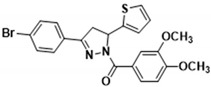
**3c**	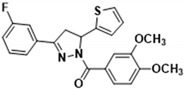	**3m**	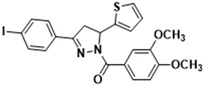
**3d**	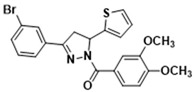	**3n**	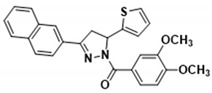
**3e**	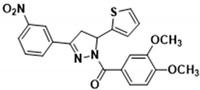	**3o**	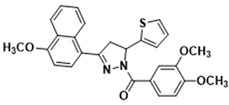
**3f**	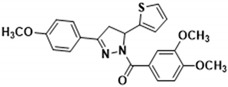	**3p**	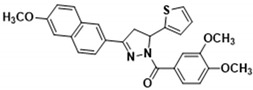
**3g**	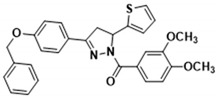	**3q**	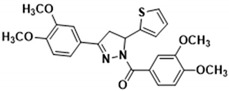
**3h**	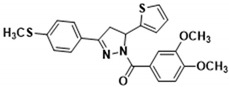	**3r**	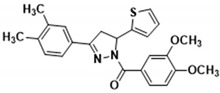
**3i**	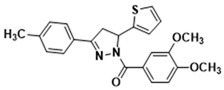	**3s**	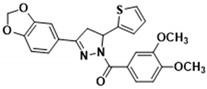
**3j**	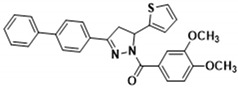	**3t**	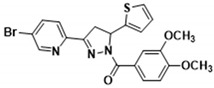

## Data Availability

Not applicable.

## Supplementary Materials

The following Supplementary Materials can be downloaded at: https://www.mdpi.com/article/10.3390/molecules27082404/s1, Figure S1: NMR Spectra (^1^H NMR Spectra and ^13^C NMR Spectra, pp. 1–20); Figure S2: HRMS analytical data (pp. 21–30).

## Sample Availability

Samples of the chalcone compounds are available from the authors.

## References

[1] Singh V.J., Sharma B., Chawla P.A. (2021). Recent developments in mitogen activated protein kinase inhibitors as potential anticancer agents. Bioorg. Chem..

[2] Rascio F., Spadaccino F., Rocchetti M.T., Castellano G., Stallone G., Netti G.S., Ranieri E. (2021). The pathogenic role of PI3K/AKT pathway in cancer onset and drug resistance: An updated review. Cancers.

[3] Kumar V., Vashishta M., Kong L., Wu X., Lu J.J., Guha C., Dwarakanath B.S. (2021). The role of Notch, Hedgehog, and Wnt signaling pathways in the resistance of tumors to anticancer therapies. Front. Cell Dev. Biol..

[4] Meng D., He W., Zhang Y., Liang Z., Zheng J., Zhang X., Zheng X., Zhan P., Chen H., Li W. (2021). Development of PI3K inhibitors: Advances in clinical trials and new strategies (Review). Pharmacol. Res..

[5] Sabbah D.A., Hajjo R., Bardaweel S.K., Zhong H.A. (2021). Phosphatidylinositol 3-kinase (PI3K) inhibitors: A recent update on inhibitor design and clinical trials (2016–2020). Expert Opin. Ther. Pat..

[6] Rathinaswamy M.K., Gaieb Z., Fleming K.D., Borsari C., Harris N.J., Moeller B.E., Wymann M.P., Amaro R.E., Burke J.E. (2021). Disease-related mutations in PI3Ky disrupt regulatory C-terminal dynamics and reveal a path to selective inhibitors. eLife.

[7] Ghafouri-Fard S., Abak A., Shoorei H., Mohaqiq M., Majidpoor J., Sayad A., Taheri M. (2021). Regulatory role of microRNAs on PTEN signaling. Biomed. Pharmacother..

[8] Kim M.J., Kim H., Gao X., Ryu J.H., Yang Y., Kwon I.C., Roberts T.M., Kim S.H. (2021). Multi-targeting siRNA nanoparticles for simultaneous inhibition of PI3K and Rac1 in PTEN-deficient prostate cancer. J. Ind. Eng. Chem..

[9] Calderón L., Schindler K., Malin S.G., Schebesta A., Sun Q., Schwickert T., Alberti C., Fischer M., Jaritz M., Tagoh H. (2021). Pax5 regulates B cell immunity by promoting PI3K signaling via PTEN down-regulation. Sci. Immunol..

[10] Rathinaswamy M.K., Dalwadi U., Fleming K.D., Adams C., Stariha J.T.B., Pardon E., Baek M., Vadas O., Dimaio F., Steyaert J. (2021). Structure of the phosphoinositide 3-kinase (PI3K) p110γ-p101 complex reveals molecular mechanism of GPCR activation. Sci. Adv..

[11] Li X., He S., Ma B. (2020). Autophagy and autophagy-related proteins in cancer. Mol. Cancer.

[12] Sun P., Meng L.-H. (2020). Emerging roles of class I PI3K inhibitors in modulating tumor microenvironment and immunity. Acta Pharmacol. Sin..

[13] Bilanges B., Posor Y., Vanhaesebroeck B. (2019). PI3K isoforms in cell signalling and vesicle traffickin. Nat. Rev. Mol. Cell Biol..

[14] Endicott S.J., Ziemba Z.J., Beckmann L.J., Boynton D.N., Miller R.A. (2020). Inhibition of class I PI3K enhances chaperone-mediated autophagy. J. Cell Biol..

[15] Aksoy E., Saveanu L., Manoury B. (2018). The isoform selective roles of PI3Ks in dendritic cell biology and function. Front. Immunol..

[16] Sadasivan C., Zhabyeyev P., Labib D., White J.A., Paterson D.I., Oudit G.Y. (2020). Cardiovascular toxicity of PI3Kα inhibitors. Clin. Sci..

[17] Pennino F.P., Murakami M., Zollo M., Robertson E.S. (2021). The metastasis suppressor protein NM23-H1 modulates the PI3K-AKT axis through interaction with the p110α catalytic subunit. Oncogenesis.

[18] Marshall J.D.S., Whitecross D.E., Mellor P., Anderson D.H. (2019). Impact of p85α alterations in cancer. Biomolecules.

[19] Luff D.H., Wojdyla K., Oxley D., Chessa T., Hudson K., Hawkins P.T., Stephens L.R., Barry S.T., Okkenhaug K. (2021). PI3Kδ forms distinct multiprotein complexes at the TCR signalosome in naïve and differentiated CD4^+^ T Cells. Front. Immunol..

[20] Fujita A., Kan-o K., Tonai K., Yamamoto N., Ogawa T., Fukuyama S., Nakanishi Y., Matsumoto K. (2020). Inhibition of PI3Kδ enhances poly I: C-induced antiviral responses and inhibits replication of human metapneumovirus in murine lungs and human bronchial epithelial cells. Front. Immunol..

[21] de Assis Lima I.V., Bellozi P.M.Q., Batista E.M., Vilela L.R., Brandão I.L., Ribeiro F.M., Moraes M.F.D., Moreira F.A., de Oliveira A.C.P. (2020). Cannabidiol anticonvulsant effect is mediated by the PI3Kγ pathway. Neuropharmacology.

[22] Perry M.W.D., Abdulai R., Mogemark M., Petersen J., Thomas M.J., Valastro B., Eriksson A.W. (2019). Evolution of PI3Kγ and δ inhibitors for inflammatory and qutoimmune diseases. J. Med. Chem..

[23] Vanhaesebroeck B., Perry M.W.D., Brown J.R., André F., Okkenhaug K. (2021). PI3K inhibitors are finally coming of age. Nat. Rev. Drug Discov..

[24] Rodon J., Dienstmann R., Serra V., Tabernero J. (2013). Development of PI3K inhibitors: Lessons learned from early clinical trials. Nat. Rev. Clin. Oncol..

[25] Ghareghomi S., Ahmadian S., Zarghami N., Hemmati S. (2021). hTERT-molecular targeted therapy of ovarian cancer cells via folate-functionalized PLGA nanoparticles co-loaded with MNPs/siRNA/wortmannin. Life Sci..

[26] Wang J., Lv X., Guo X., Dong Y., Peng P., Huang F., Wang P., Zhang H., Zhou J., Wang Y. (2021). Feedback activation of STAT3 limits the response to PI3K/AKT/mTOR inhibitors in PTEN-deficient cancer cells. Oncogenesis.

[27] Down K., Amour A., Baldwin I.R., Cooper A.W.J., Deakin A.M., Felton L.M., Guntrip S.B., Hardy C., Harrison Z.A., Jones K.L. (2015). Optimization of novel indazoles as highly potent and selective inhibitors of phosphoinositide 3-kinase δ for the treatment of respiratory disease. J. Med. Chem..

[28] Wallin J.J., Guan J., Prior W.W., Lee L.B., Berry L., Belmont L.D., Koeppen H., Belvin M., Friedman L.S., Sampath D. (2012). GDC-0941, a novel class I selective PI3K inhibitor, enhances the efficacy of docetaxel in human breast cancer models by increasing cell death in vitro and in vivo. Clin. Cancer Res..

[29] Omeljaniuk W.J., Krętowski R., Ratajczak-Wrona W., Jablońska E., Cechowska-Pasko M. (2021). Novel dual PI3K/mTOR inhibitor, Apitolisib (GDC-0980), inhibits growth and induces apoptosis in human glioblastoma cells. Int. J. Mol. Sci..

[30] Lauder S.N., Smart K., Kersemans V., Allen D., Scott J., Pires A., Milutinovic S., Somerville M., Smart S., Kinchesh P. (2020). Enhanced antitumor immunity through sequential targeting of PI3Kδ and LAG3. J. Immunother. Cancer.

[31] Yin Y., Sha S., Wu X., Wang S.-F., Qiao F., Song Z.-C., Zhu H.-L. (2019). Development of novel chromeno[4,3-*c*]pyrazol-4(2*H*)-one derivates bearing sulfonylpiperazine as antitumor inhibitors targeting PI3Kα. Eur. J. Med. Chem..

[32] Yin Y., Zhou Y., Sha S., Wu X., Wang S.-F., Qiao F., Song Z.-C., Zhu H.-L. (2019). Development of novel chromeno[4,3-*c*]pyrazol-4(2*H*)-one derivates containing piperazine as inhibitors of PI3Kα. Bioorg. Chem..

[33] Tang J.-F., Lv X.-H., Wang X.-L., Sun J., Zhang Y.-B., Yang Y.-S., Gong H.-B., Zhu H.-L. (2012). Design, synthesis, biological evaluation and molecular modeling of novel 1,3,4-oxadiazole derivatives based on Vanillic acid as potential immunosuppressive agents. Bioorg. Med. Chem..

[34] Elewa S.I., Mansour E., Nassar I.F., Mekawey A.A.I. (2020). Synthesis of some new pyrazoline-based thiazole derivatives and evaluation of their antimicrobial, antifungal, and anticancer activities. Russ. J. Bioorg. Chem..

[35] Yang B., Zhou J., Wang F., Hu X.-W., Shi Y. (2021). Pyrazoline derivatives as tubulin polymerization inhibitors with one hit for Vascular Endothelial Growth Factor Receptor 2 inhibition. Bioorg. Chem..

[36] Alex J.M., Singh S., Kumar R. (2014). 1-Acetyl-3,5-diaryl-4,5-dihydro(1*H*)pyrazoles: Exhibiting anticancer activity through intracellular ROS scavenging and the Mitochondria-dependent death pathway. Arch. Pharm..

[37] Qin Y.-J., Li Y.-J., Jiang A.-Q., Yang M.-R., Zhu Q.-Z., Dong H., Zhu H.-L. (2015). Design, synthesis and biological evaluation of novel pyrazoline-containing derivatives as potential tubulin assembling inhibitor. Eur. J. Med. Chem..

[38] Chen K., Zhang Y.-L., Fan J., Ma X., Qin Y.-J., Zhu H.-L. (2018). Novel nicotinoyl pyrazoline derivates bearing N-methyl indole moiety as antitumor agents: Design, synthesis and evaluation. Eur. J. Med. Chem..

[39] Stockert J.C., Horobin R.W., Colombo L.L., Blazquez-Castro A. (2018). Tetrazolium salts and formazan products in Cell Biology: Viability assessment, fluorescence imaging, and labeling perspectives. Acta Histochem..

[40] Xie J.-H., Liu X., Shen M.-Y., Nie S.-P., Zhang H., Li C., Gong D.-M., Xie M.-Y. (2013). Purification, physicochemical characterisation and anticancer activity of a polysaccharide from Cyclocarya paliurus leaves. Food Chem..

[41] Xu Y.-J., Su M.-M., Li H.-L., Liu Q.-X., Xu C., Yang Y.-S., Zhu H.-L. (2018). A fluorescent sensor for discrimination of HSA from BSA through selectivity evolution. Anal. Chim. Acta.

[42] Lin H.-Y., Han H.-W., Sun W.-X., Yang Y.-S., Tang C.-Y., Lu G.-H., Qi J.-L., Wang X.-M., Yang Y.-H. (2018). Design and characterization of α-lipoic acyl shikonin ester twin drugs as tubulin and PDK1 dual inhibitors. Eur. J. Med. Chem..

[43] Morris G.M., Huey R., Lindstrom W., Sanner M.F., Belew R.K., Goodsell D.S., Olson A.J. (2009). Autodock4 and AutoDockTools4: Automated docking with selective receptor flexibility. J. Comput. Chem..

