# Supplementary material for: Development of New Thiophene-Containing Triaryl Pyrazoline Derivatives as PI3Kγ Inhibitors
Source: Molecules. 2022 Apr 8;27(8):2404. doi: 10.3390/molecules27082404 (PMC9027920; doi:10.3390/molecules27082404)
Supplement: Supplementary file 1 [file molecules-27-02404-s001.zip › molecules-1671653-supplementary.pdf]

Supporting Information

Development of New Thiophene-containing Triaryl Pyrazoline Derivatives as PI3Kγ Inhibitors

Bing Yang [1,‡,*], Bo Zhang [2,‡], Qun Zhao [1], Jin Li [3,*], and Yujun Shi [1,*]

[1] School of Chemistry and Chemical Engineering, Nantong University, Nantong 226019, China;
[2] State Key Laboratory of Pharmaceutical Biotechnology, Nanjing University, Nanjing 210023, China;
[3] National and Local Joint Engineering Research Center for Mineral Salt Deep Utilizatio; Key Laboratory for Palygorskite Science and Applied Technology of Jiangsu Province; Huaiyin Institute of Technology, Huai'an 223003, China.
* Correspondence: yangbing111@ntu.edu.cn (B.Y.); lijin96998@126.com (J. L.); syj@ntu.edu.cn (Y.S.).
‡ The authors contributed equally to this work.

Contents

NMR Spectra (^{1}H NMR Spectra and ^{13}C NMR Spectra) 1

HRMS analytical data 21

NMR Spectra (^{1}H NMR Spectra and ^{13}C NMR Spectra)

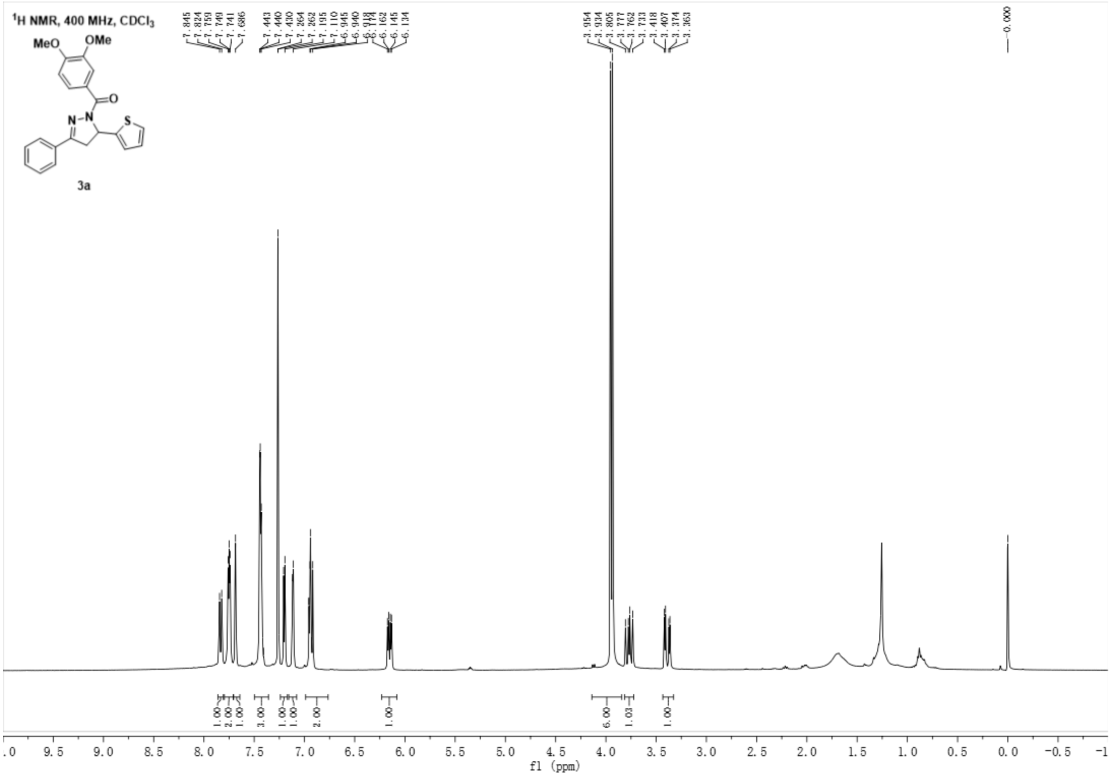

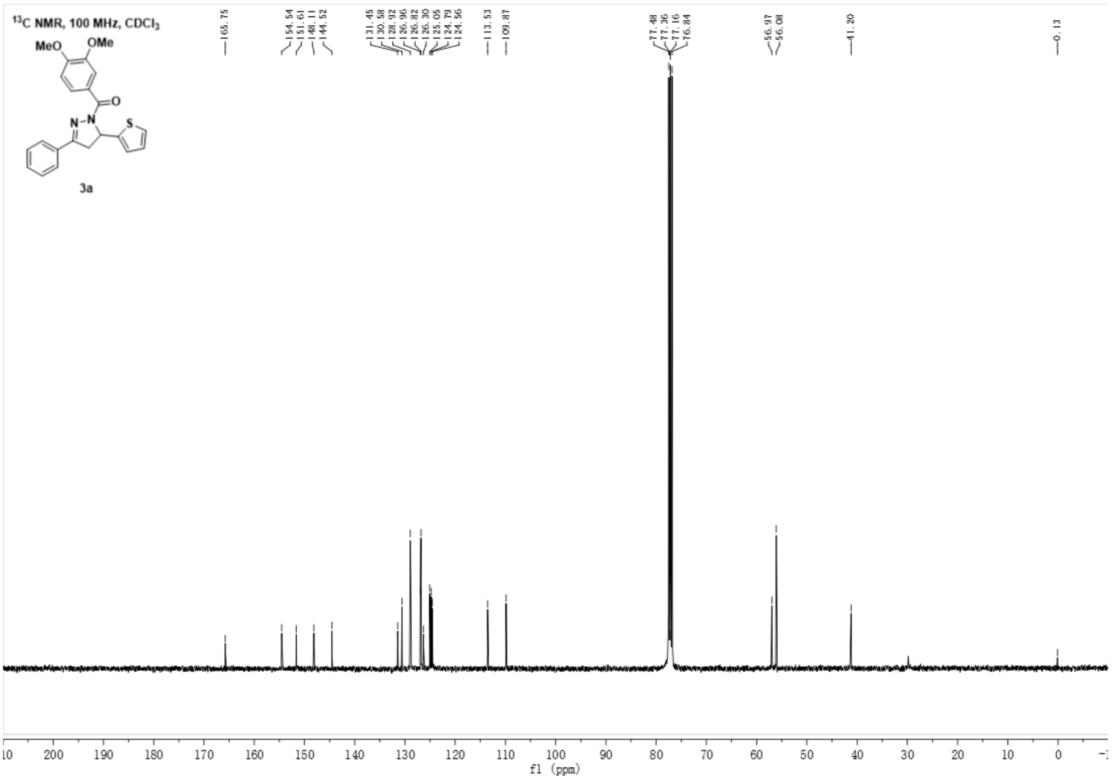

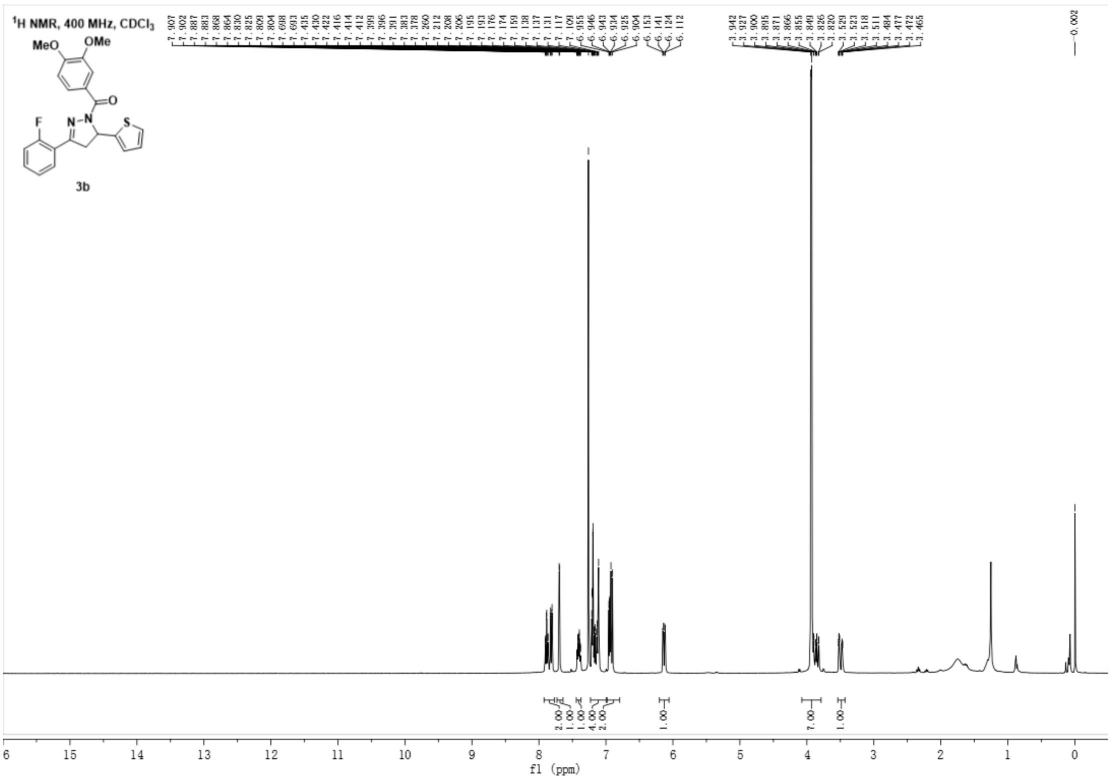
1H NMR, 400 MHz, CDCl3
MeO
OMe
O
N
N
F
S
3b
f1 (ppm)

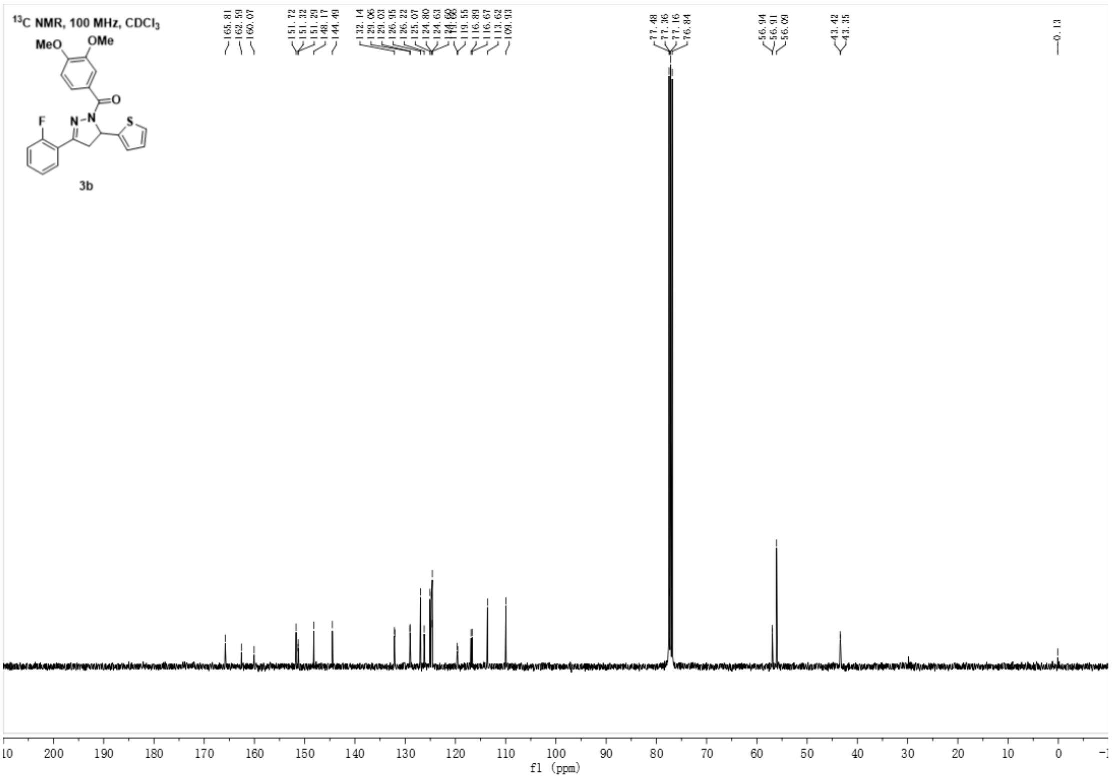
13C NMR, 100 MHz, CDCl3
MeO
OMe
O
N
N
F
S
3b
f1 (ppm)

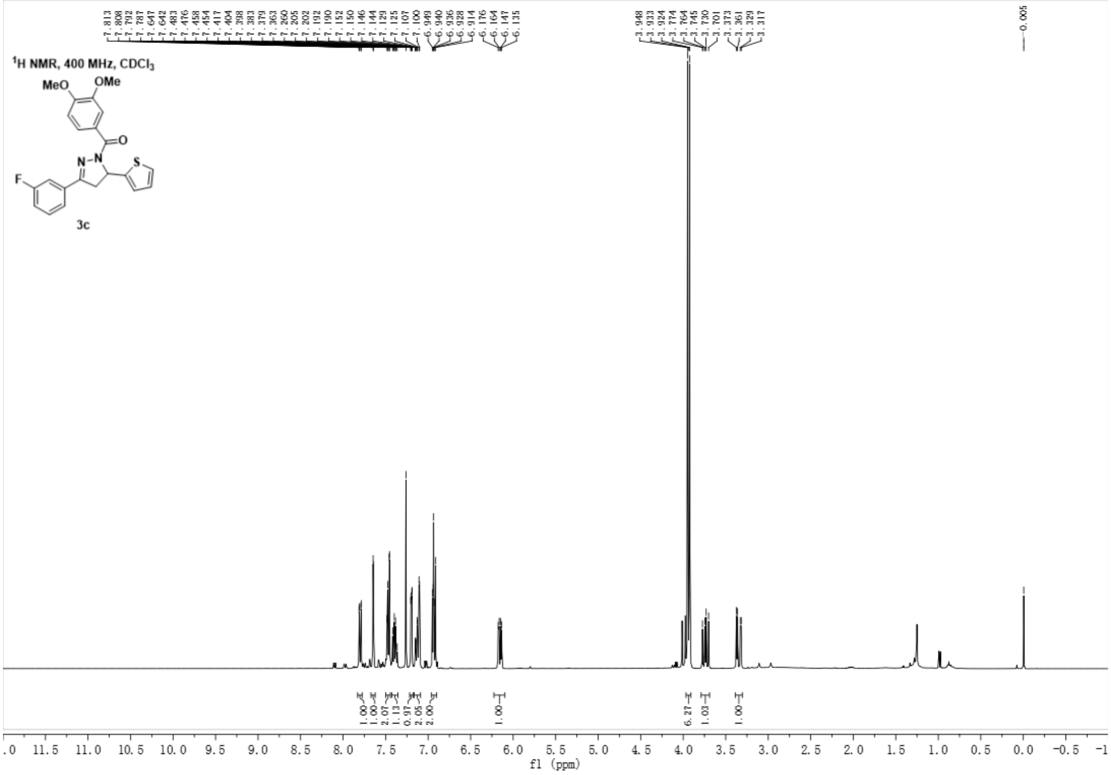
1H NMR, 400 MHz, CDCl3
MeO
OMe
3c
f1 (ppm)

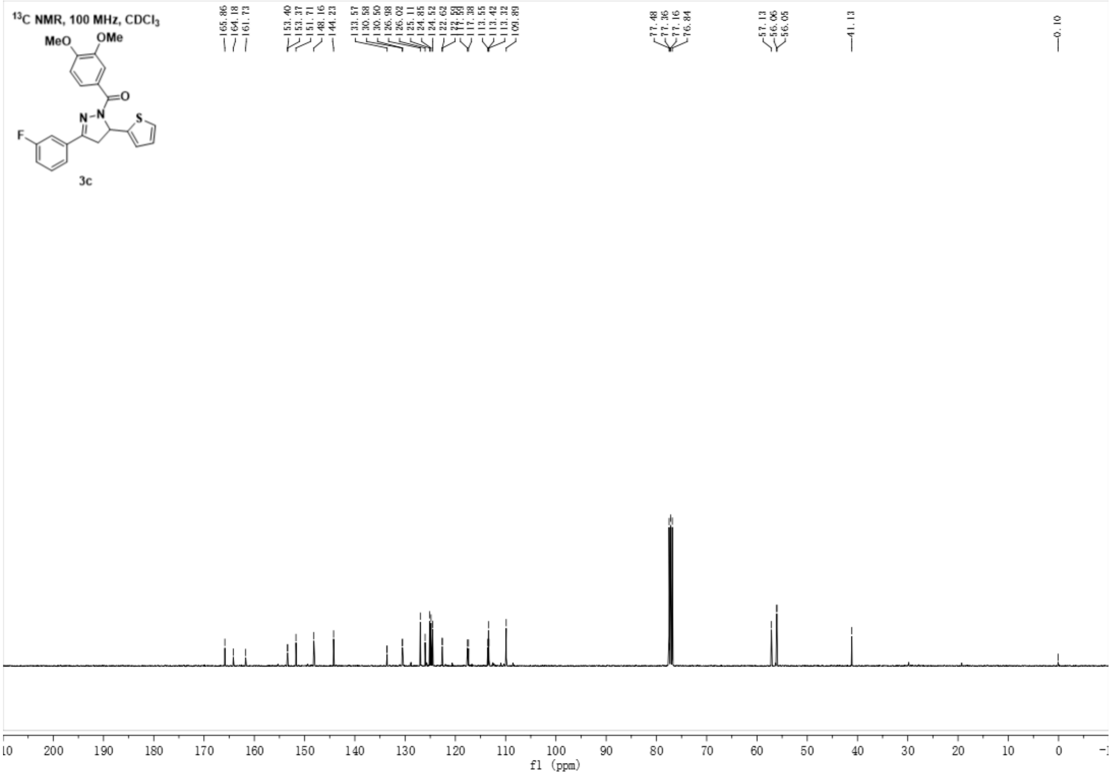
13C NMR, 100 MHz, CDCl3
MeO
OMe
3c
f1 (ppm)

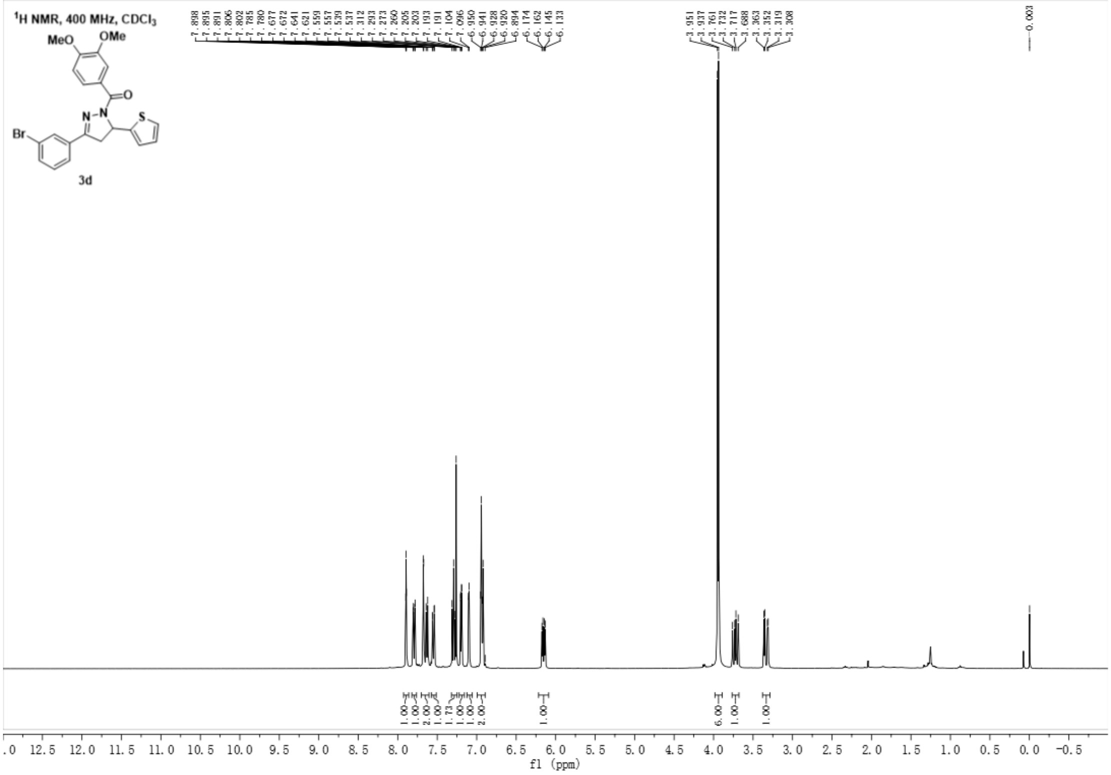
¹H NMR, 400 MHz, CDCl3
MeO
OMe
O
N
N
S
Br
3d
f1 (ppm)

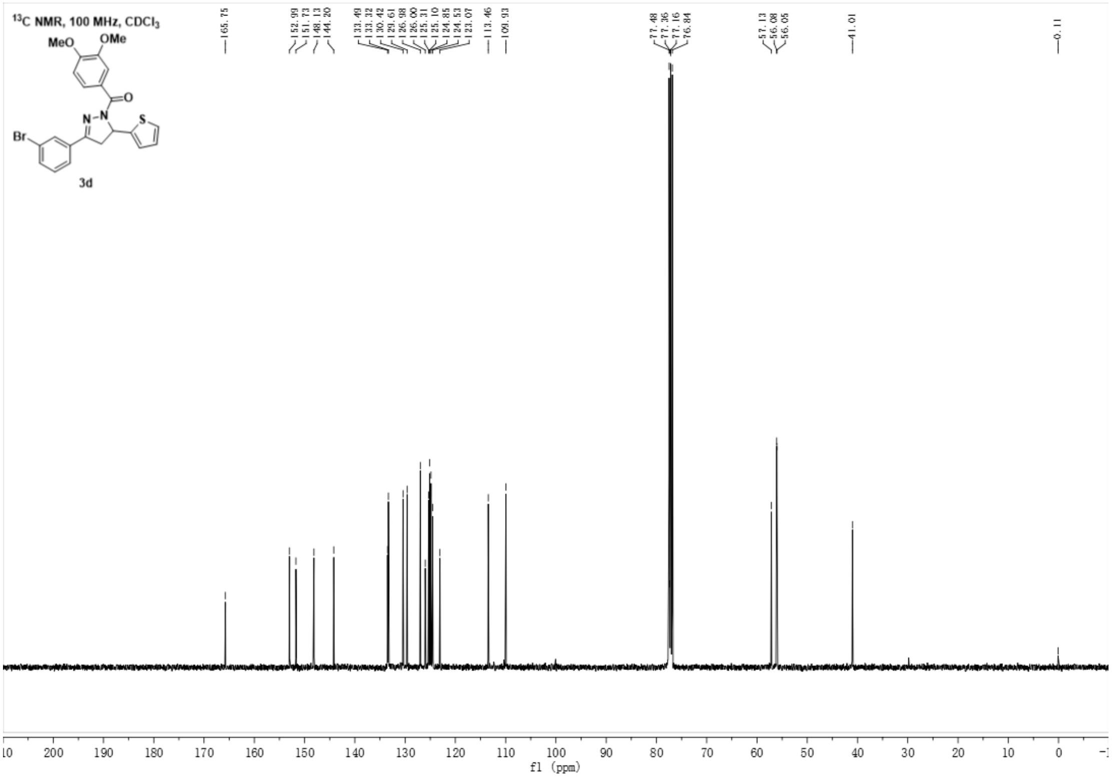
¹³C NMR, 100 MHz, CDCl3
MeO
OMe
O
N
N
S
Br
3d
f1 (ppm)

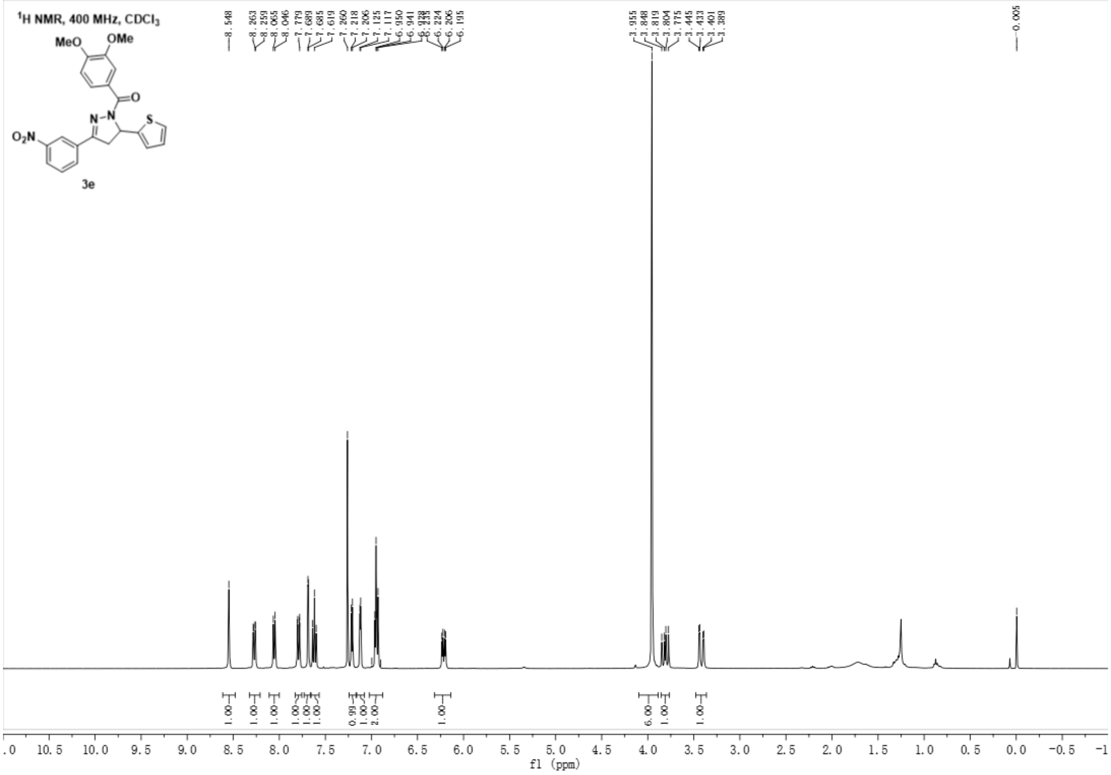
1H NMR, 400 MHz, CDCl3
MeO
OMe
O2N
3e
f1 (ppm)

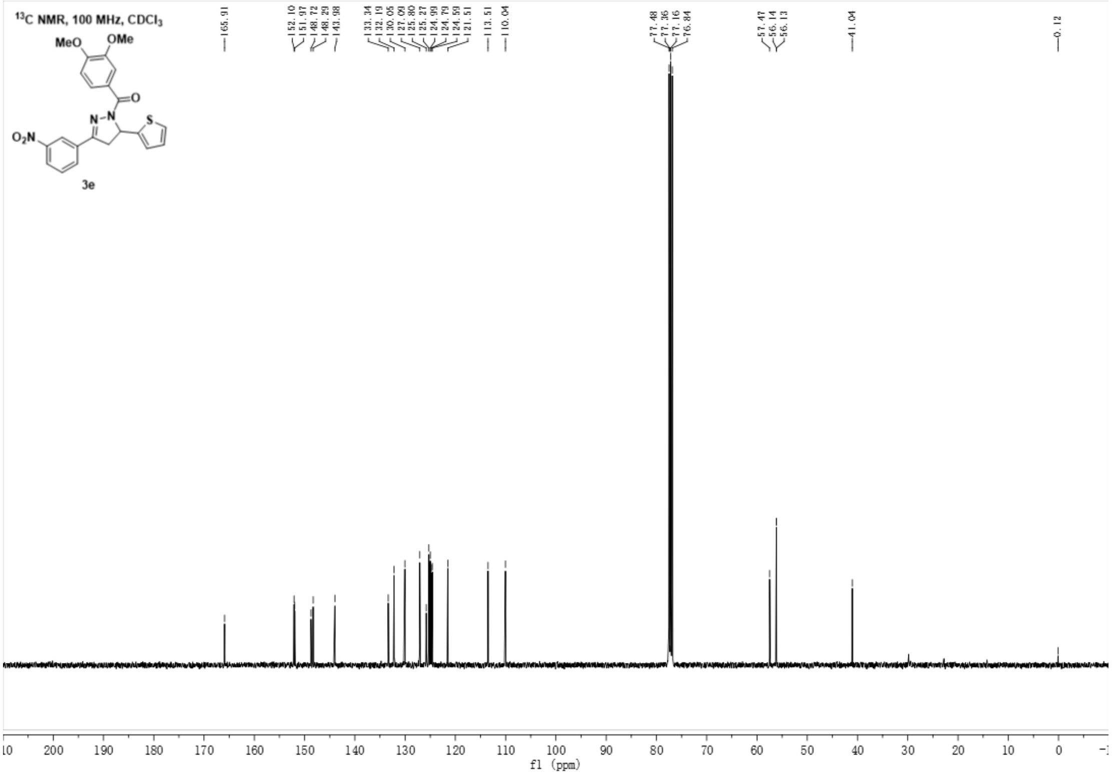
13C NMR, 100 MHz, CDCl3
MeO
OMe
O2N
3e
f1 (ppm)

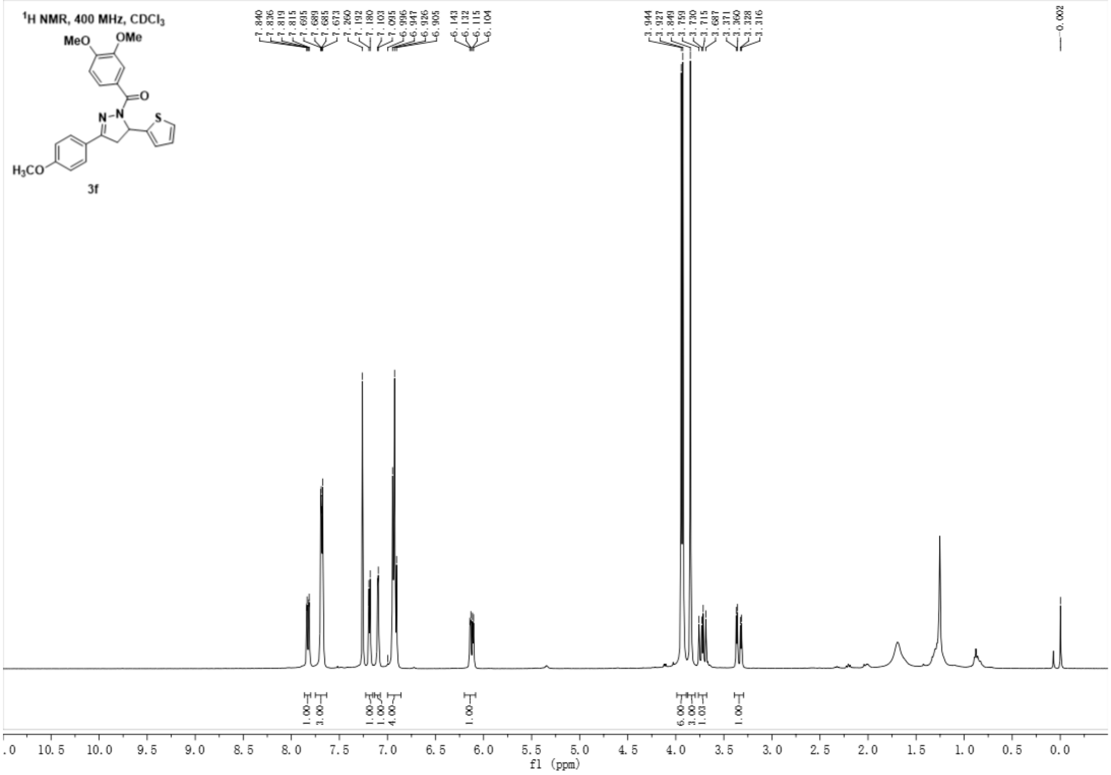
¹H NMR, 400 MHz, CDCl3
MeO
OMe
O
N
N
S
H3CO
3f
7.840 7.836 7.819 7.815 7.695 7.689 7.685 7.673 7.260 7.192 7.180 7.103 7.095 6.996 6.947 6.926 6.905
6.143 6.132 6.115 6.104
3.944 3.927 3.849 3.759 3.730 3.715 3.687 3.371 3.360 3.328 3.316
0.002
1.00 3.00 1.00 1.00 4.00 1.00 6.00 3.00 1.03 1.00
f1 (ppm)

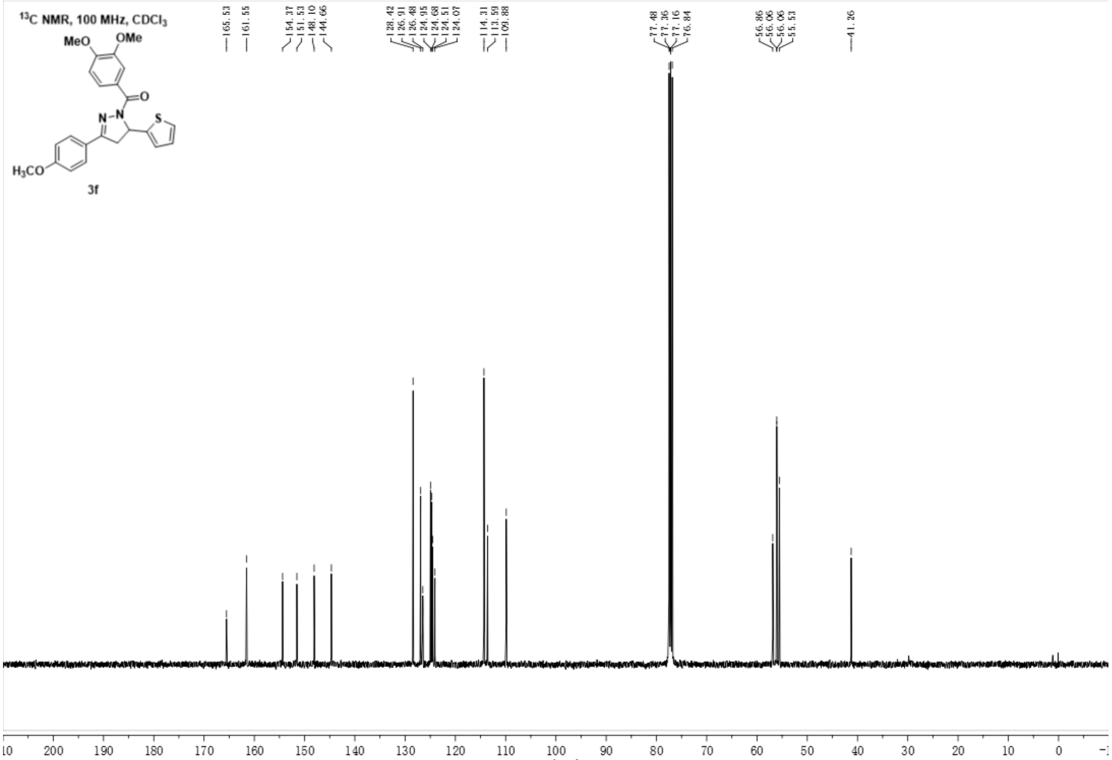
¹³C NMR, 100 MHz, CDCl3
MeO
OMe
O
N
N
S
H3CO
3f
165.53 161.55 154.37 151.53 148.10 144.06
128.42 126.91 126.48 124.95 124.68 124.51 124.07 114.31 113.59 109.88
77.48 77.36 77.16 76.84
56.86 56.06 56.06 55.53
41.26
f1 (ppm)

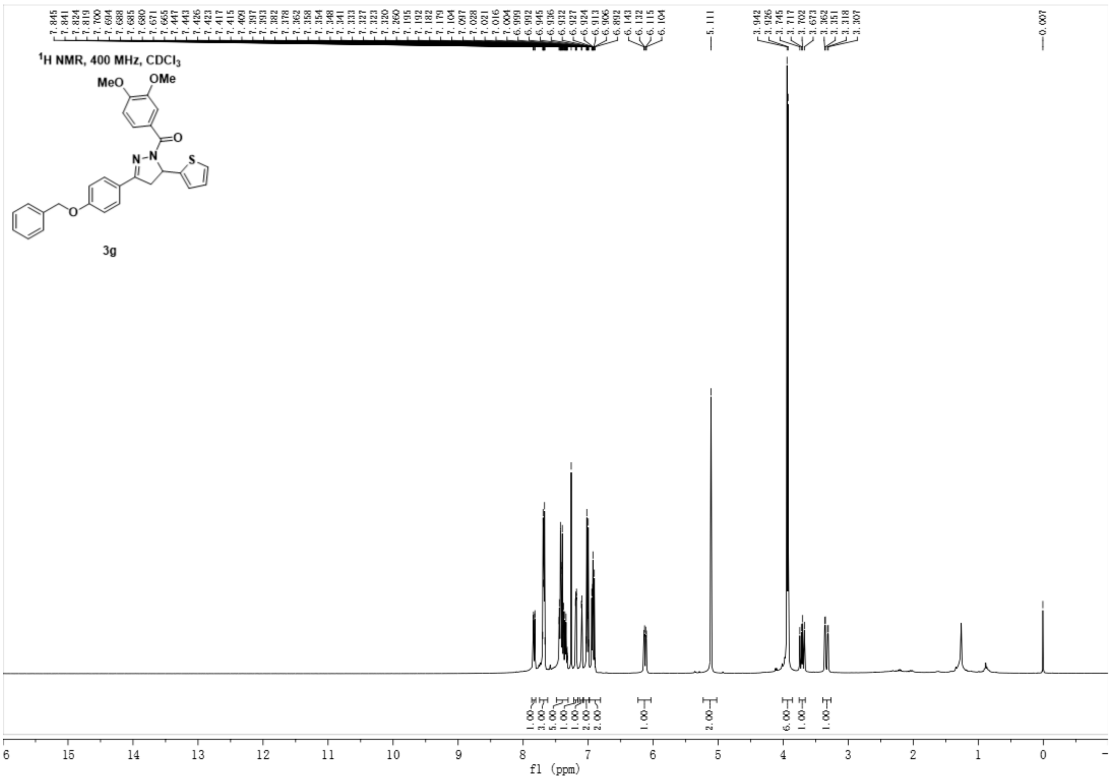

^{1}H NMR, 400 MHz, CDCl$_3$
MeO
OMe
3g
f1 (ppm)

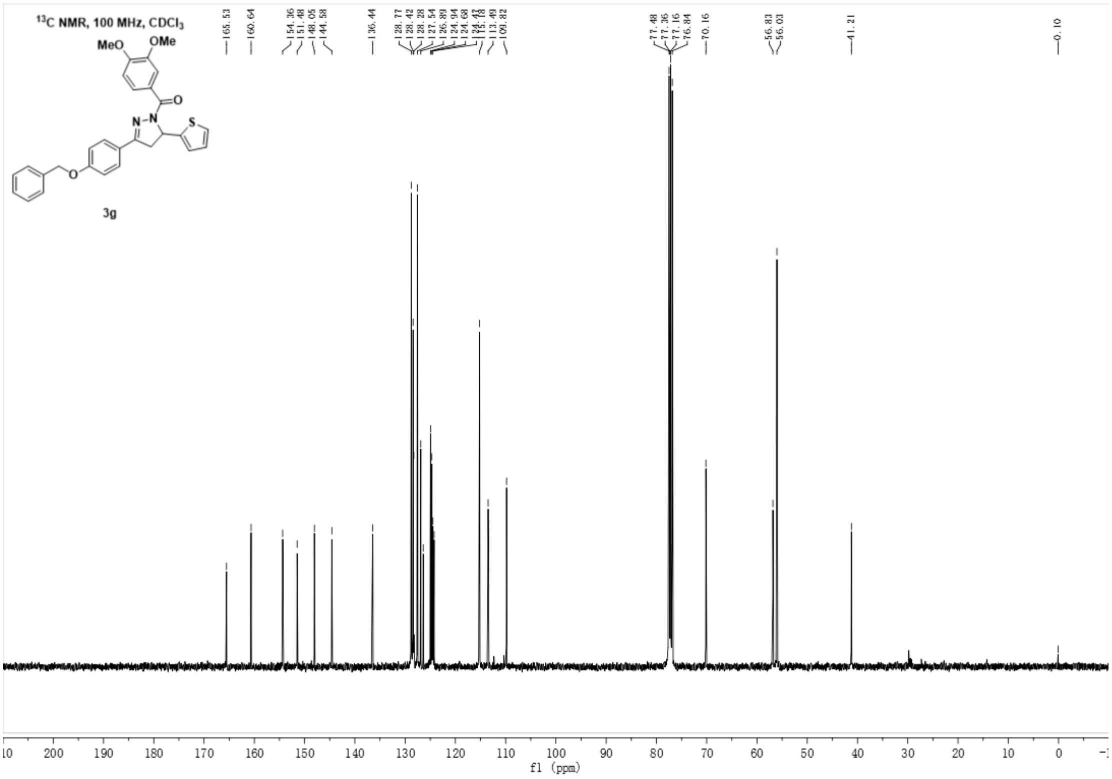

^{13}C NMR, 100 MHz, CDCl$_3$
MeO
OMe
3g
f1 (ppm)

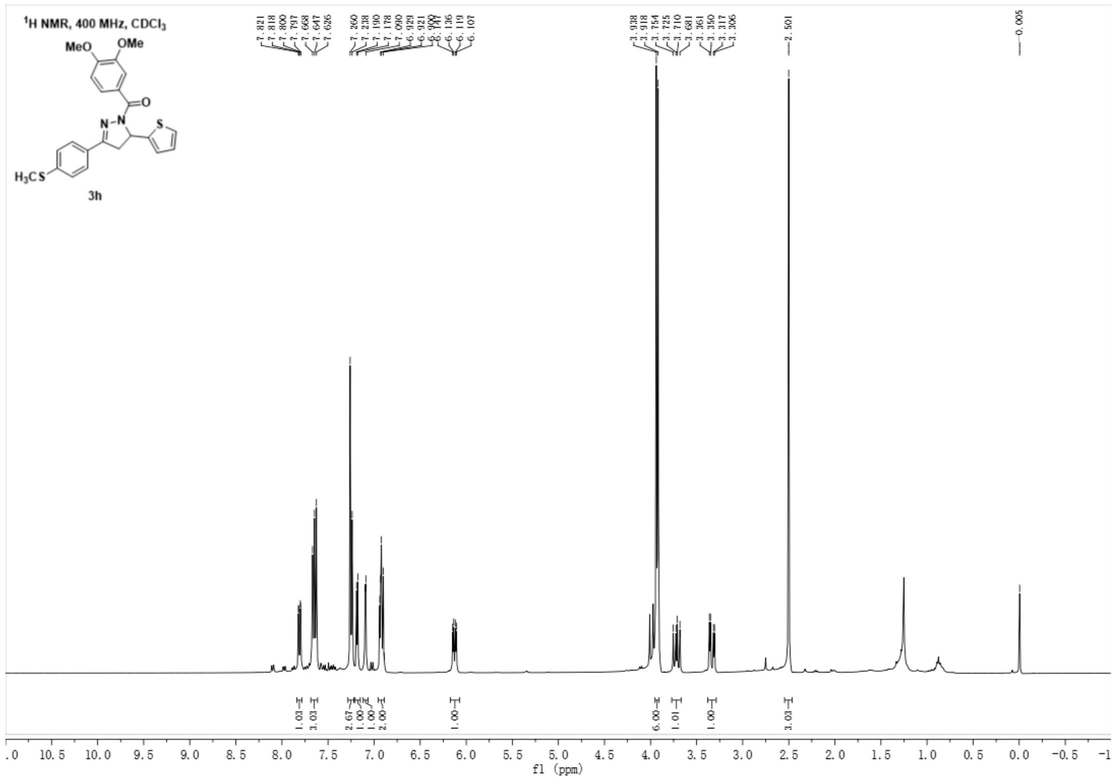

^{1}H NMR, 400 MHz, CDCl$_3$
MeO
OMe
O
N
N
S
H_3CS
3h
f1 (ppm)

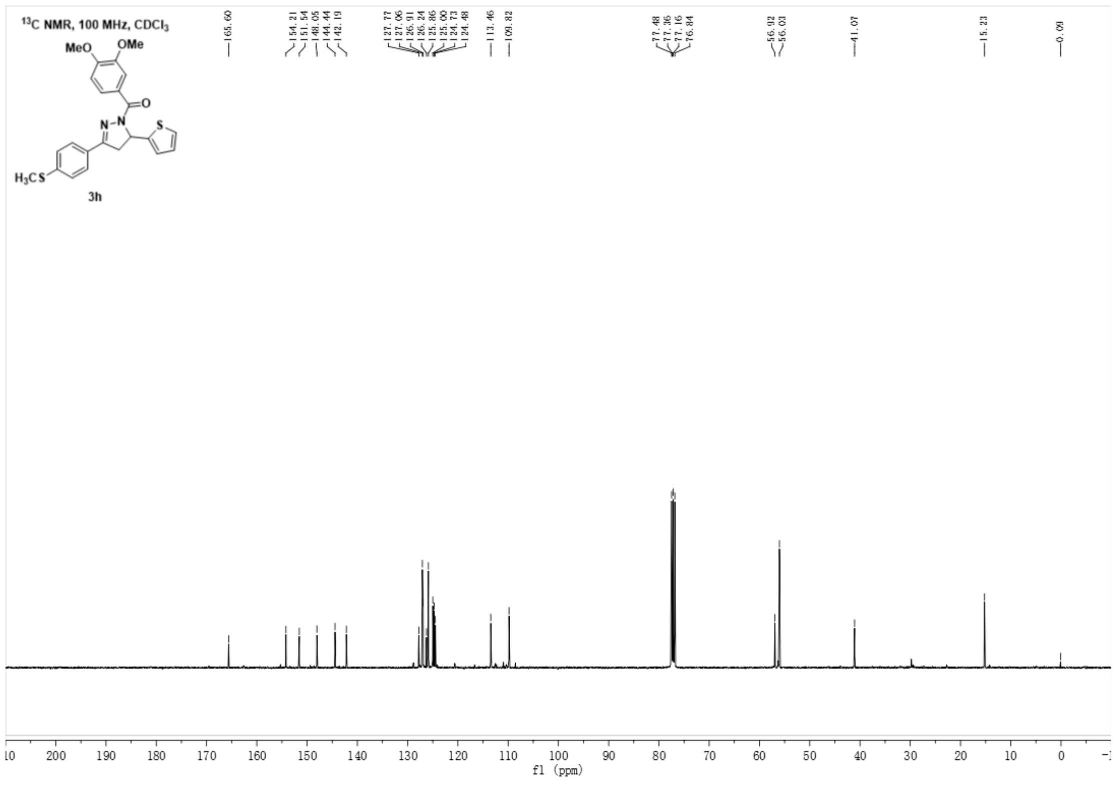

^{13}C NMR, 100 MHz, CDCl$_3$
MeO
OMe
O
N
N
S
H_3CS
3h
165.60
154.21
151.54
148.05
144.44
142.19
127.77
127.06
126.91
126.24
125.86
125.00
124.73
124.48
113.46
109.82
77.48
77.36
77.16
76.84
56.92
56.03
41.07
15.23
0.09
f1 (ppm)

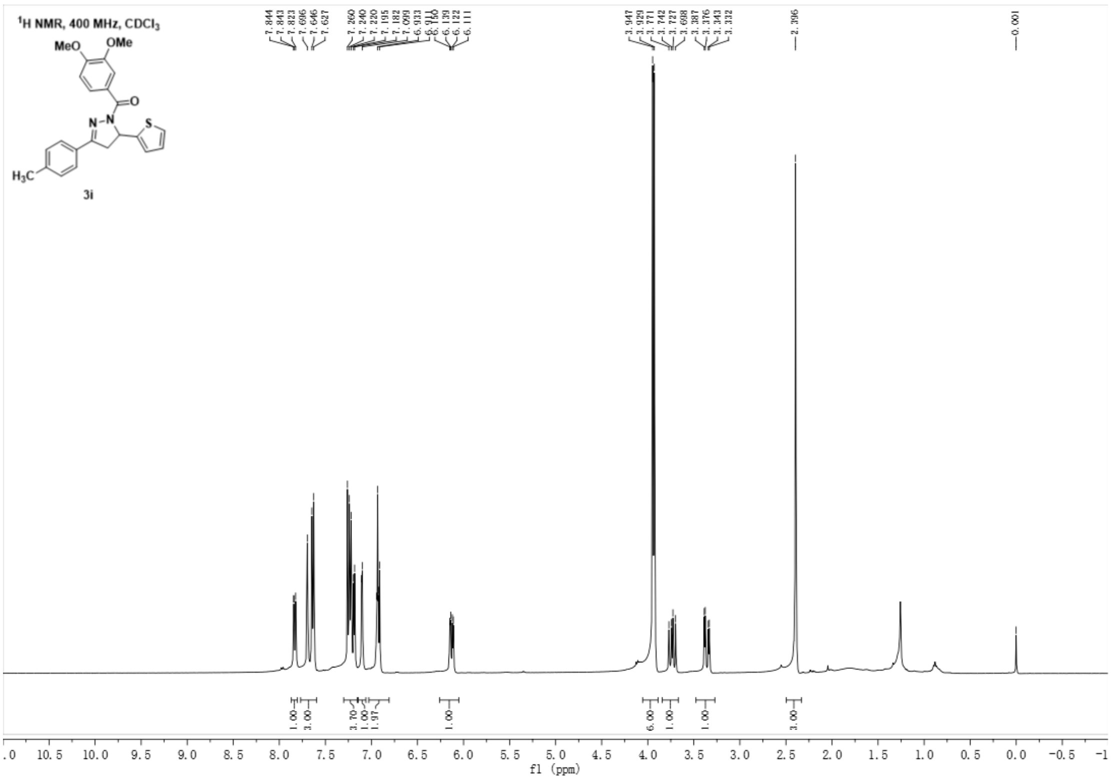

1H NMR, 400 MHz, CDCl3
MeO
OMe
O
N
N
S
H3C
3i
f1 (ppm)

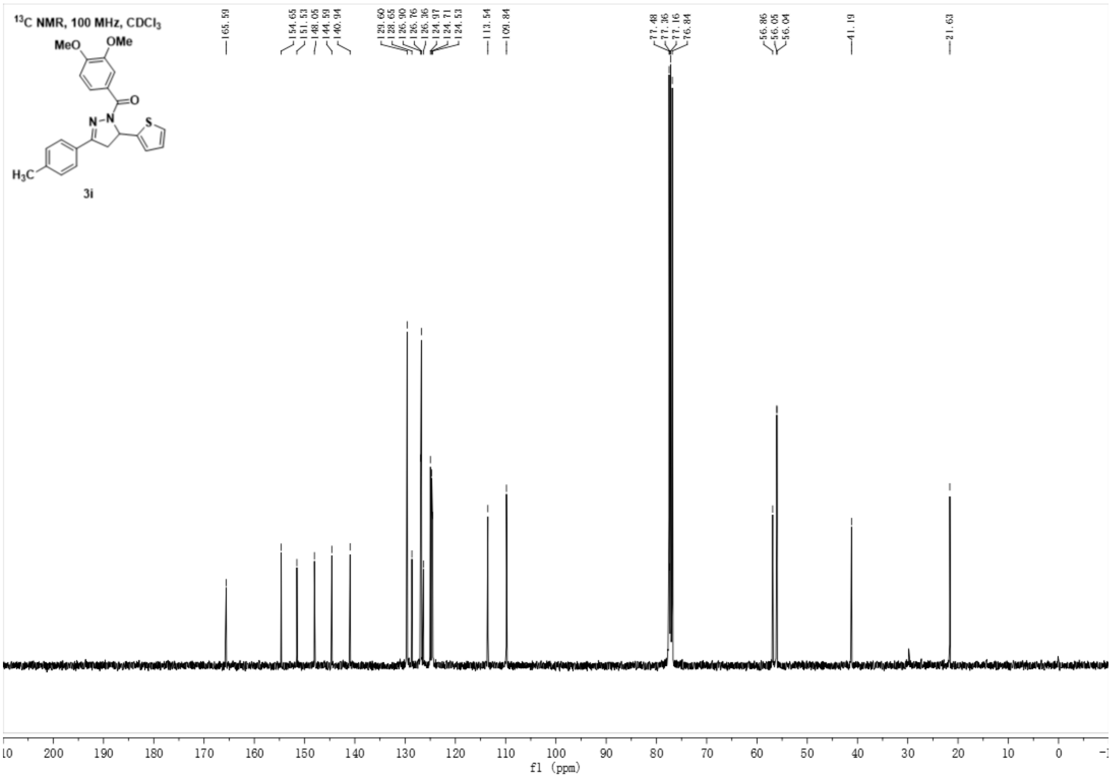

13C NMR, 100 MHz, CDCl3
MeO
OMe
O
N
N
S
H3C
3i
f1 (ppm)

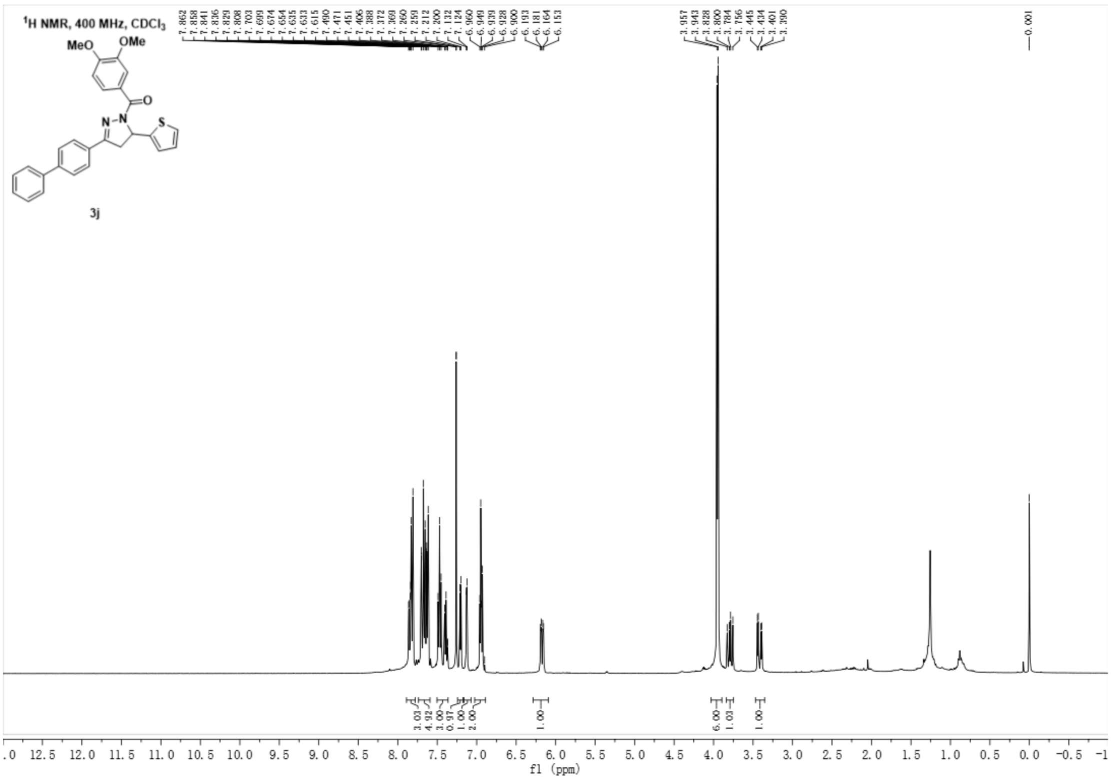

1H NMR, 400 MHz, CDCl3
MeO
OMe
O
N
N
S
3j
7.862
7.858
7.841
7.836
7.829
7.808
7.703
7.699
7.674
7.654
7.635
7.633
7.615
7.490
7.471
7.451
7.406
7.388
7.372
7.369
7.260
7.259
7.212
7.200
7.132
7.124
6.960
6.949
6.939
6.928
6.900
6.193
6.181
6.164
6.153
3.957
3.943
3.828
3.800
3.784
3.756
3.445
3.434
3.401
3.390
0.001
3.03
4.92
3.00
0.97
1.00
2.00
1.00
6.00
1.03
1.00
fl (ppm)

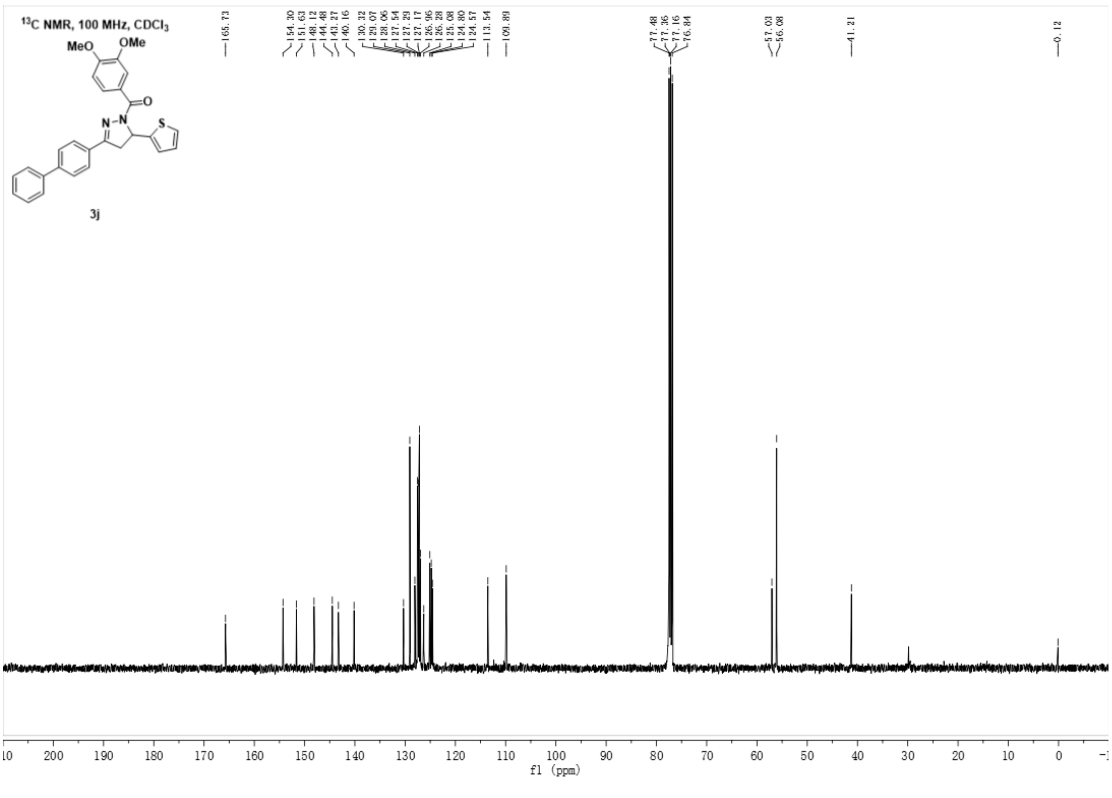

13C NMR, 100 MHz, CDCl3
MeO
OMe
O
N
N
S
3j
165.73
154.30
151.63
148.12
144.48
143.27
140.16
130.32
129.07
128.06
127.54
127.29
127.17
126.96
126.28
125.08
124.80
124.57
113.54
109.89
77.48
77.36
77.16
76.84
57.03
56.08
41.21
0.12
fl (ppm)

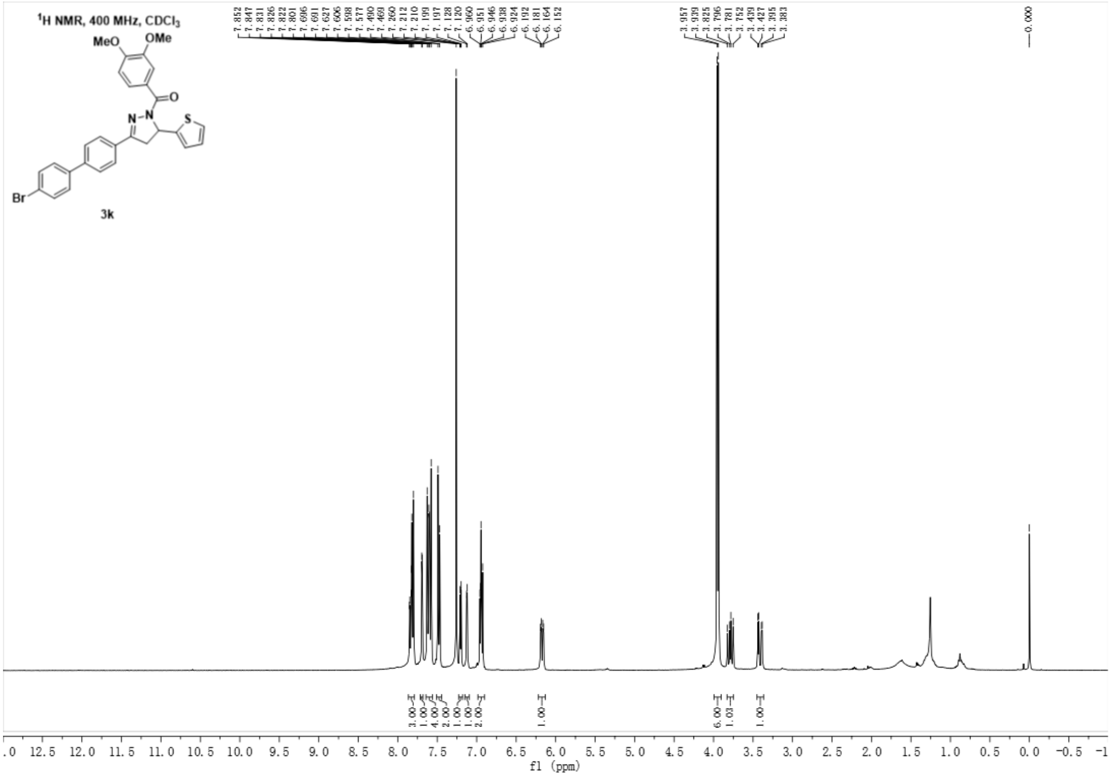
1H NMR, 400 MHz, CDCl3
MeO
OMe
Br
3k
7.852 7.847 7.831 7.826 7.822 7.801 7.696 7.691 7.627 7.606 7.598 7.577 7.490 7.469 7.260 7.212 7.210 7.199 7.197 7.128 7.120 6.960 6.951 6.946 6.938 6.924 6.192 6.181 6.164 6.152
3.957 3.929 3.825 3.796 3.781 3.752 3.439 3.427 3.395 3.383
0.000
3.00 1.00 1.00 4.00 2.00 1.00 1.00 2.00
1.00
6.00 1.03 1.00
f1 (ppm)

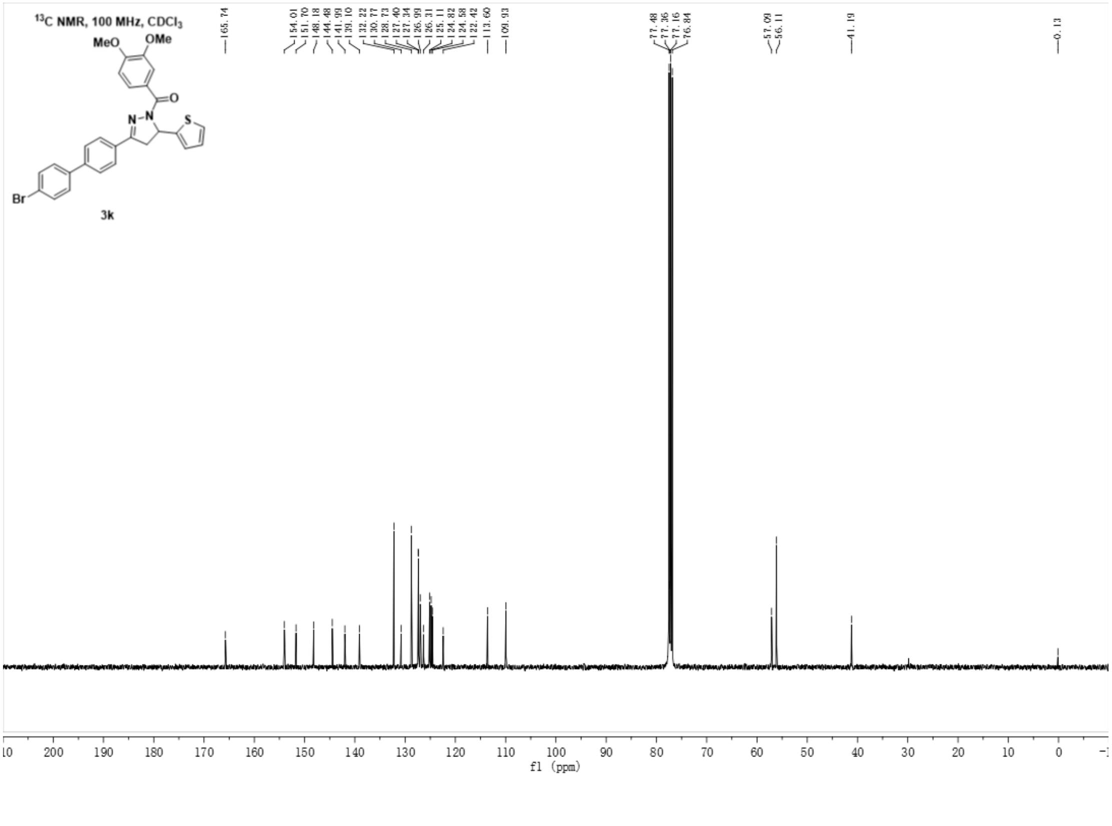
13C NMR, 100 MHz, CDCl3
MeO
OMe
Br
3k
165.74
154.01 151.70 148.18 144.48 141.99 139.10 132.22 130.77 128.73 127.40 127.34 126.99 126.31 125.11 124.82 124.58 122.42 113.60 109.93
77.48 77.36 77.16 76.84
57.09 56.11
41.19
0.13
f1 (ppm)

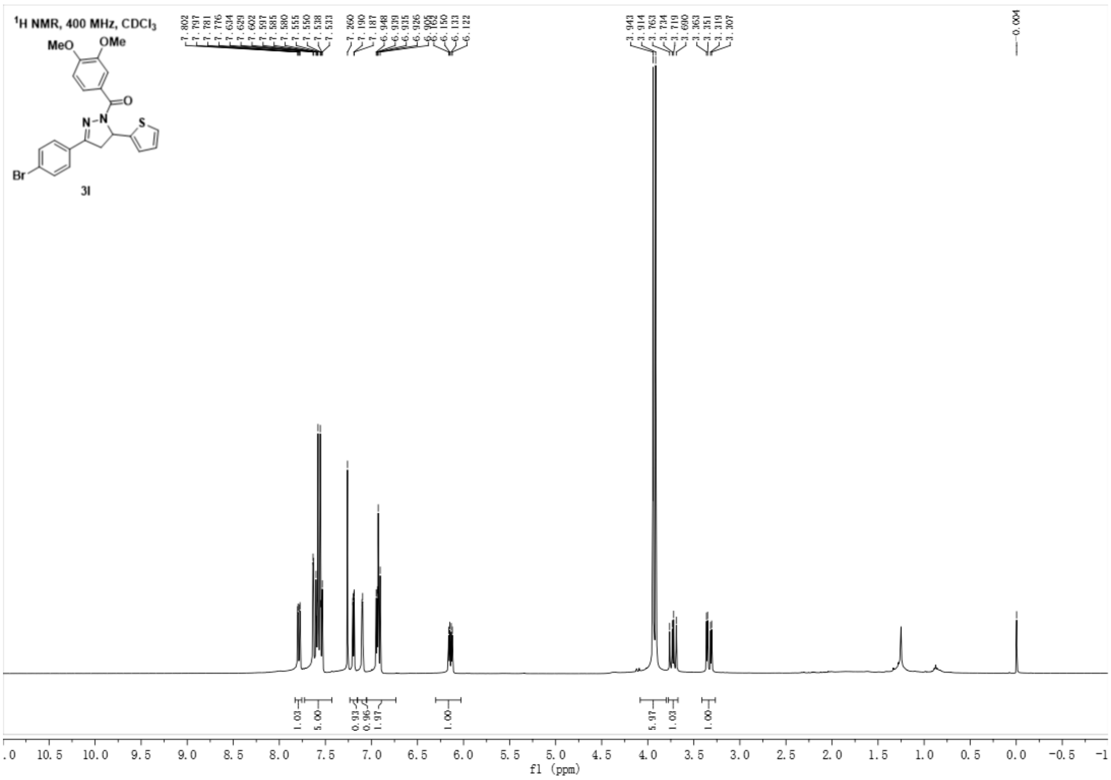
1H NMR, 400 MHz, CDCl3
MeO
OMe
Br
3l
f1 (ppm)

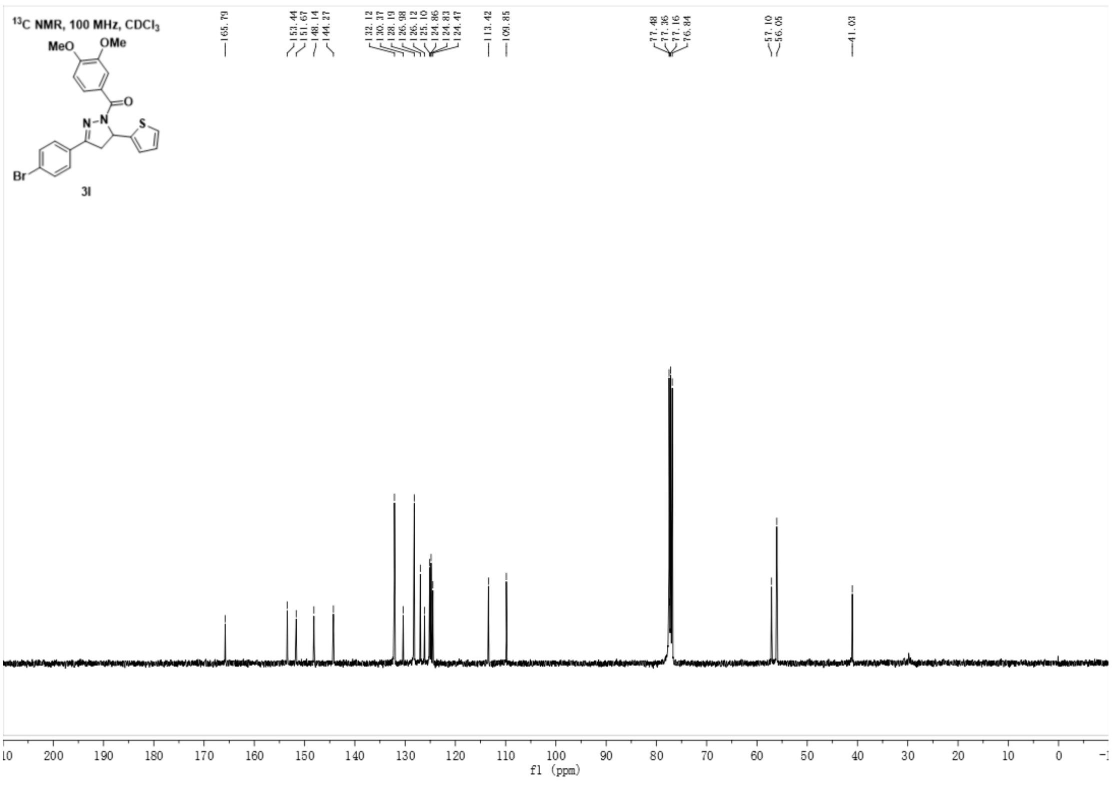
13C NMR, 100 MHz, CDCl3
MeO
OMe
Br
3l
f1 (ppm)

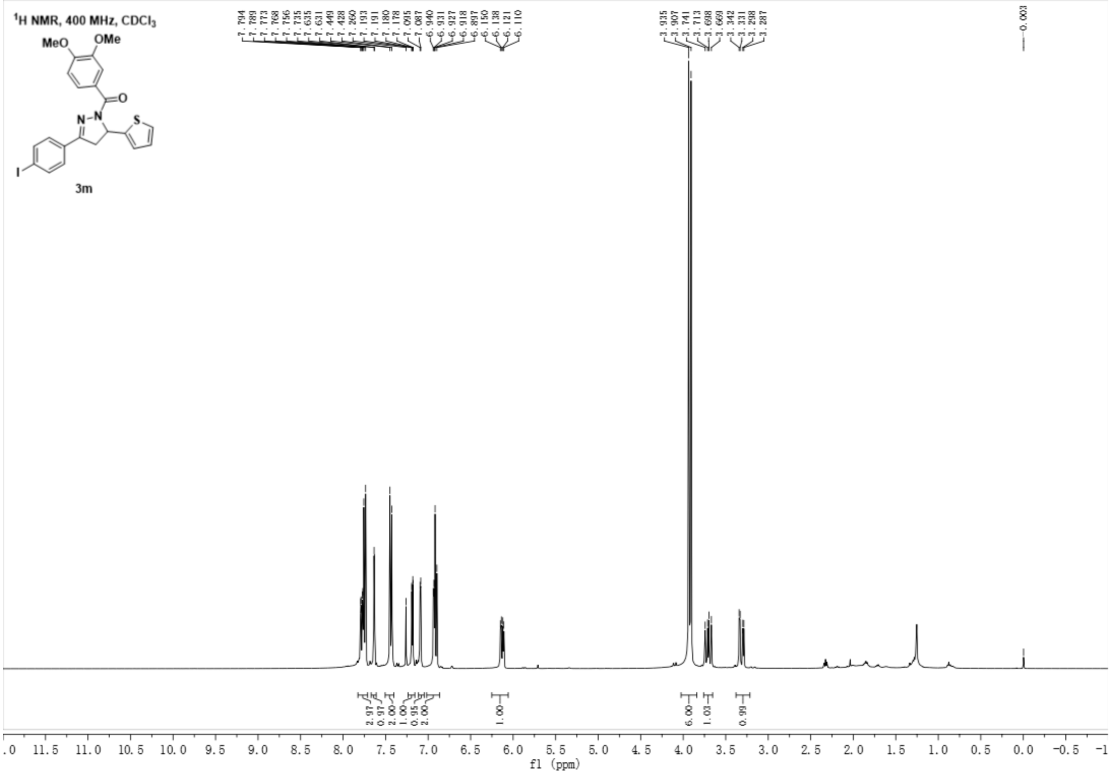

¹H NMR, 400 MHz, CDCl3
MeO
OMe
3m
f1 (ppm)

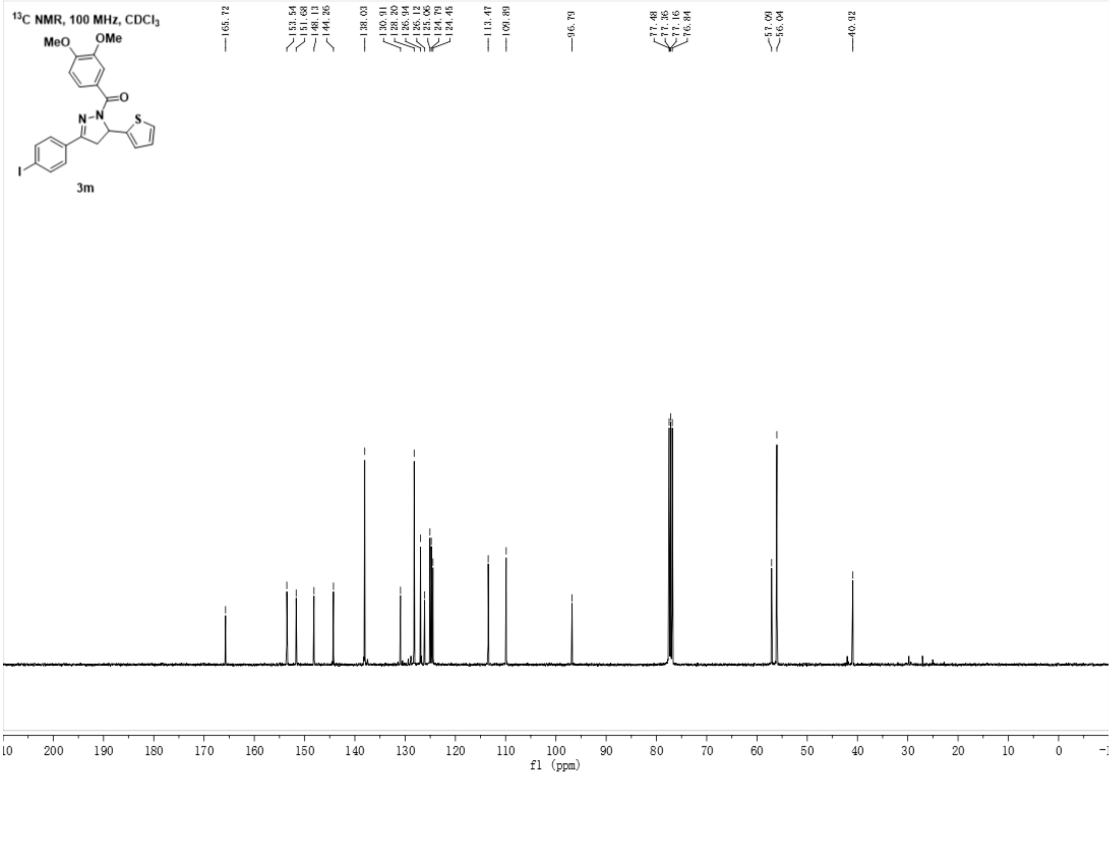

¹³C NMR, 100 MHz, CDCl3
MeO
OMe
3m
f1 (ppm)

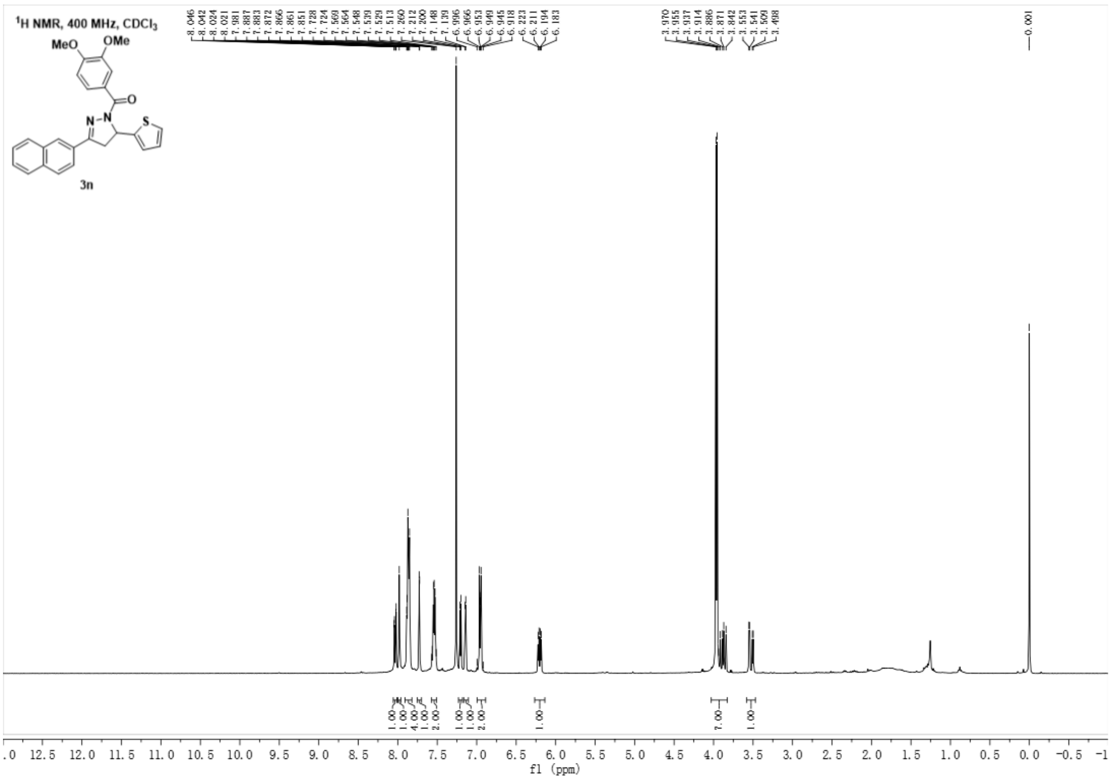
1H NMR, 400 MHz, CDCl3
MeO
OMe
3n
f1 (ppm)

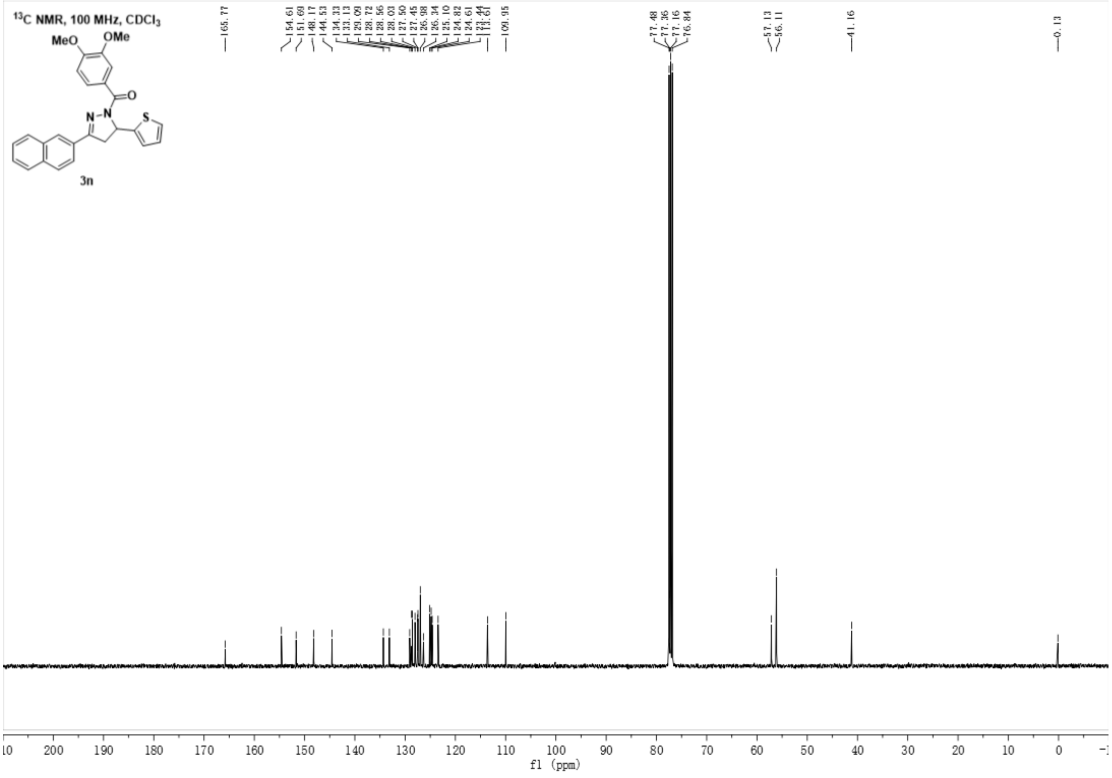
13C NMR, 100 MHz, CDCl3
MeO
OMe
3n
f1 (ppm)

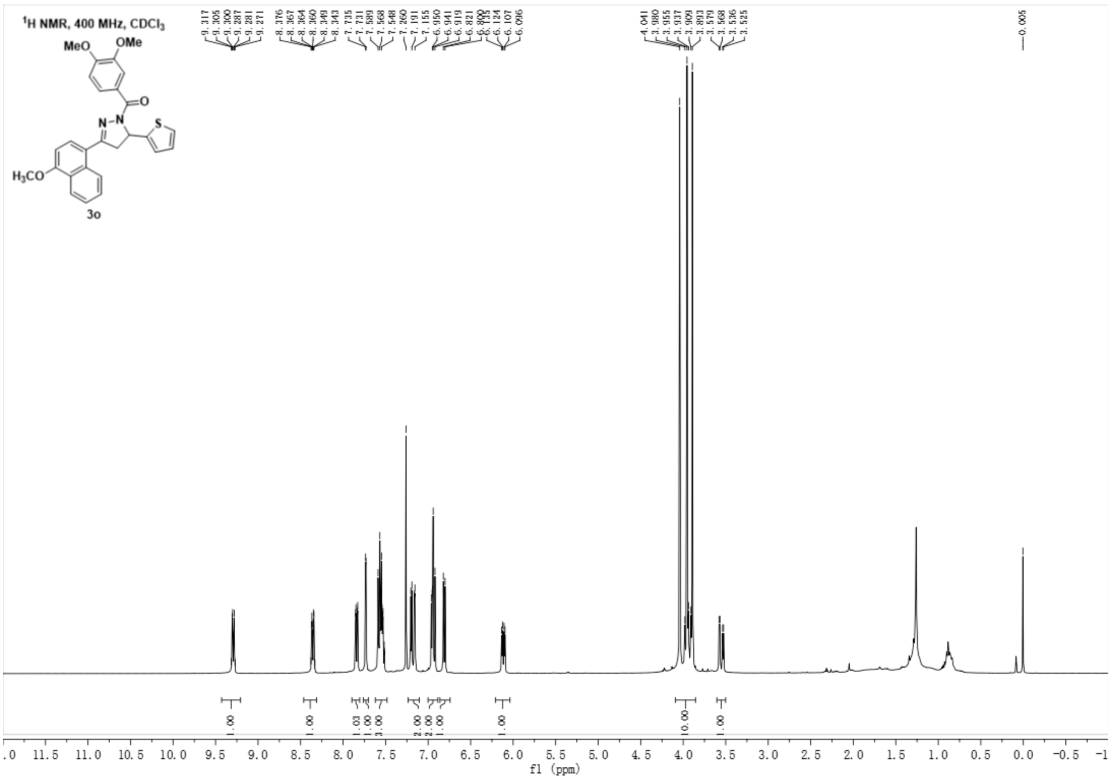

1H NMR, 400 MHz, CDCl3
MeO
OMe
O
N
N
S
H3CO
3o
f1 (ppm)

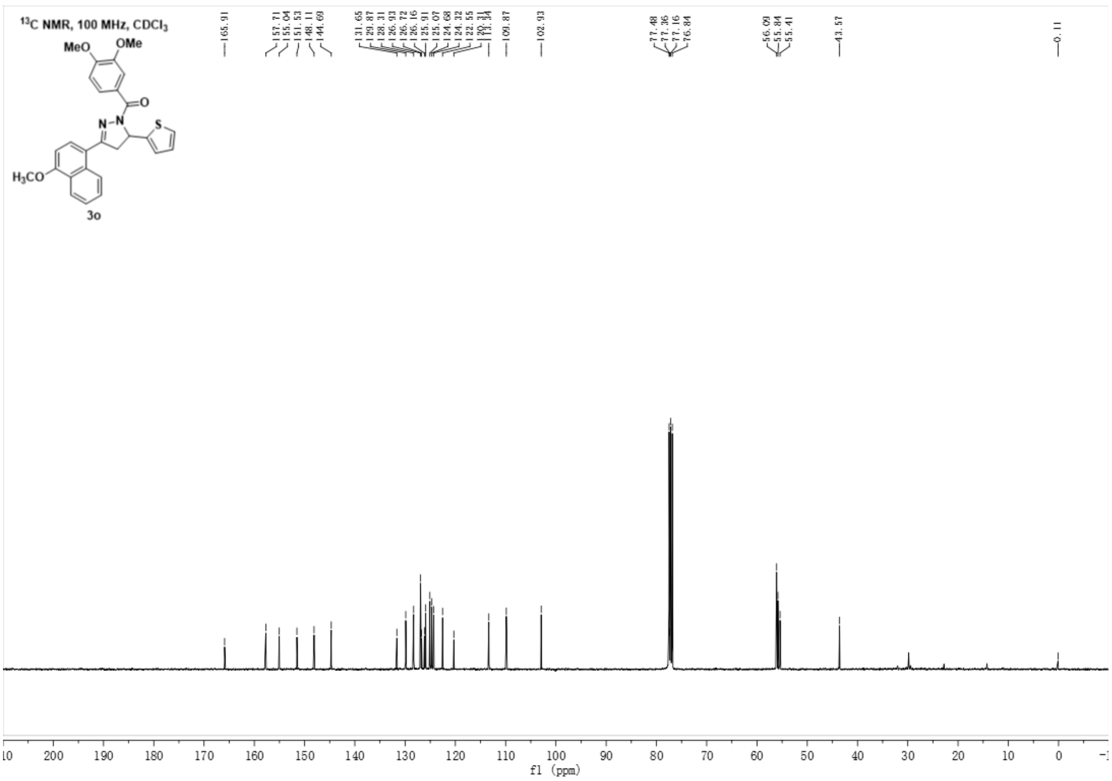

13C NMR, 100 MHz, CDCl3
MeO
OMe
O
N
N
S
H3CO
3o
f1 (ppm)

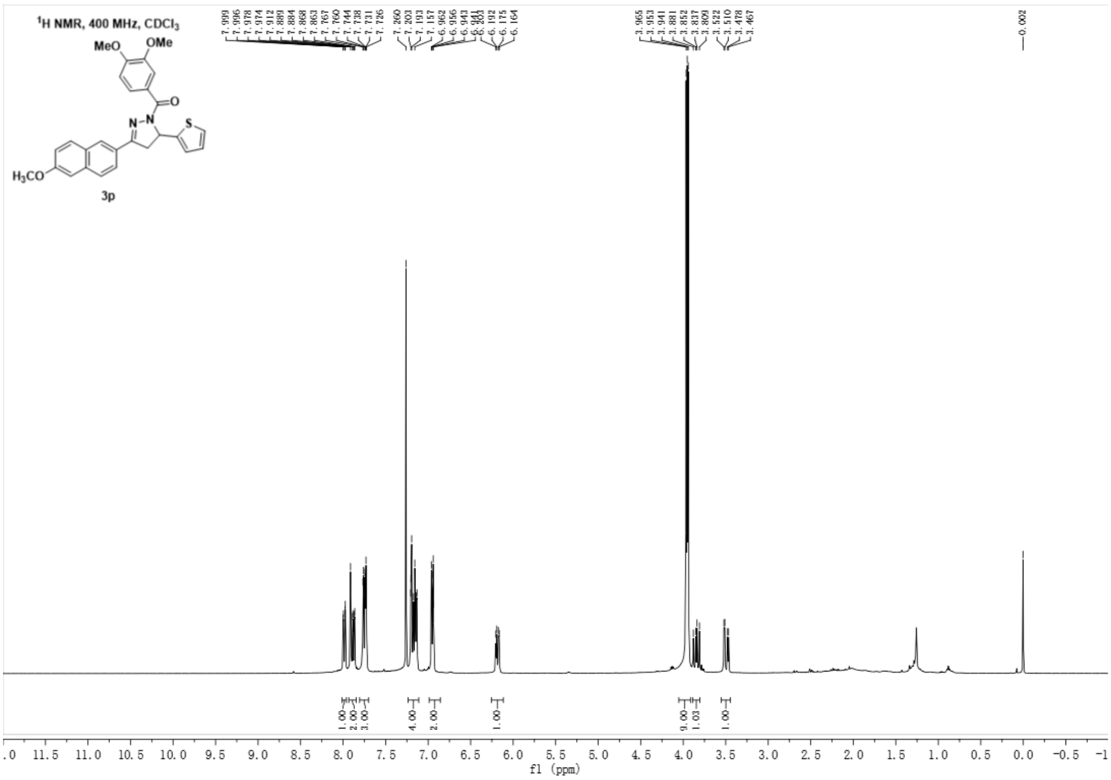

^{1}H NMR, 400 MHz, CDCl$_3$
MeO
OMe
H$_3$CO
3p
f1 (ppm)

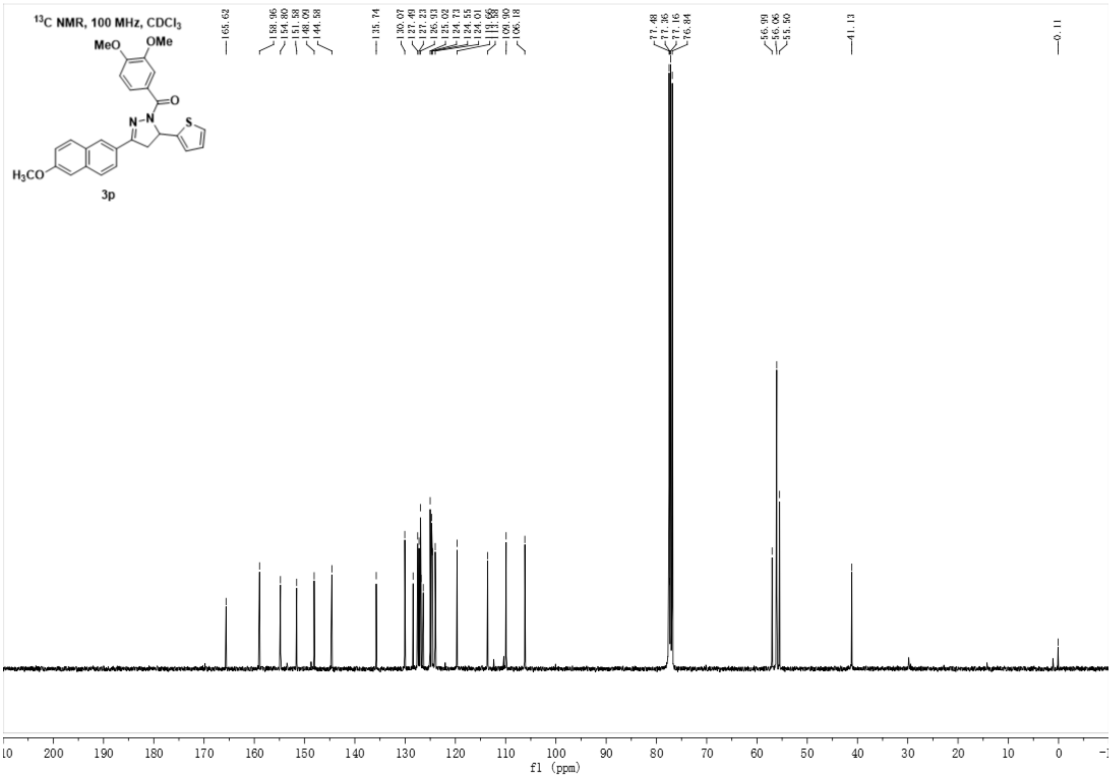

^{13}C NMR, 100 MHz, CDCl$_3$
MeO
OMe
H$_3$CO
3p
f1 (ppm)

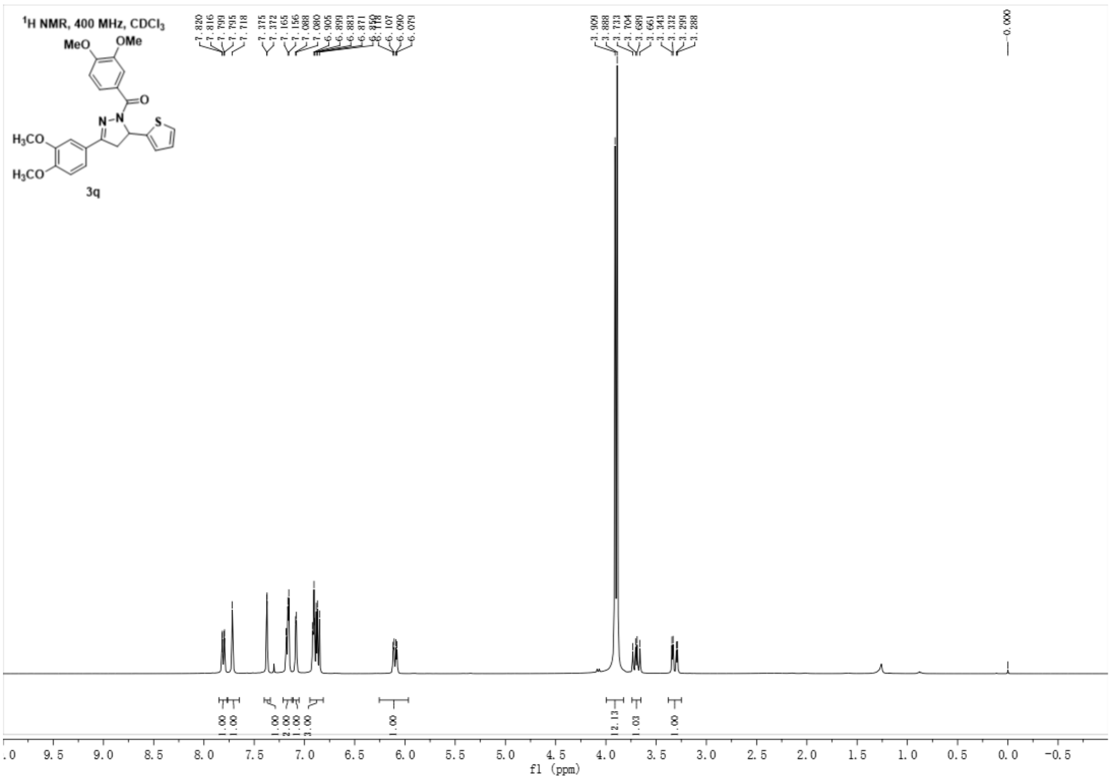

^{1}H NMR, 400 MHz, CDCl$_3$
MeO
OMe
O
N–N
S
H$_3$CO
H$_3$CO
3q
f1 (ppm)

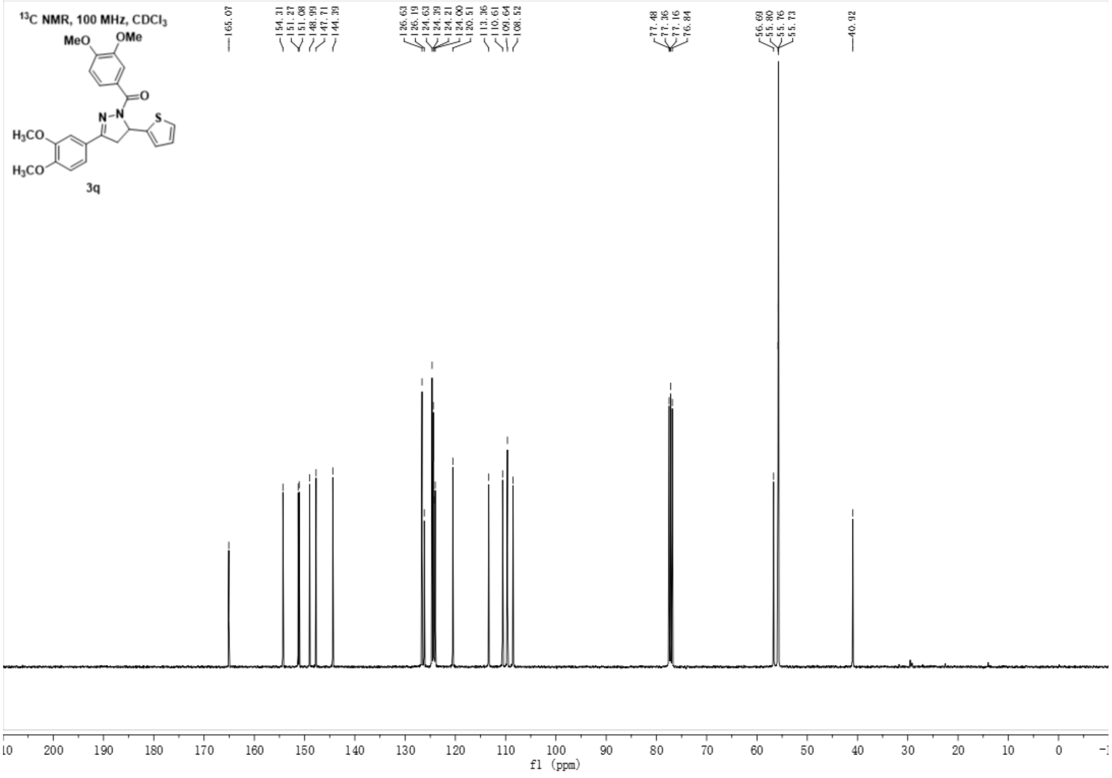

^{13}C NMR, 100 MHz, CDCl$_3$
MeO
OMe
O
N–N
S
H$_3$CO
H$_3$CO
3q
f1 (ppm)

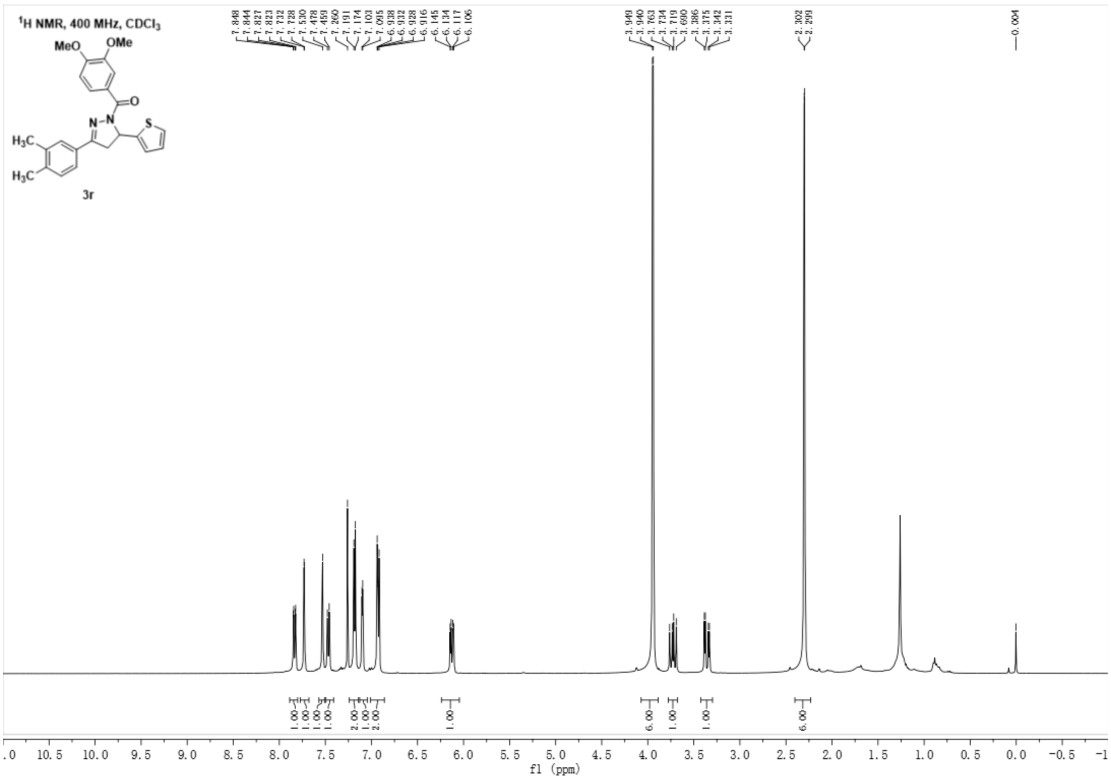
1H NMR, 400 MHz, CDCl3
MeO
OMe
N
N
O
S
H3C
H3C
3r
f1 (ppm)

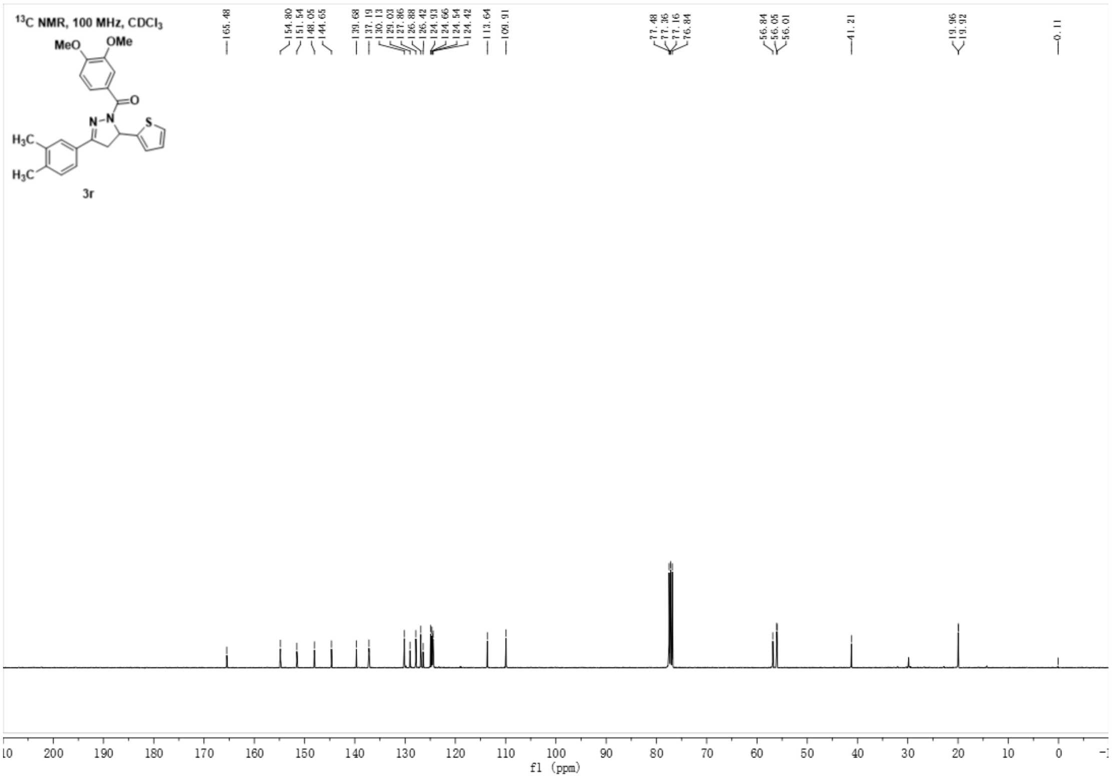
13C NMR, 100 MHz, CDCl3
MeO
OMe
N
N
O
S
H3C
H3C
3r
f1 (ppm)

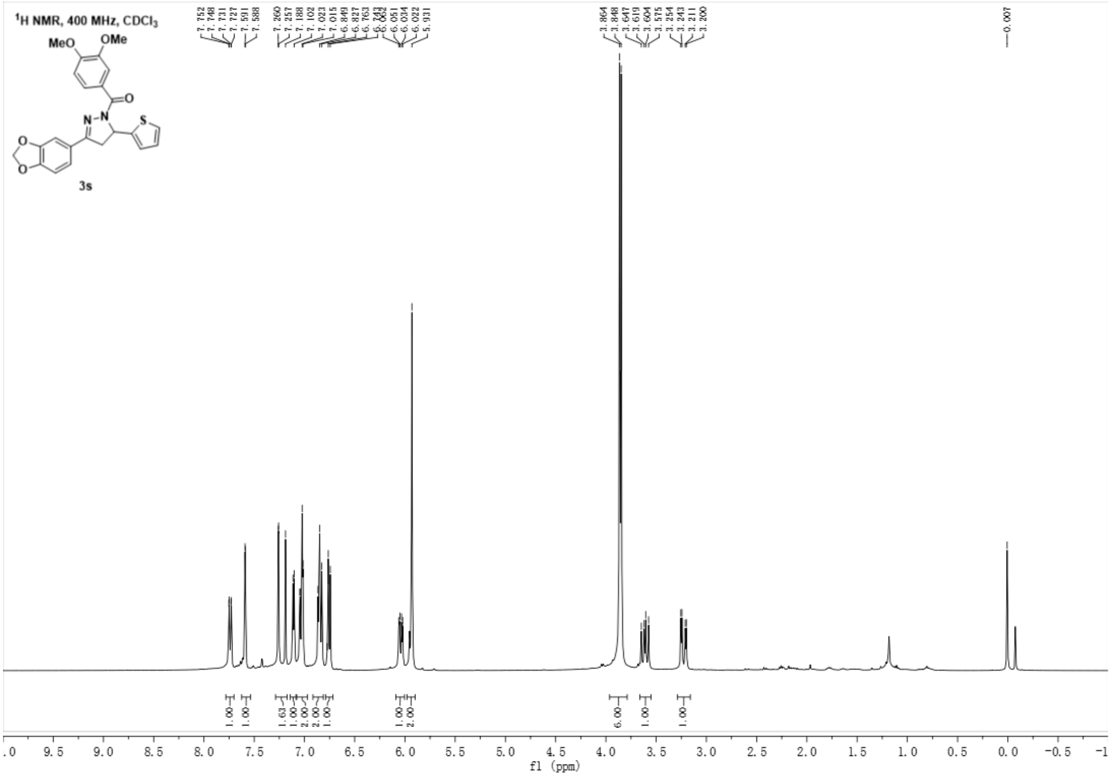

¹H NMR, 400 MHz, CDCl₃
MeO
OMe
3s
f1 (ppm)

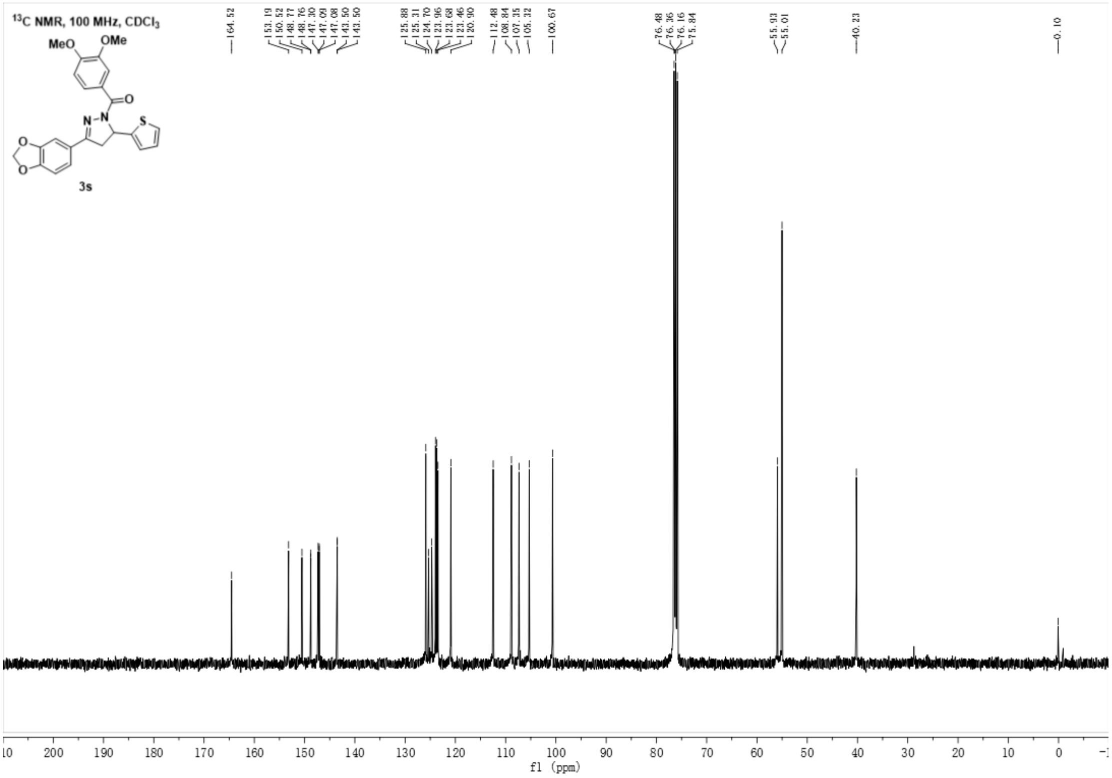

¹³C NMR, 100 MHz, CDCl₃
MeO
OMe
3s
f1 (ppm)

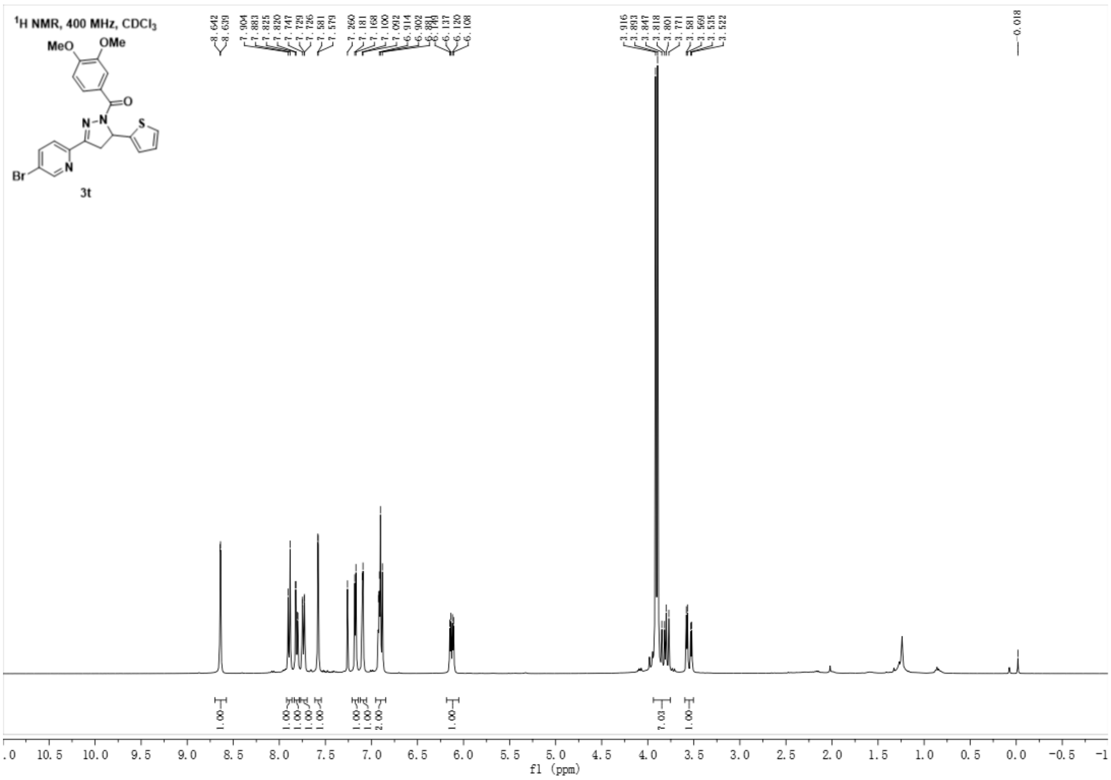

^{1}H NMR, 400 MHz, CDCl$_3$
MeO
OMe
O
N
N
S
Br
N
3t
f1 (ppm)

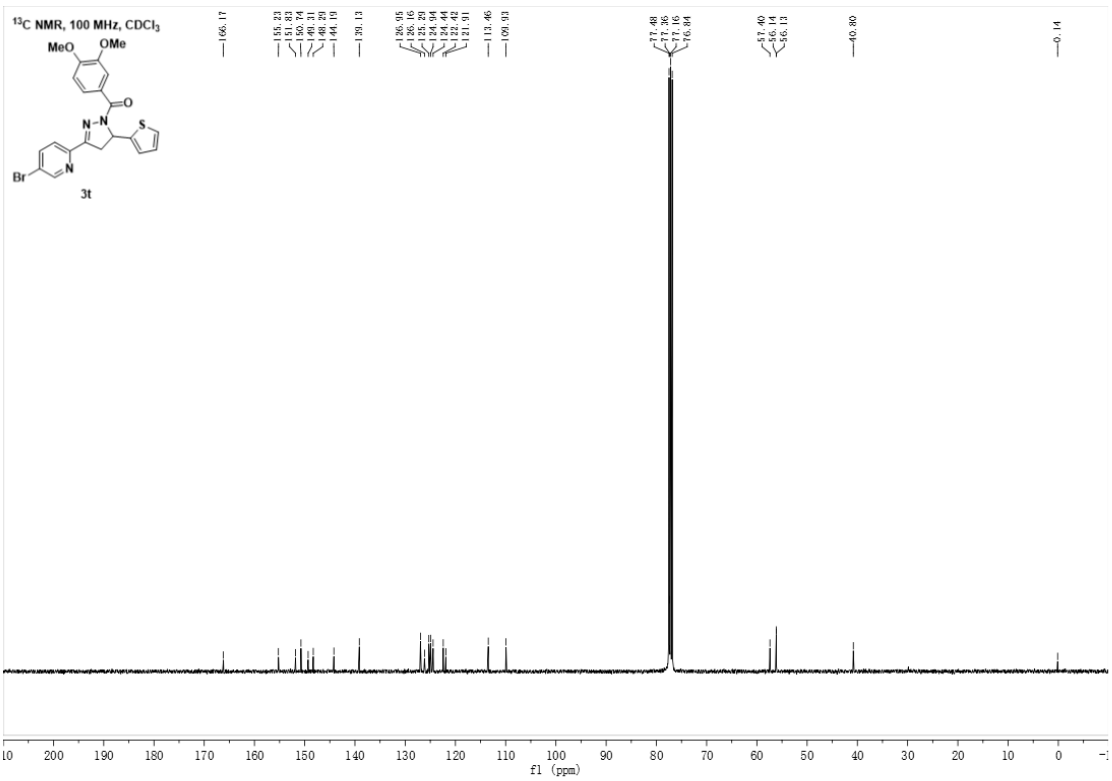

^{13}C NMR, 100 MHz, CDCl$_3$
MeO
OMe
O
N
N
S
Br
N
3t
166.17
155.23
151.83
150.74
149.31
148.29
144.19
139.13
126.95
126.16
125.29
124.94
124.44
122.42
121.91
113.46
109.93
77.48
77.36
77.16
76.84
57.40
56.14
56.13
40.80
0.14
f1 (ppm)

HRMS analytical data

3a:

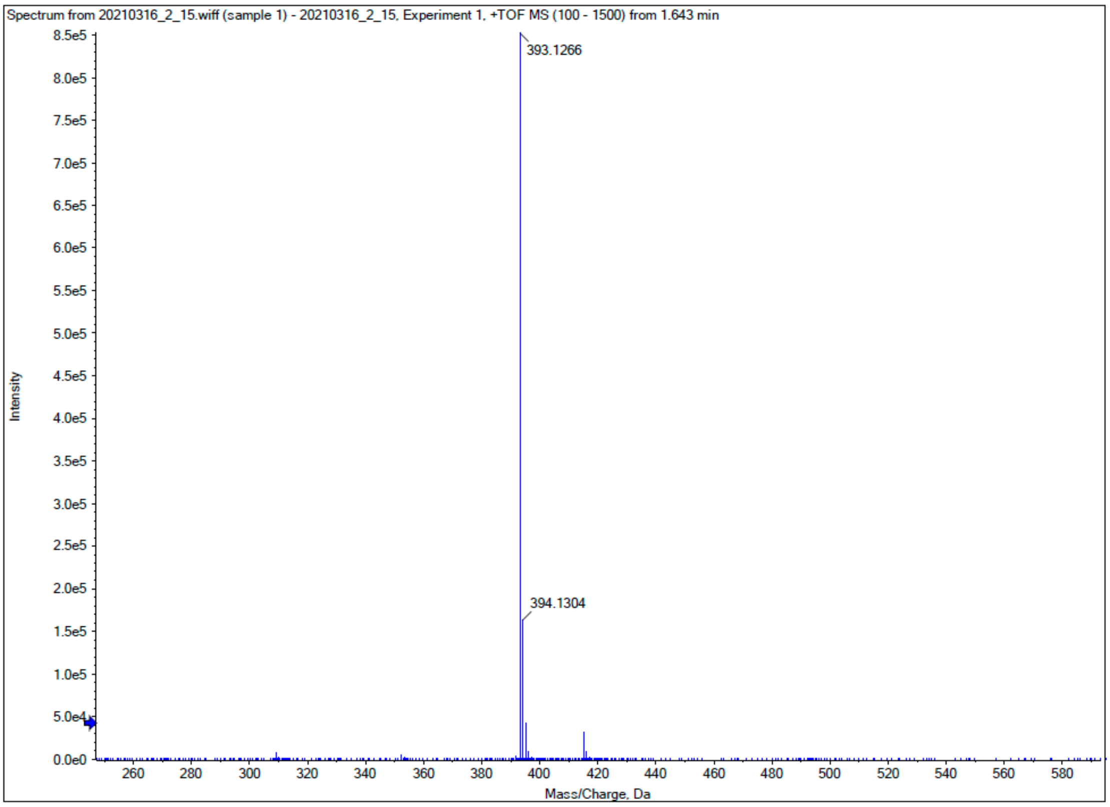

3b:

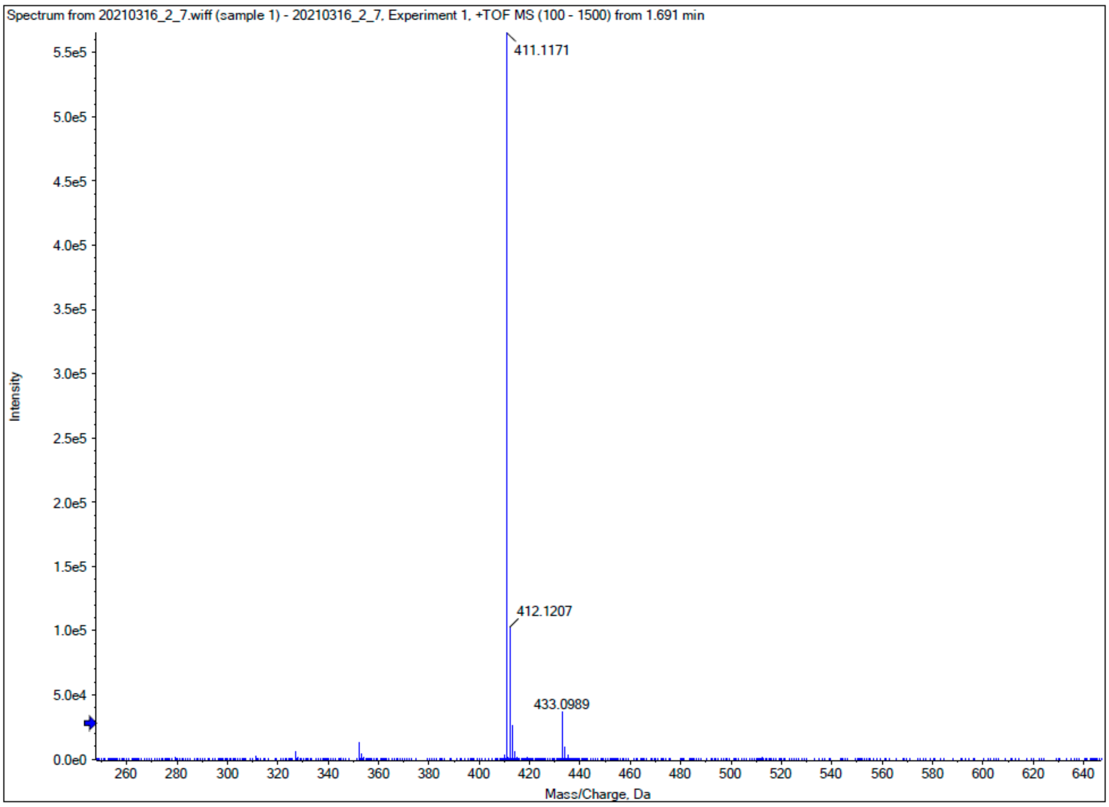

3c:

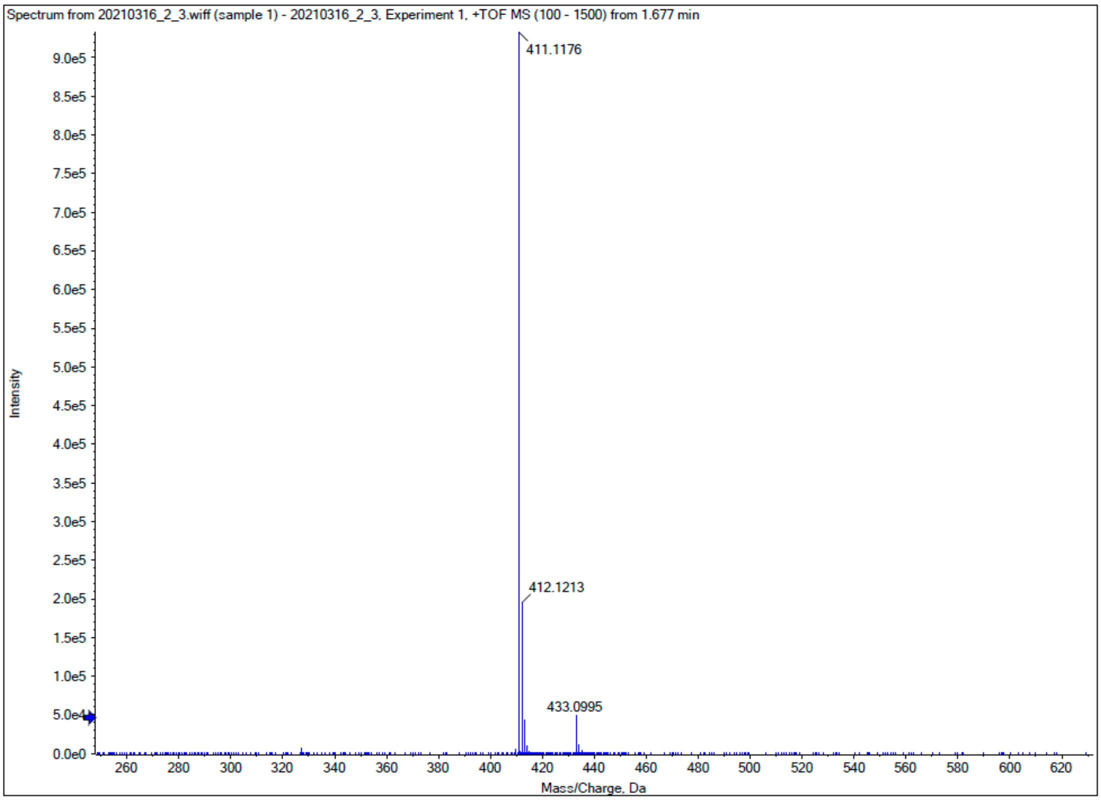
Spectrum from 20210316_2_3.wiff (sample 1) - 20210316_2_3, Experiment 1, +TOF MS (100 - 1500) from 1.677 min
411.1176
412.1213
433.0995
Intensity
Mass/Charge, Da

3d:

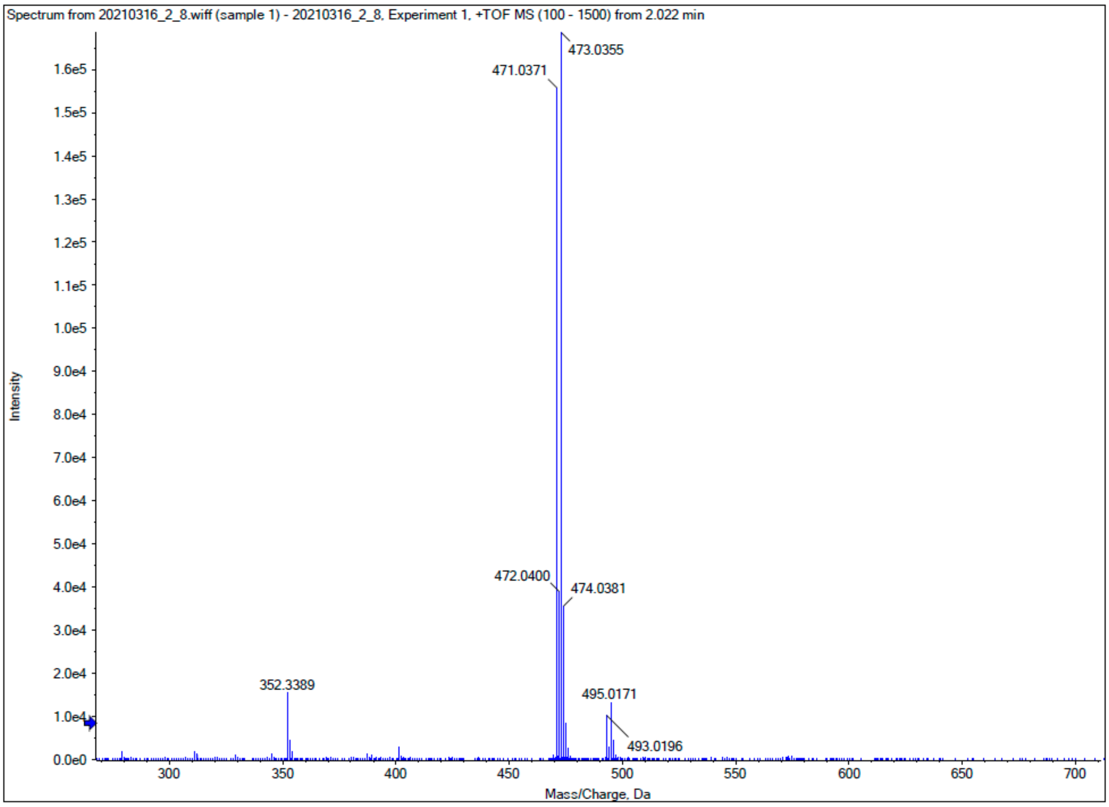
Spectrum from 20210316_2_8.wiff (sample 1) - 20210316_2_8, Experiment 1, +TOF MS (100 - 1500) from 2.022 min
473.0355
471.0371
472.0400
474.0381
352.3389
495.0171
493.0196
Intensity
Mass/Charge, Da

3e:

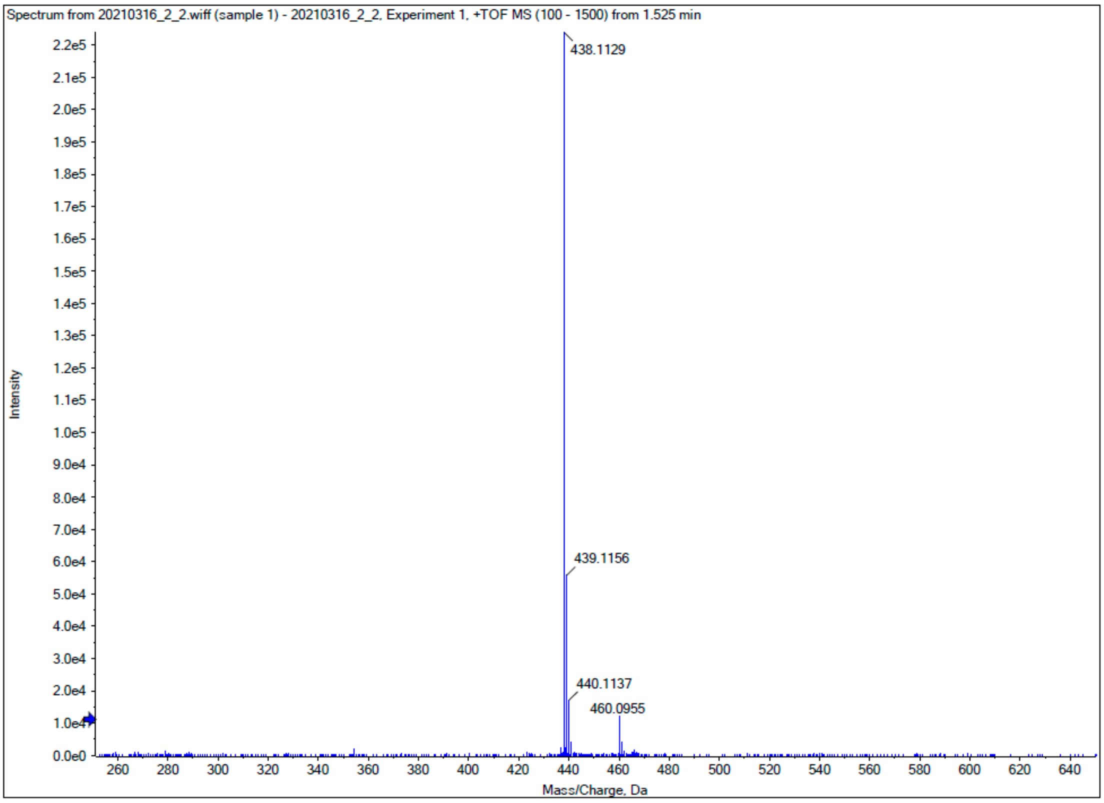
Spectrum from 20210316_2_2.wiff (sample 1) - 20210316_2_2, Experiment 1, +TOF MS (100 - 1500) from 1.525 min
438.1129
439.1156
440.1137
460.0955
Intensity
Mass/Charge, Da

3f:

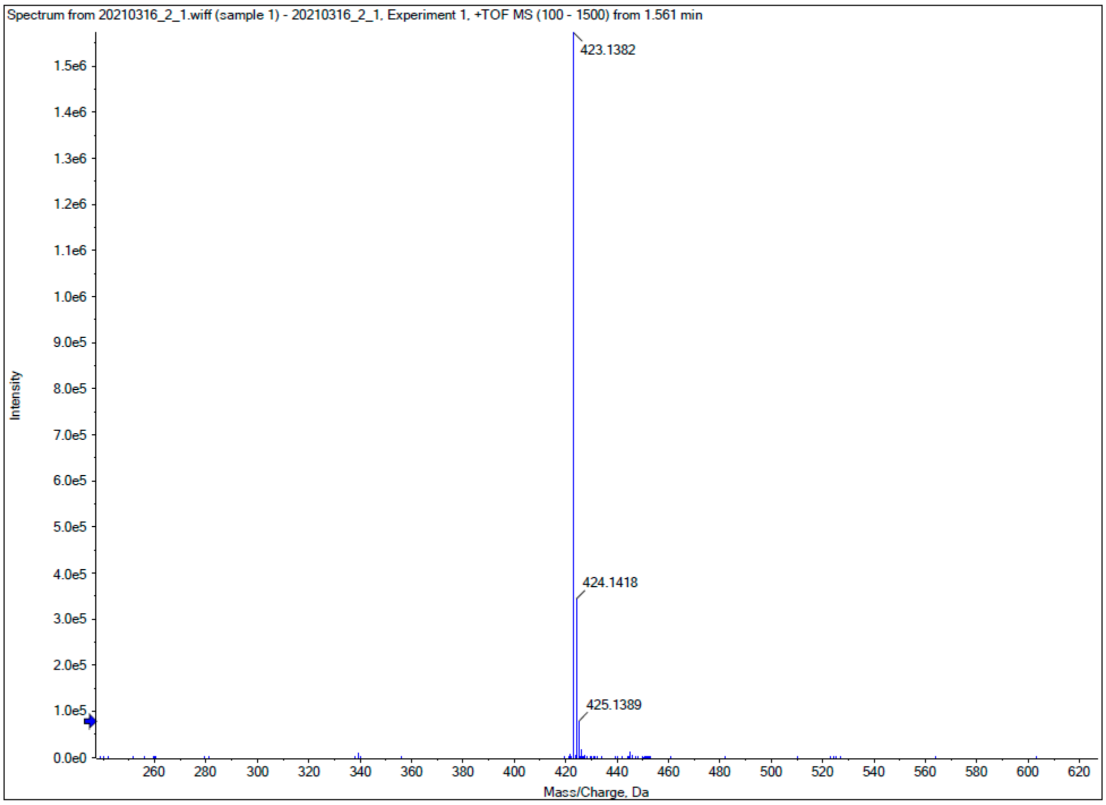
Spectrum from 20210316_2_1.wiff (sample 1) - 20210316_2_1, Experiment 1, +TOF MS (100 - 1500) from 1.561 min
423.1382
424.1418
425.1389
Intensity
Mass/Charge, Da

3g:

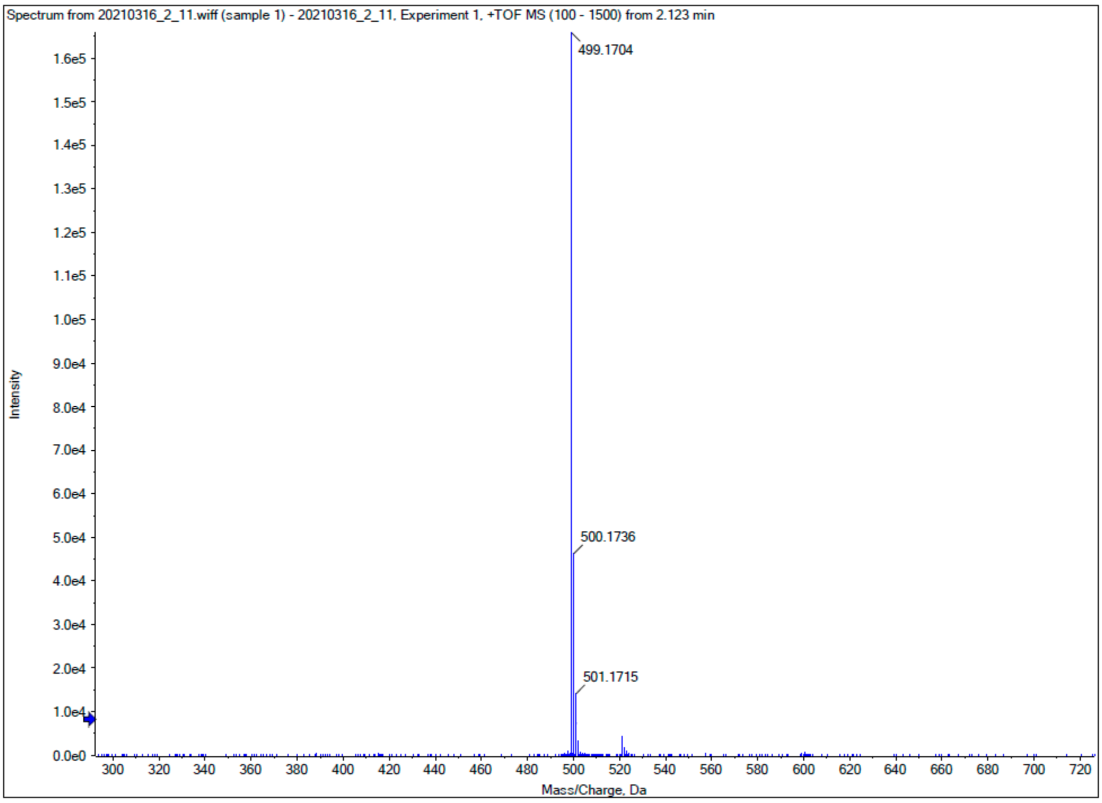
Spectrum from 20210316_2_11.wiff (sample 1) - 20210316_2_11, Experiment 1, +TOF MS (100 - 1500) from 2.123 min
499.1704
500.1736
501.1715
Intensity
Mass/Charge, Da

3h:

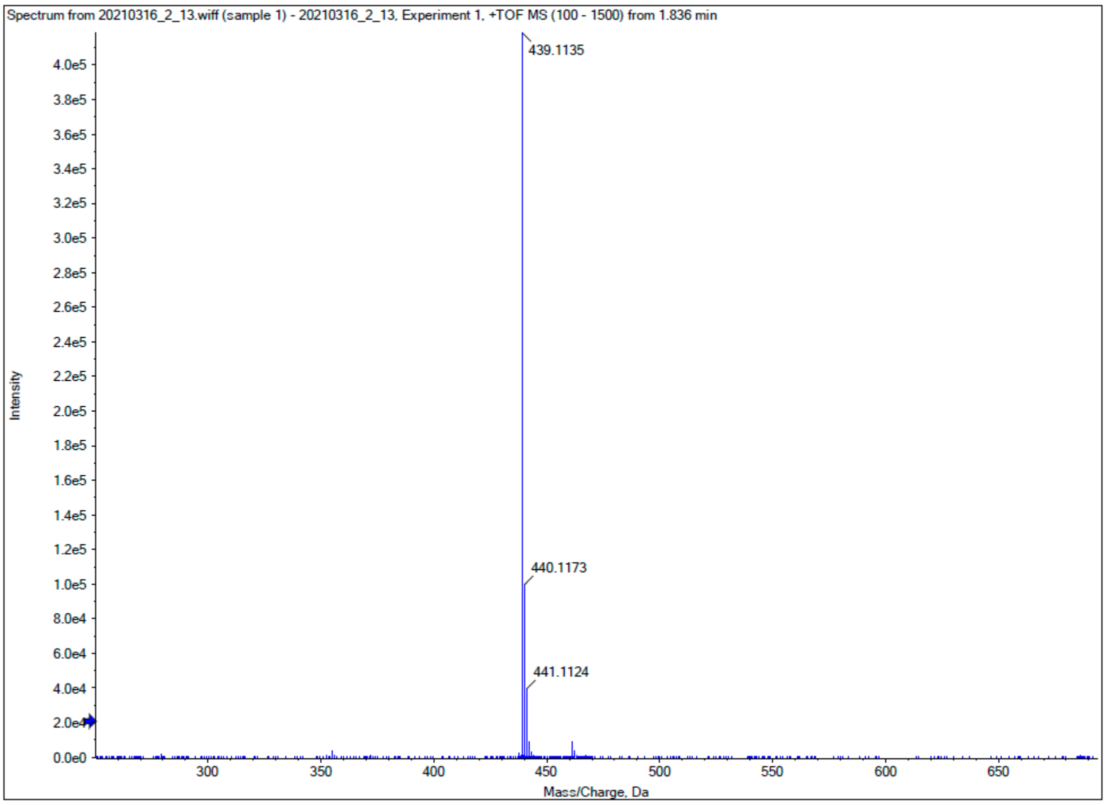
Spectrum from 20210316_2_13.wiff (sample 1) - 20210316_2_13, Experiment 1, +TOF MS (100 - 1500) from 1.836 min
439.1135
440.1173
441.1124
Intensity
Mass/Charge, Da

3i:

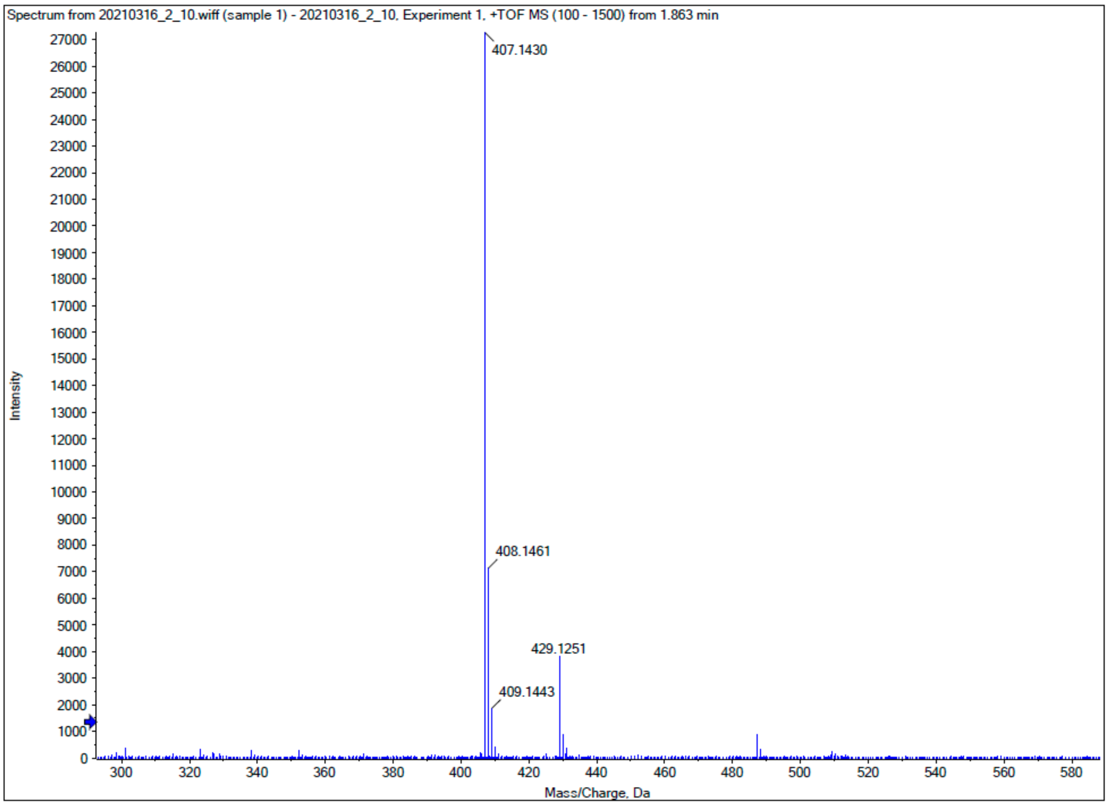

Spectrum from 20210316_2_10.wiff (sample 1) - 20210316_2_10, Experiment 1, +TOF MS (100 - 1500) from 1.863 min
407.1430
408.1461
429.1251
409.1443
Intensity
Mass/Charge, Da

3j:

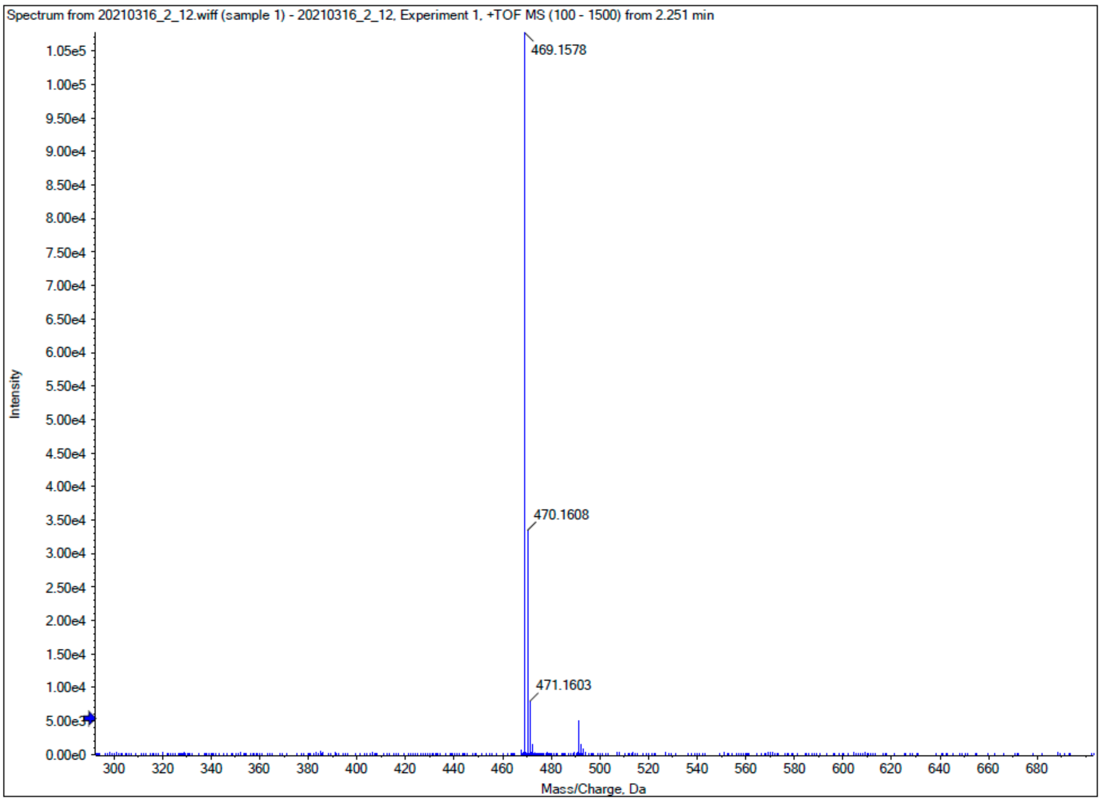

Spectrum from 20210316_2_12.wiff (sample 1) - 20210316_2_12, Experiment 1, +TOF MS (100 - 1500) from 2.251 min
469.1578
470.1608
471.1603
Intensity
Mass/Charge, Da

3k:

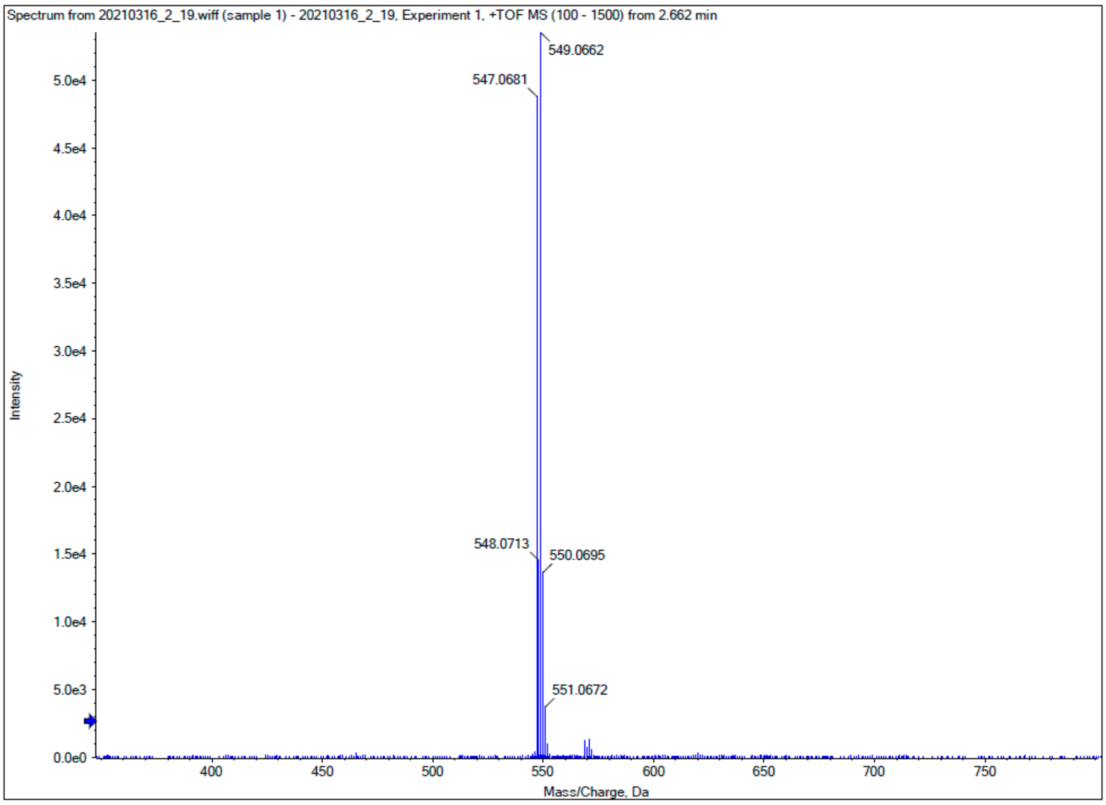
Spectrum from 20210316_2_19.wiff (sample 1) - 20210316_2_19, Experiment 1, +TOF MS (100 - 1500) from 2.662 min
549.0662
547.0681
548.0713
550.0695
551.0672
Intensity
Mass/Charge, Da

3l:

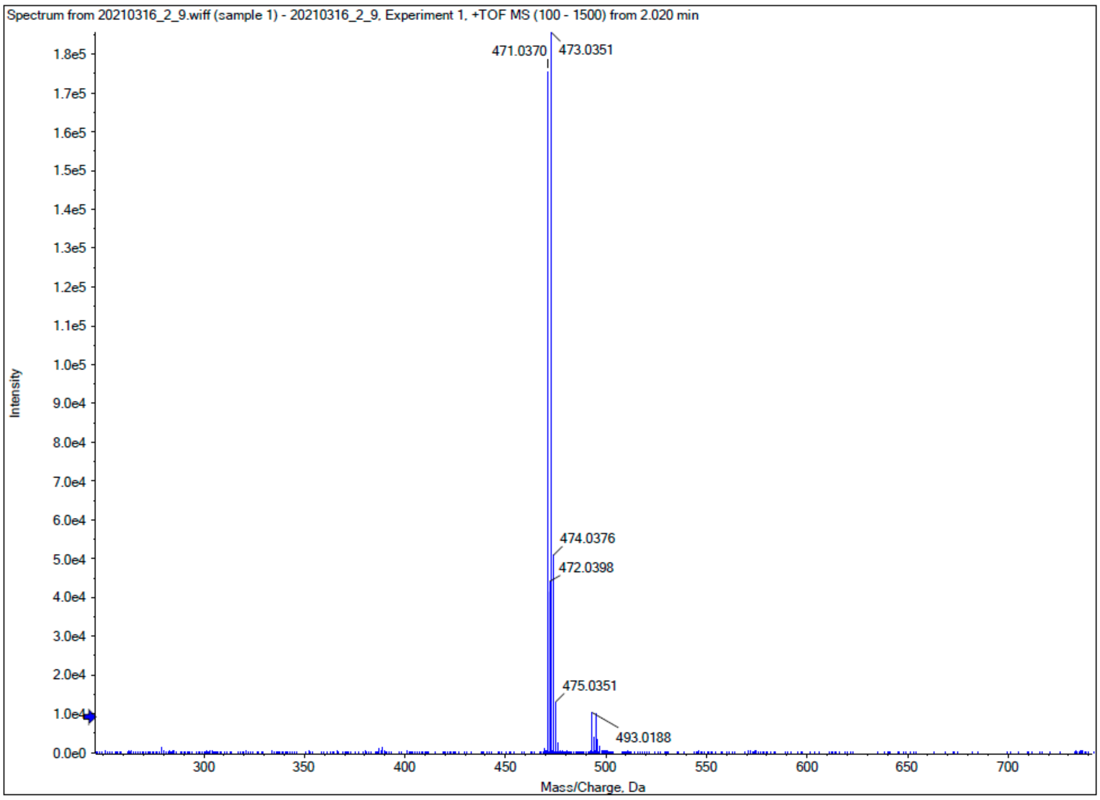
Spectrum from 20210316_2_9.wiff (sample 1) - 20210316_2_9, Experiment 1, +TOF MS (100 - 1500) from 2.020 min
471.0370
473.0351
474.0376
472.0398
475.0351
493.0188
Intensity
Mass/Charge, Da

3m:

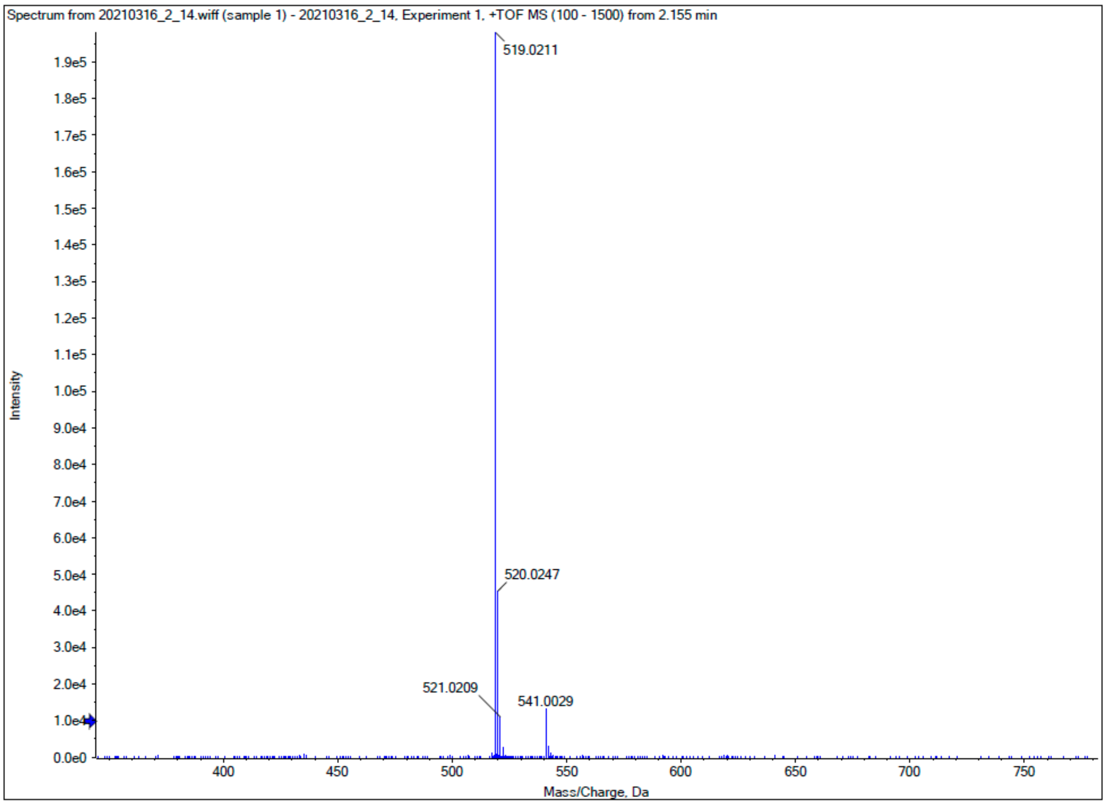
Spectrum from 20210316_2_14.wiff (sample 1) - 20210316_2_14, Experiment 1, +TOF MS (100 - 1500) from 2.155 min
519.0211
520.0247
521.0209
541.0029
Intensity
Mass/Charge, Da

3n:

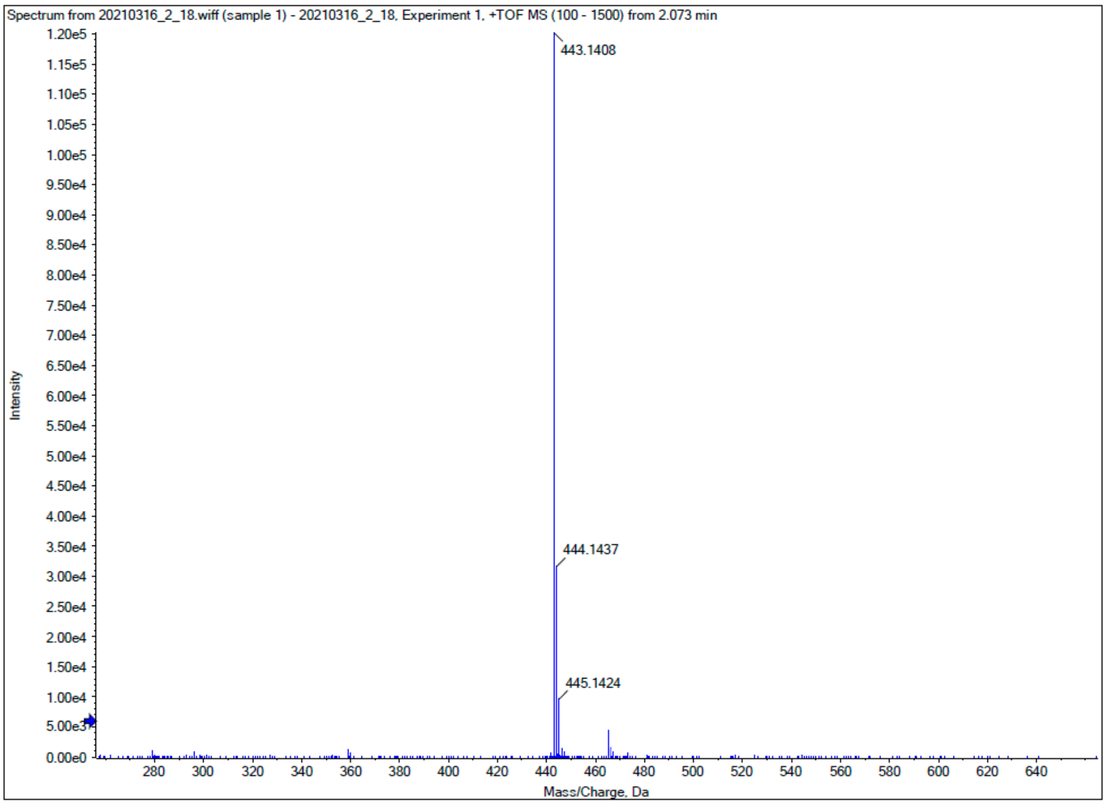
Spectrum from 20210316_2_18.wiff (sample 1) - 20210316_2_18, Experiment 1, +TOF MS (100 - 1500) from 2.073 min
443.1408
444.1437
445.1424
Intensity
Mass/Charge, Da

3o:

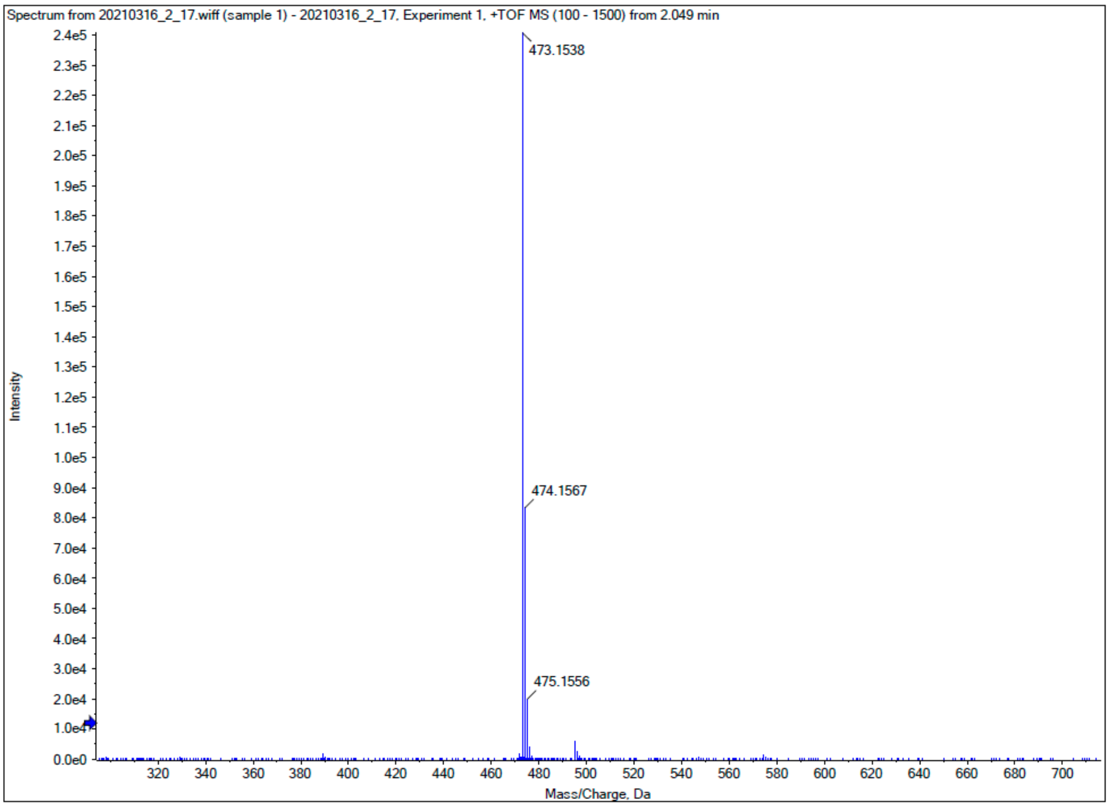
Spectrum from 20210316_2_17.wiff (sample 1) - 20210316_2_17, Experiment 1, +TOF MS (100 - 1500) from 2.049 min
473.1538
474.1567
475.1556
Intensity
Mass/Charge, Da

3p:

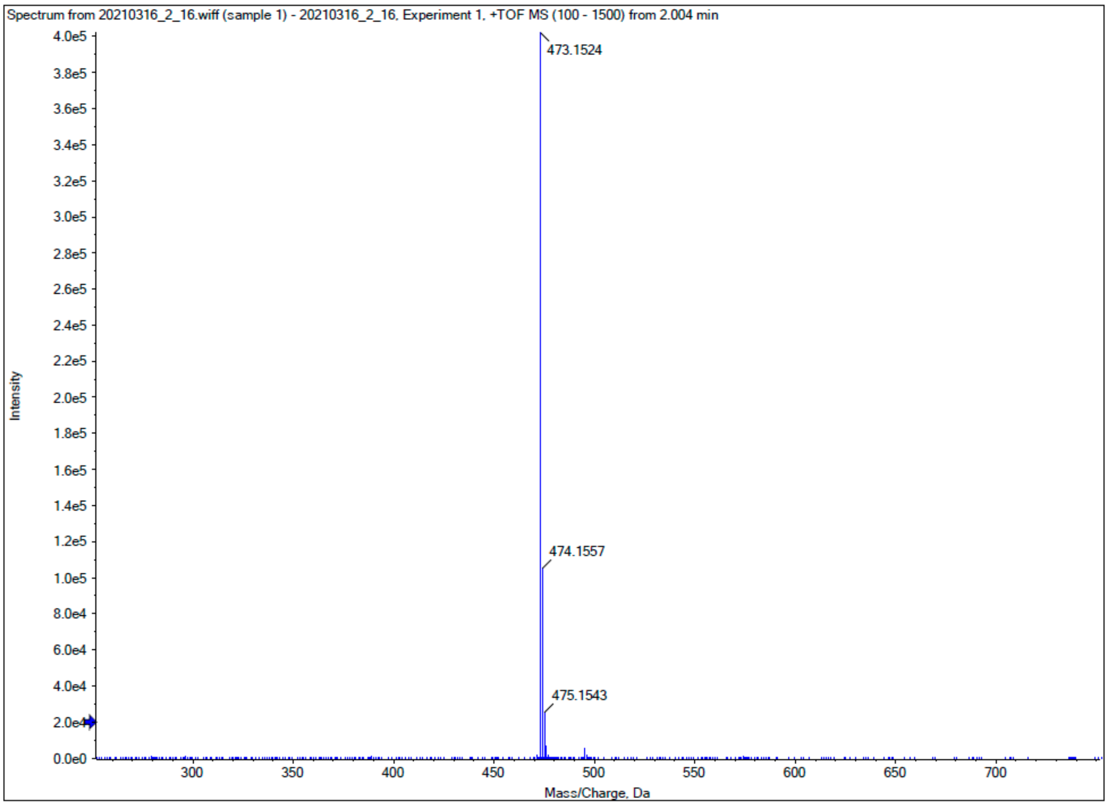
Spectrum from 20210316_2_16.wiff (sample 1) - 20210316_2_16, Experiment 1, +TOF MS (100 - 1500) from 2.004 min
473.1524
474.1557
475.1543
Intensity
Mass/Charge, Da

3q:

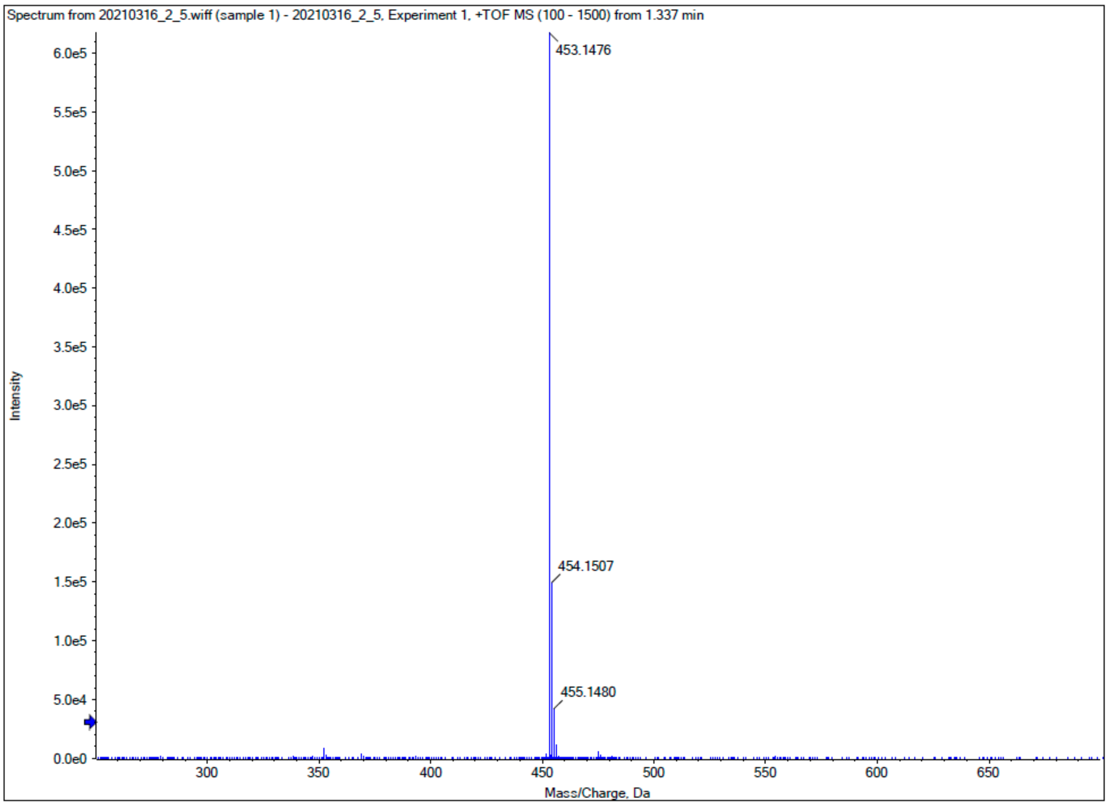
Spectrum from 20210316_2_5.wiff (sample 1) - 20210316_2_5, Experiment 1, +TOF MS (100 - 1500) from 1.337 min
453.1476
454.1507
455.1480
Intensity
Mass/Charge, Da

3r:

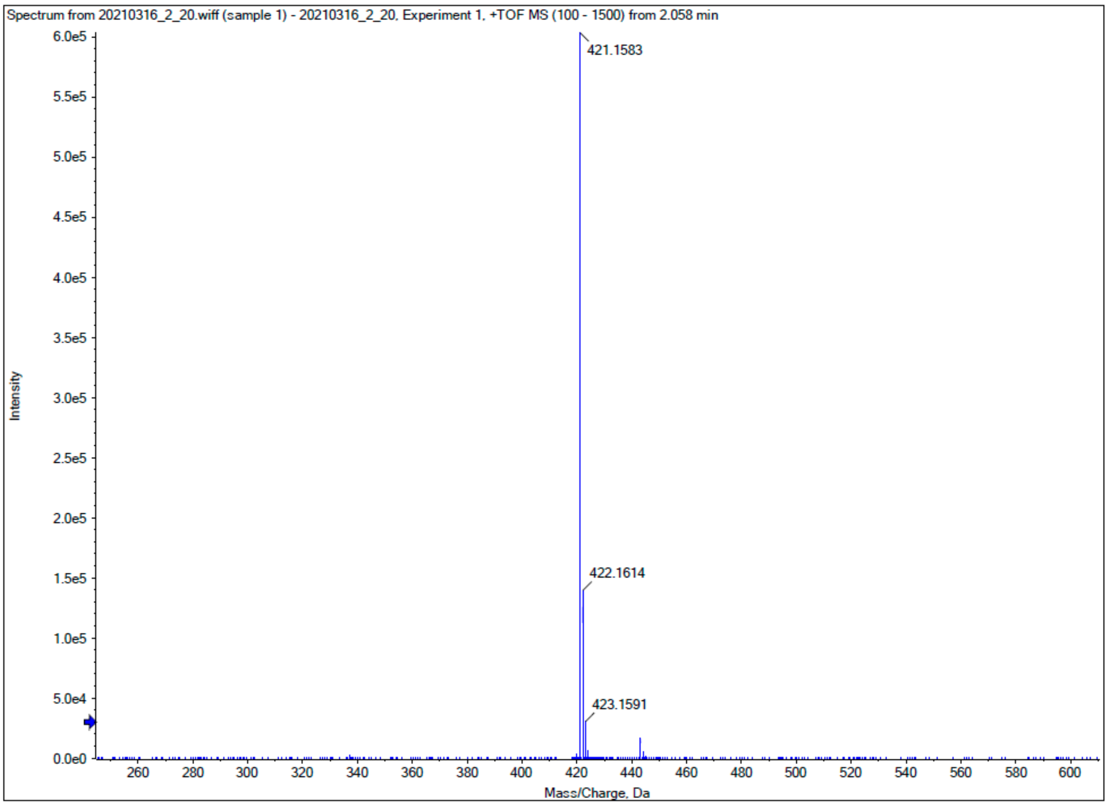
Spectrum from 20210316_2_20.wiff (sample 1) - 20210316_2_20, Experiment 1, +TOF MS (100 - 1500) from 2.058 min
421.1583
422.1614
423.1591
Intensity
Mass/Charge, Da

3s:

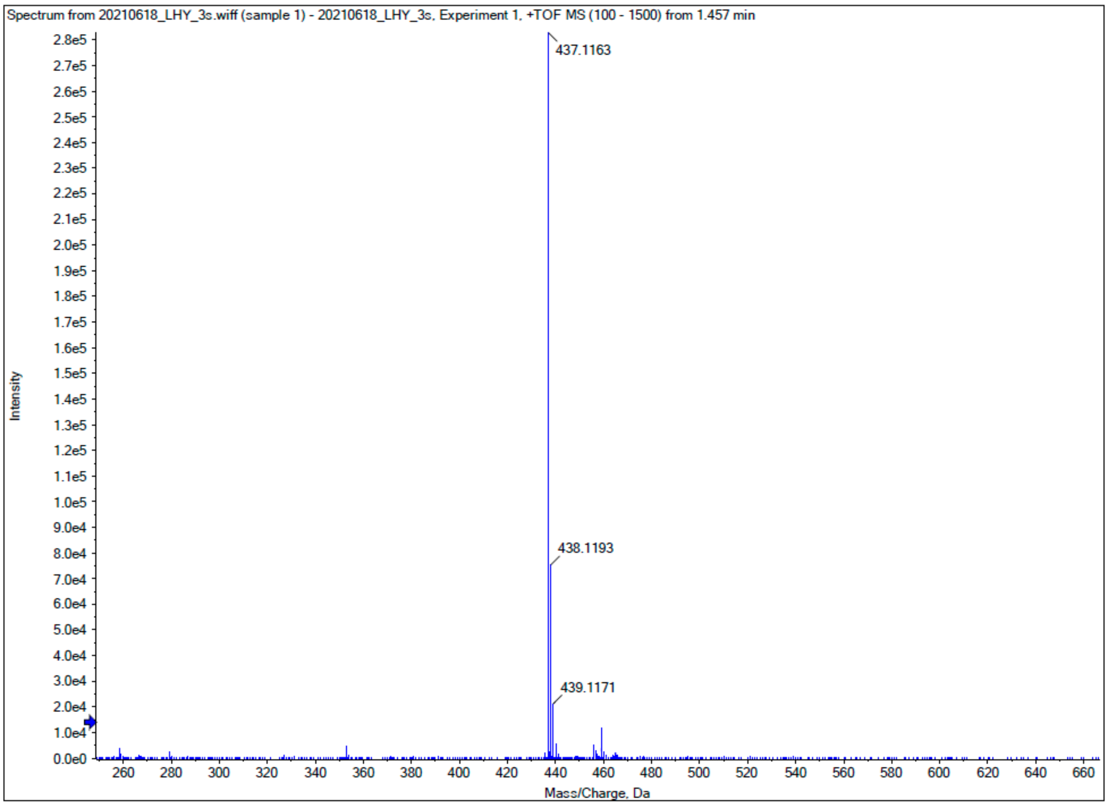
Spectrum from 20210618_LHY_3s.wiff (sample 1) - 20210618_LHY_3s, Experiment 1, +TOF MS (100 - 1500) from 1.457 min
437.1163
438.1193
439.1171
Intensity
Mass/Charge, Da

3t:

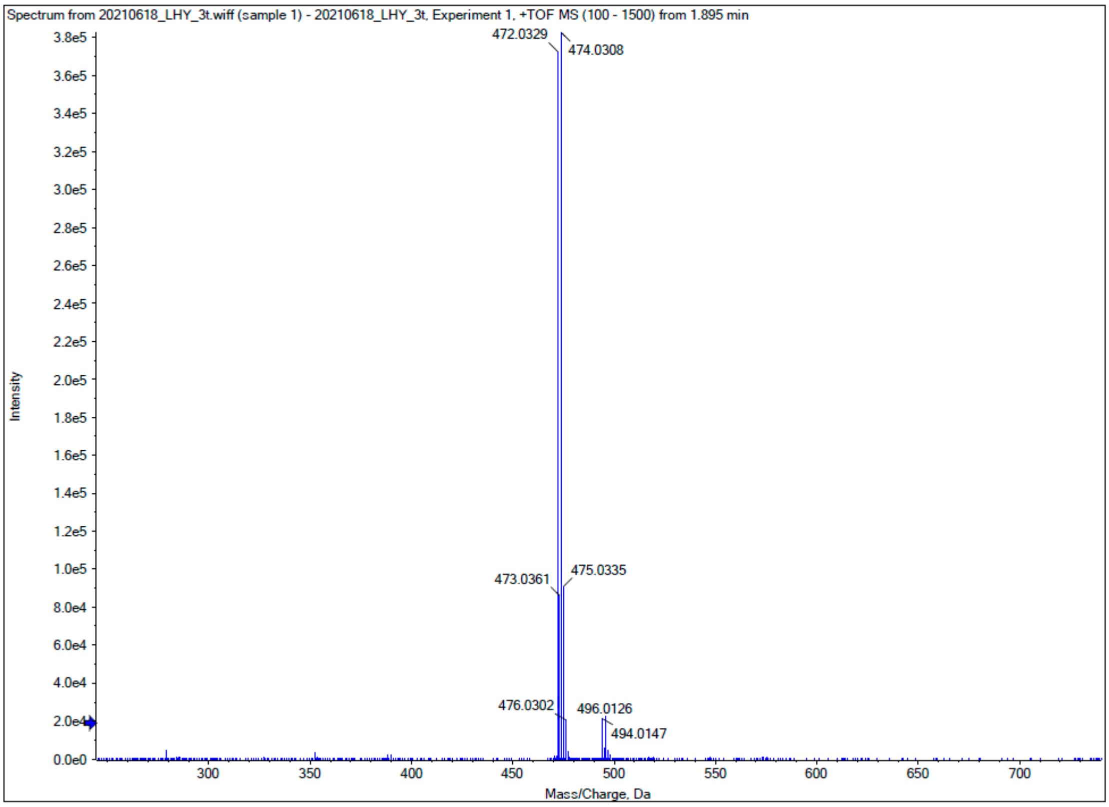
Spectrum from 20210618_LHY_3t.wiff (sample 1) - 20210618_LHY_3t, Experiment 1, +TOF MS (100 - 1500) from 1.895 min
472.0329
474.0308
473.0361
475.0335
476.0302
496.0126
494.0147
Intensity
Mass/Charge, Da
